# Relandscaping the Gut Microbiota with a Whole Food: Dose–Response Effects to Common Bean

**DOI:** 10.3390/foods11081153

**Published:** 2022-04-15

**Authors:** Tymofiy Lutsiv, John N. McGinley, Elizabeth S. Neil-McDonald, Tiffany L. Weir, Michelle T. Foster, Henry J. Thompson

**Affiliations:** 1 Cancer Prevention Laboratory, Colorado State University, Fort Collins, CO 80523, USA; tymofiy.lutsiv@colostate.edu (T.L.); john.mcginley@colostate.edu (J.N.M.); elizabeth.neil@colostate.edu (E.S.N.-M.); 2 Graduate Program in Cell and Molecular Biology, Colorado State University, Fort Collins, CO 80523, USA; tiffany.weir@colostate.edu (T.L.W.); michelle.foster@colostate.edu (M.T.F.); 3 Department of Food Science and Human Nutrition, Colorado State University, Fort Collins, CO 80523, USA

**Keywords:** common bean, microbiome, gut microbiota, cecal microbiota, microbiota accessible carbohydrates, biological sex, dose response, food pharmacology

## Abstract

Underconsumption of dietary fiber and the milieu of chemicals with which it is associated is a health concern linked to the increasing global burden of chronic diseases. The benefits of fiber are partially attributed to modulation of the gut microbiota, whose composition and function depend on the amount and quality of microbiota-accessible substrates in the diet. However, not all types of fiber are equally accessible to the gut microbiota. *Phaseolus vulgaris* L., or common bean, is a food type rich in fiber as well as other prebiotics posing a great potential to positively impact diet-microbiota-host interactions. To elucidate the magnitude of bean’s effects on the gut microbiota, increasing doses of common bean were administered in macronutrient-matched diet formulations. The microbial communities in the ceca of female and male mice were evaluated via 16S rRNA gene sequencing. As the bean dose increased, the Bacillota:Bacteroidota ratio (formerly referred to as the Firmicutes:Bacteroidetes ratio) was reduced and α-diversity decreased, whereas the community composition was distinctly different between the diet groups according to β-diversity. These effects were more pronounced in female mice compared to male mice. Compositional analyses identified a dose-responsive bean-induced shift in microbial composition. With an increasing bean dose, Rikenellaceae, *Bacteroides*, and RF39, which are associated with health benefits, were enhanced. More taxa, however, were suppressed, among which were *Allobaculum*, *Oscillospira*, *Dorea*, and *Ruminococcus*, which are predominantly associated with chronic disease risk. Investigation of the origins of the dose dependent and biological sex differences in response to common bean consumption may provide insights into bean-gut microbiota-host interactions important to developing food-based precision approaches to chronic disease prevention and control.

## 1. Introduction

Chronic non-communicable diseases, such as obesity, type-2 diabetes mellitus, cardiovascular disease, and cancer, account for greater than 70% of global deaths per annum [1]. Among the risk factors associated with their intricate and multifaceted etiology, poor diet has long been considered to play a role in these disease processes [2,3]. While the emphasis of current epidemiological investigations and of food policy decision-makers is on foods and the pattern in which they are typically eaten [4,5], dietary guidelines, such as U.S. Dietary Guidelines for Americans (DGA), as well as those published in other countries and by various organizations therein [6,7], continue to identify nutrients and dietary factors of concern because they are typically either underconsumed, or are consumed in excess, and are therefore associated with disease risk [7,8,9,10].

Fiber, and the milieu of chemicals with which it is associated, is a dietary factor of concern due to underconsumption in developed countries and in developing countries transitioning to Westernized food patterns [6,11,12]. Dietary fiber is a type of carbohydrate whose chemical components vary among food categories and that is either slowly digested or is indigestible by monogastric animals including humans. Consequently, dietary fiber is a fuel source for the intestinal microbiota, and many of the health benefits associated with dietary fiber ingestion are attributed, in particular, to them. Several mediators of such benefits have been identified, including metabolites of bacterial fermentation, such as short-chain fatty acids, polyphenol derivatives, lower pH, and mucus layer protection [13,14,15]. However, dietary fiber encompasses various compounds with different functionality, and the food source thereof plays an important role as well. As recently noted in the 2020–2025 DGA [7], and as underscored by us [16,17], one particular category of foods rich in dietary fiber that was once widely consumed and that has essentially disappeared from the food patterns typical of developed countries is pulses.

Pulses are grain legumes unique in their high content of both fiber and protein, as well as concomitant absence of fat [17]. On a per 100 kcal edible portion basis, whole pulses provide more dietary fiber than any other food category. Thus, the increased consumption of pulses, such as chickpea, dry pea, lentil, and the most commonly consumed among them—common bean, has the potential to close the dietary fiber gap by improving dietary quality while decreasing the energy density of a food pattern [18,19]. Food guidelines on legume consumption differ across the countries: while Europe recommends over 450 g of legumes per week, the United States recommends less than 45 g total weekly [6]. Perusal of the scientific literature reveals a growing number of systematic reviews and meta-analyses describing inverse relationships between pulse consumption and incidence of chronic non-communicable diseases [20,21,22]. These reports are consistent with our findings that pulse consumption has anti-obesogenic activity in a rat model of polygenic obesity and in a mouse model of dietary-induced obesity where it also confers microbiota-modulating effects [23,24,25,26,27,28]. However, what is missing from the literature is an assessment of how the gut bacteria respond to increasing amounts of pulse in the diet and whether the biological sex of the organism has any effect on the observed responses.

Common bean, or *Phaseolus vulgaris* L., is the most widely eaten type of pulse along with being consumed in the greatest quantities globally, and therefore was chosen to study effects on the cecal microbial composition of mice. Our main objectives were: (1) to analyze changes to cecal bacteria composition with an increasing dose of common bean in the diet; and (2) to determine whether the biological sex of the mice has an effect on those changes. Following the recent changes in the nomenclature of bacterial taxonomy [29], we adopted herein the new names of phyla, e.g., Firmicutes were renamed to Bacillota, Bacteroidetes—to Bacteroidota, Verrucomicrobia—to Verrucomicrobiota, etc.; as well as updated names since the last version (13_8) of Greengenes reference database, such as family S24-7 was updated to name Muribaculaceae [30]. Despite translatability tissues of the gut microbiota studies in the murine models [31,32], our focus was the scope of the effects of bean consumption on shifts in the bacterial populations in the cecum. Since the intestinal microorganisms comprise only one of the elements of systemic physiologic changes induced by the diet, the findings obtained in the current study correspond to previous and future studies on mice in the realm of dietary effects on the etiology of chronic diseases.

## 2. Materials and Methods

### 2.1. Study Design

All animal-related work was performed in compliance with the Colorado State University Institutional Animal Care and Use Committee (protocol 18-7746A-KP 1431). Eighty female and eighty male wildtype C57BL6/J mice (stock# 000664) were purchased from The Jackson Laboratory (Barr Harbor, ME, USA) at 20 days of age. Mice were housed in solid bottomed polycarbonate rodent cages and maintained on a 12 h light/dark cycle at 27.5 ± 2 °C with 30% relative humidity and *ad libitum* access to distilled water. As a model for obesity, mice were adapted to a purified rodent diet formulation (with 32.5% dietary kcal derived from fat) and animal husbandry routine until they reached 8 weeks of age. This approach promotes dysbiosis that is associated with the obesity phenotype, which manifests beginning 8 weeks of age [33]. At that time, animals were randomized based on their body weight to experimental diet groups (*n* = 20 of each sex per diet group) and fed their respective experimental diet formulations for 12–14 weeks (see Section 2.2). Animals were euthanized by cervical dislocation following isoflurane-induced anesthesia. The cecum was harvested, cut open, and contents gently mixed with a clean spatula to produce a homogeneous mixture, which was placed in a sterile cryovial and snap-frozen in liquid nitrogen for subsequent DNA extraction. Cecal samples from animals within each diet group with a similar weight gain pattern were pooled (*n* = 2 per pool). Thus, each diet group was comprised of 10 pooled cecal samples total.

### 2.2. Experimental Diets

Purified diets were formulated to provide 32.5% of dietary kcal from fat to induce excessive weight gain in C57BL6/J mice [34]. Freeze dried powder from whole cooked white kidney bean was incorporated into the diet by substituting casein with 0%, 17.5%, 35%, and 70% of the protein derived from bean. Despite increasing doses of the bean, adjustments were made in other components so that macronutrient content was similar across diet groups (Table 1). 

### 2.3. 16S rRNA Gene Library Preparation, Sequencing, and Processing

From the cecal content, DNA was extracted with the QIAamp PowerFecal DNA kit (Qiagen, Germantown, MD, USA) according to the manufacturer’s protocol, and its purity and concentration were determined by NanoDrop (Thermo Fisher Scientific, Waltham, MA, USA). The V4 region of the 16S rRNA gene was amplified and sequenced to construct paired-end sequencing libraries using the 515F-806R primer set following the Earth Microbiome Project protocols [35], followed by sequencing using the MiSeq Reagent Kit v2 2 × 250 bp on an Illumina MiSeq instrument housed at the Next-Generation Sequencing Facility at Colorado State University. 

Bioinformatic processing of the obtained forward and reverse paired-end sequence reads was performed with QIIME 2 platform, version 2021.8 [36]. Raw sequence data were demultiplexed and quality-filtered via q2-demux plugin. Denoising was performed with the DADA2 pipeline (q2-dada2) [37]: each sequence pair was truncated from 225 bp (forward) and 155 bp (reverse), checked for chimeras, and filtered for quality control. Taxonomy was assigned to the resulting amplicon sequence variants (ASVs) using a Naive Bayes classifier (via q2-feature-classifier plugin [38]) pre-trained on Greengenes (16S rRNA, version 13_8)—a marker gene reference database trimmed to the V4 domain (bound by the 515F/806R primer pair) with a 99% sequence identity threshold [39]. The dataset was filtered to remove all features annotated as “mitochondria” and “chloroplast.” A rooted phylogenetic tree was built using FastTree [40] and MAFFT [41] alignment via q2-phylogeny plugin. 

### 2.4. Statistical and Bioinformatic Analyses

Further analysis was conducted in QIIME 2, MicrobiomeAnalyst (Marker-gene Data Profiling module) [42,43], and R (version 4.1.1, R studio version 2021.09.0) on two output datasets: the ASVs abundance dataset and their taxa-assigned abundance dataset. The latter comprised a list of identified bacteria mapped to the lowest possible taxonomic level. Certain bacteria were underclassified and thus marked “I” if there was insufficient resolution to annotate to species (indicated with “s_” in the Greengenes database); or marked “II” if it was impossible to classify the ASVs to a deeper level (indicated as “_” in the Greengenes database). Features (ASVs or taxa) with less than two counts were automatically removed by pre-processing steps of MicrobiomeAnalyst’s integral Sanity Check. As a result, the female cohort dataset consisted of 233 ASVs, whereas the male cohort dataset included 220 ASVs. 

Using q2-diversity plugin in QIIME 2, the samples were rarefied (subsamples without replacements) to 21,418 (female cohort) and 23,617 (male cohort) sequences per sample, retaining 74.23% and 78.93% features (the maximally available option), respectively. Two samples from each sex cohort did not meet the sampling depth threshold and were excluded. Richness and evenness were analyzed using Observed Features, Faith’s phylogenetic diversity, and Shannon’s diversity index metrics, then statistically compared between the diet groups via the Kruskal-Wallis non-parametric test (both in QIIME 2) and visualized using R. Principal coordinates analysis (PCoA) of β-diversity matrices was based on Jaccard dissimilarity as well as phylogeny-based Weighted and Unweighted UniFrac distances, and were assessed using permutational multivariate analysis of variance (PERMANOVA) with 999 permutations in QIIME 2, and the results were visualized in MicrobiomeAnalyst.

For compositional analyses, the taxa-assigned datasets were further subjected to quality filtering and normalization in MicrobiomeAnalyst. Taxa were filtered for low abundance—a minimum of 10% samples with at least four counts were retained—as well as for the low variance—less than 5% based on the inter-quantile range were removed. Total sum scaling (TSS) was performed to normalize the data.

The Heat Tree Analysis was used to utilize the hierarchical structure of taxonomic classifications to quantitatively (using the median abundance) and statistically (using the non-parametric Wilcoxon Rank Sum test) depict taxonomic differences between microbial communities between the sex cohorts via Reingold-Tilford layout using R metacoder package in MicrobiomeAnalyst [44].

Bacterial biomarkers were discovered using the linear discriminant analysis (LDA) effect size (LEfSe) method [45]. This algorithm allowed detection of differentially abundant taxa among the experimental groups using the Kruskal-Wallis test and then evaluated their relevance via an LDA score. LEfSe was performed on the phylum and the feature levels of the taxa-assigned dataset using cutoffs of <0.05 for the FDR-adjusted *p*-value and >2.0 for the logarithmic LDA score. Analyses were performed within sex cohorts as well as in the pairwise comparison setting to assess differences between the diet groups.

Variation of taxonomic abundance related to the diet group was visualized on a heatmap in MicrobiomeAnalyst after Ward’s hierarchical clustering algorithm based on Minkowski distances. Feature level was used for the analysis of the taxa-assigned dataset.

Correlation analysis was performed to build correlograms between bacterial relative abundance pairs and the bean dosage using Spearman’s rank-order correlation testing. Correlations with a *p*-value < 0.05 were included in the results. 

Functional attributes of the identified microbial communities were predicted using Phylogenetic Investigation of Communities by Reconstruction of Unobserved States 2 (PICRUSt2) pipeline, version 2.4.1 [46]. With the ASV dataset as an input, PICRUSt2 performs phylogenetic placement by aligning ASVs to the reference 16S sequences (HMMER, www.hmmer.org, accessed on 1 December 2021) and incorporating them into the reference tree evolutionary placement algorithm (EPA)-NG and genesis applications for phylogenetic placement analysis (GAPPA) [47,48], followed by the hidden-state prediction of gene families (castor R package [49]) and, finally, generation of metagenomic predictions and tabulation of pathways’ inferences and abundances (Minimal set of Pathways (MinPath)) [50] and MetaCyc [51]. Statistical analysis of taxonomic and functional profiles (STAMP) software, version 2.1.3 (Robert Beiko, Halifax, NS, Canada), was used to analyze and visualize PICRUSt2 output data [52]. In brief, the pulse-free Control group and samples from pulse-based diet groups were compared using Welch’s two-sided *t*-test with 0.95 Welch’s inverted CI method and the Benjamini-Hochberg FDR multiple test corrections method.

## 3. Results

### 3.1. Overview of the Bean-Induced Microbial Community in the Cecum

**Figure 1:**
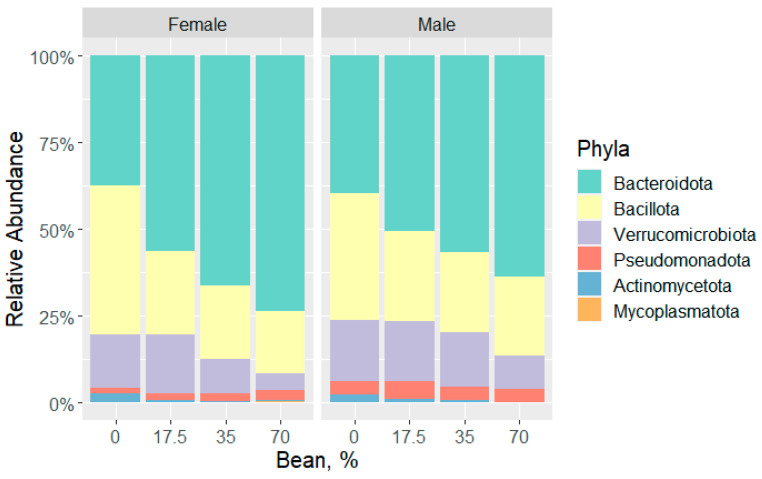
Relative abundance of identified phyla across the diet groups of each sex cohort.

Analysis of bacteria at the phylum level allows for the detection of the most marked statistical differences between the experimental groups. Overall, six phyla were identified in the ceca of mice fed with or without increasing amounts of common bean. In light of recent changes in microbiological nomenclature [29], new phyla names are reported here. Each diet group demonstrated a distinct microbial profile (Figure 1). Across all samples, the most prevalent phyla were the Gram-positive Bacillota (formerly Firmicutes) and the Gram-negative Bacteroidota (formerly Bacteriodetes); however, there is a significant increase in Bacteriodota and subsequent decrease in Bacillota and Actinomycetota (formerly Actimomycetes) with increasing bean dose. 

Although the dose response trends were similar across male and female mice, there were some sex-specific changes. In the female cohort, the bean-free control diet exhibited the Bacillota:Bacteroidota ratio (a Firmicutes:Bacteroidetes ratio) of 1.154, with Bacillota being the most abundant phylum (43.03%), followed by Bacteroidota (37.3%), Verrucomicrobiota (15.4%), and Actinomycetota (2.72%). As bean was introduced to the diet, the Bacillota:Bacteroidota ratio decreased with the increasing dose (0.421–0.232), with Bacteroidota dominating in the cecum (56.65–74.12%), followed by Bacillota (23.85–17.23%), Verrucomicrobiota (16.95–4.94%), and Pseudomonadota (formerly Proteobacteria; 1.77–3.14%) as the bean dose increased. 

In contrast, the Bacteroidota was dominant in male mice prior to the introduction of bean, with a Bacillota:Bacteroidota ratio of 0.922 in the control group and a range of 0.528–0.360 as the bean dose increased. The most abundant phyla were Bacteroidota (39.35–63.47%), Bacillota (36.29–22.88%), Verrucomicrobiota (18.02–9.62%), and Pseudomonadota (3.83% in the control and bean dose-dependent decrease from 5.24% to 3.81%). Therefore, despite similar patterns of change in phyla abundance, the male mice were less impacted by bean introduction than female mice. 

For more quantitative analyses of differences in bacterial communities across the diet groups, the relative abundances were subjected to Kruskal-Wallis testing with a *post hoc* Dunn test for pairwise comparisons, and the results are summarized in Table 2. Starting at the 17.5% bean group, the relative abundance of Bacillota decreased by 19.18%, and then by 22.26% and 25.8% in the 35% and 70% groups, respectively, among the female cohort. Less drastically in males, Bacillota decreased by 10.14%, 12.74%, and 13.41% along with the increasing bean dose in the diet groups. Starting at 35% bean, Actinomycetota decreased by 2.56% and 2.63% compared with the control group in females, and by 1.96% and 2.39%, respectively, in males. An increase in Bacteroidota upon bean consumption is observed starting at 35% bean in both sex cohorts (by 29.17–36.82% increase in females and 16.97–24.12% increase in males). 

### 3.2. Analysis of α- and β-Diversity among the Diet Groups

**Figure 2:**
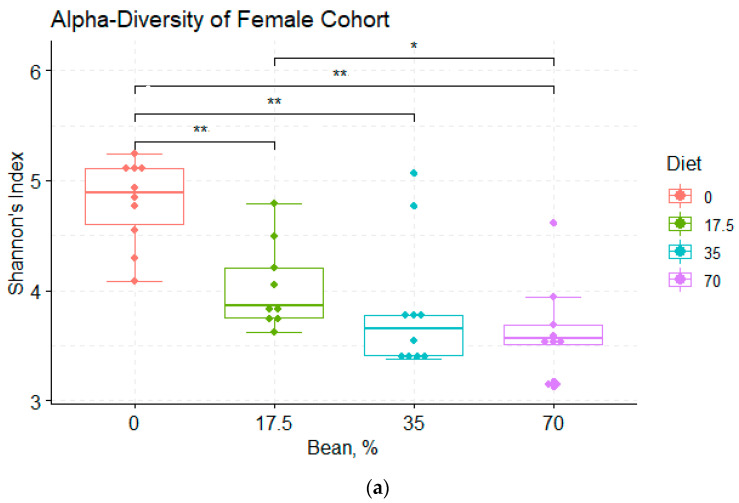

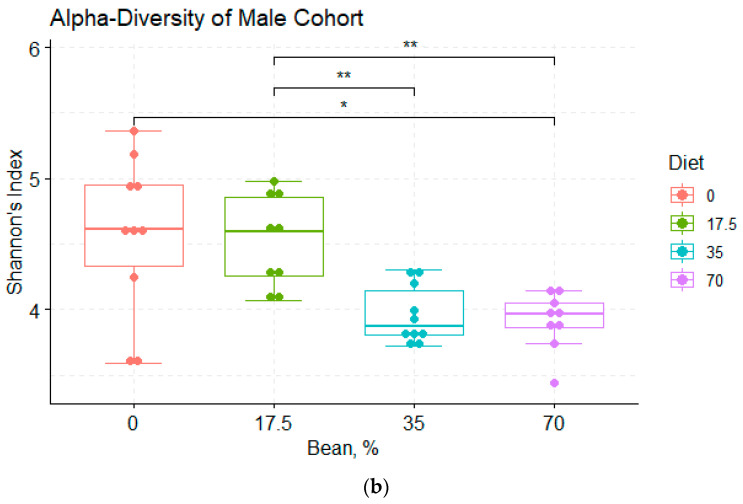
(**a**) Shannon’s indices of α-diversity of the female cohort. Statistically significant differences were determined by the Kruskal-Wallis test (*p*-value < 0.001) with * *q*-value < 0.05; ** *q*-value < 0.01; (**b**) Shannon’s indices of α-diversity of the male cohort. Statistically significant differences were determined by the Kruskal-Wallis test (*p*-value = 0.002) with * *q*-value < 0.05; ** *q*-value < 0.01.

Bean incorporation into the diet decreased the diversity of the cecal microbiota within samples. Observed richness, as well as Faith’s phylogenetic diversity, were significantly higher in the bean-free control group compared with each bean-containing diet, but this was not observed in their male counterparts (Figures S1–S2). When α-diversity was assessed using the Shannon index (Figure 2), which accounts for both ASVs richness and evenness within samples, the 70% bean diet group was statistically lower than the control diet in male mice, whereas all bean-based diet groups had significantly reduced diversity from the control diet in female animals. Looking at the uncorrected *p*-values, the males fed the 35% bean group also differed from the control with *p*-value = 0.034 (correction for multiple comparisons showed only a statistical trend, *q*-value = 0.052). Females responded to changes in their gut microbiome at the lowest dose of bean compared to their male counterparts in terms of Shannon’s indices. Each diet group was also assessed for α-diversity differences in female versus male cohorts (Figure S3). Only Faith’s phylogenetic richness was higher in males compared to the female sample cohort, while according to the other metrics, there were not statistically significant differences despite the tendency for higher intra-sample diversity in males. 

**Figure 3:**
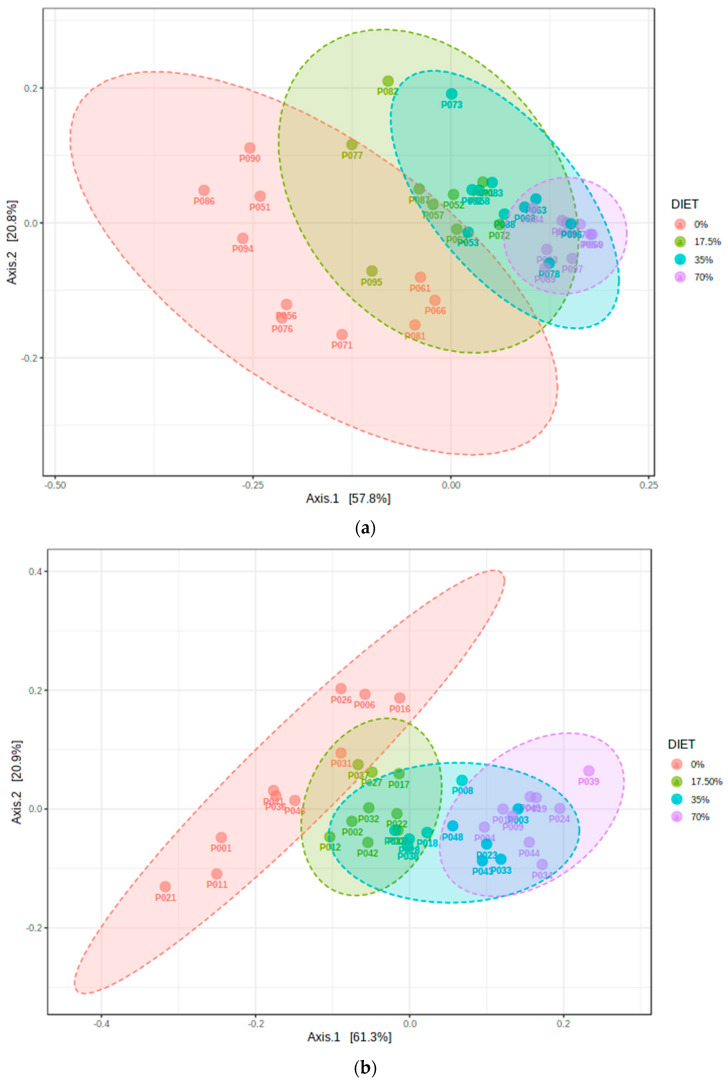
Phylogenetic Weighted UniFrac distances were used to explain β-diversity across diet groups. Differences between the samples were tested by the permutational multivariate analysis of variance (PERMANOVA): samples are colored and grouped by their corresponding diet group into ellipses representing 95% confidence intervals. (**a**) in the female cohort, pseudo-*F*-value = 12.845; *p*-value < 0.001. All diet groups were statistically different from one another; (**b**) in the male cohort, pseudo-*F*-value = 14.751; *p*-value < 0.001. All diet groups were statistically different from one another.

Several measures of β-diversity also suggested that the control diets resulted in a microbial profile distinct from the microbiota of the bean-containing diet groups. As the bean dose increased in the diet, the samples localized further away from the control group in the PCoA matrix. Pairwise PERMANOVA analysis indicated that all bean-based diets significantly differed from the bean-free control (*p* < 0.01). All diet groups significantly differed from one another when Jaccard distances were subjected to PCoA (Figure S4), highlighting the qualitative absence and presence of unique ASVs in both sex cohorts. Similar results were obtained when phylogenetic relationships were used to generate the distance matrices. There was a clear separation of diet groups in ordinations of both weighted (Figure 3) and unweighted (Figure S5) UniFrac distance matrices. Therefore, bean-induced differences in the cecal microbial communities among the samples were detected even at the lowest tested dose (17.5%). 

### 3.3. The Differential Abundance Analyses

**Figure 4:**
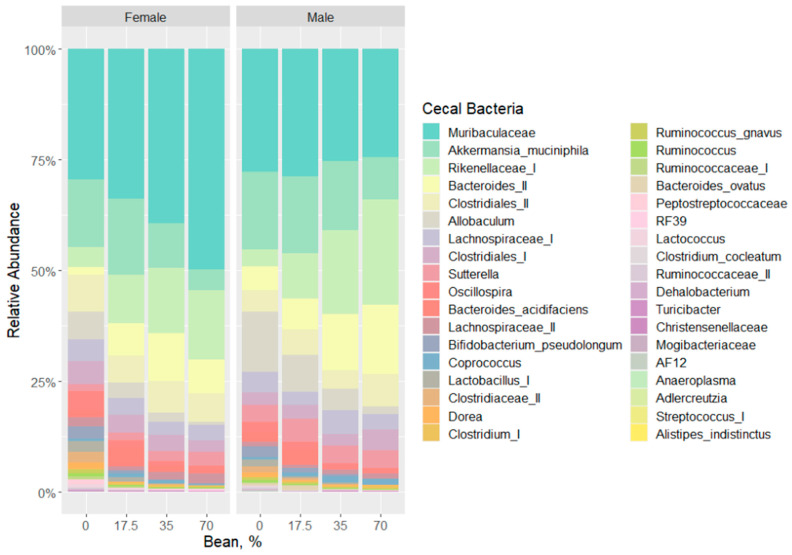
Relative abundance of cecal bacteria at their lowest level of identified taxonomy across the diet groups of each sex cohort.

**Figure 5:**
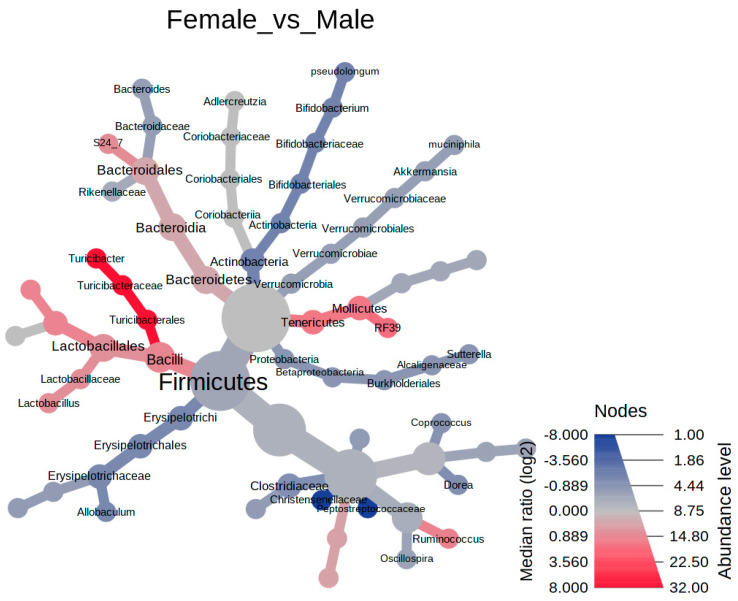
The Heat Tree analysis of taxonomic changes in the female versus male cohorts. The color corresponds to the log2 ratio of median proportions of taxa observed in each cohort across only bean-containing diet groups. Only significant differences are colored according to the Wilcoxon rank-sum testing. Nomenclature used herein is according to the Greengenes database.

Observed changes in the cecal microbiota from the aforementioned analyses revealed that female and male mice respond differently to the introduction of whole beans into the dietary formulation. Figure 4 displays the relative abundance (post filtering) of detected cecal bacteria at their lowest possible taxonomic level across all tested diet groups. Muribaculacease, *Akkermansia muciniphila*, Rikenellaceae (I), *Bacteroides (II)*, Clostridiales (II) comprised on average the top 5 most abundance bacterial taxa. To better visualize the distribution of the microbiota in our samples, we constructed a hierarchical clustering heatmap. Figure S6 displays that regardless of sex, the bean-free control samples cluster distinctly from the bean-fed diet groups in a similar pattern. Differences between the sex cohorts are evident in each diet as well. Next, all bean-containing diet groups were subjected to the Heat Tree analysis (Figure 5) to show group-wise changes in relative abundances of the bacteria that drive sex-dependent differences upon bean consumption. Overall, Bacilli were more abundant in the female mice, as well as *Turicibacter*, *Lactococcus*, *Lactobacillus*, *Ruminococcus*, Muribaculaceae, and RF39, whereas Christensenellaceae, Peptostreptococcaceae, Clostridiaceae, *Bacteroides*, *Coprococcus*, *Sutterella*, *Allobaculum*, *B. pseudolongum*, and *A. muciniphila* were more representative of the male cohort. These sex-dependent biomarkers across all the diet groups were further confirmed via pairwise LEfSe setting (Figure S7).

**Figure 6:**
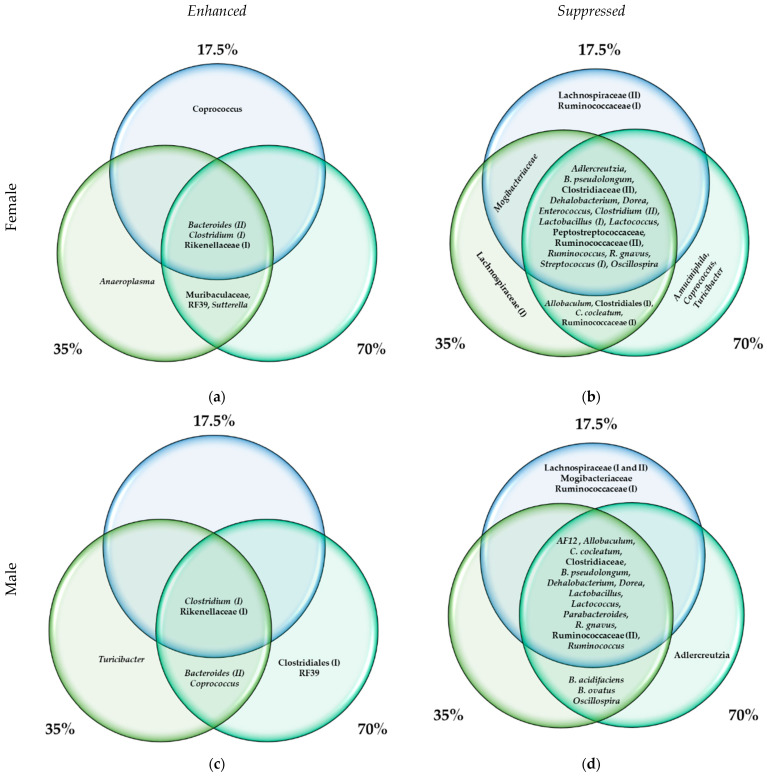
Bean-induced bacterial biomarkers of the (**a**,**c**) female and (**b**,**d**) male cohorts according to the pairwise linear discriminant analysis (LDA) of effect size (LEfSe) at the feature level. Panels (**a**,**b**) exhibit bean-enhanced, whereas (**c**,**d**) exhibit bean-suppressed bacterial biomarkers. All represented bacteria were statistically significant (LDA score > |2.0|, FDR-adjusted *p*-value < 0.05).

To determine which bacteria are the most influential in driving the differences across the diets in each sex cohort, LEfSe was performed. LEfSe indicated that 25 microbiota were statistically differential biomarkers of diet groups in the female cohort (Figure 6a,b). According to the pairwise LEfSe analyses, Rikenellaceae (I), *Bacteroides (II)*, and *Clostridium (I)* significantly increased compared with the control in all bean-based diets, whereas higher counts of Muribaculaceae, *Sutterella*, and RF39 were observed starting from the 35% dose of bean. These 6 bacterial groups comprise a bean-enhanced eco-group in the female mice (Figure 6a). Interestingly, *Coprococcus* was statistically enhanced in the 17.5% bean group versus bean-free control, but not in the higher bean dose groups. Similarly, *Anaeroplasma* was significantly different from the control only in the 35% bean group. In the male cohort, there were 28 differential bacterial biomarkers across the diet groups (Figure 6c,d). Among them, Rikenellaceae (I) and *Clostridium (I)* steadily increased in all bean-based diets compared with the control group; *Bacteroides (II)* and *Coprococcus* were enhanced starting from 35% bean dosage, whereas unclassified Clostridiales (I) and RF39 were significantly enhanced compared to the bean-free control only in the 70% bean group. These taxa form a bean-enhanced eco-group in the male mice (Figure 6c). Interestingly, in the 35% bean diet group, *Turicibacter* was also statistically different from the control and 17.5% bean groups, but not in the higher 70% dose group.

The bean-suppressed eco-group in the male samples was comprised of *Allobaculum, B. pseudolongum*, *Parabacteroides*, Clostridiaceae (II), *Lactobacillus (I)*, *Dorea*, *R. gnavus*, *Ruminococcus*, *AF12*, *C. cocleatum*, *Lactococcus*, Ruminococceae (II), and *Dehalobacterium*, which decreased in all bean-based groups versus the control; as well as *B. acidifaciens*, *Oscillospira*, and *B. ovatus*, the abundance of which declined starting from 35% bean dosage; lastly, *Adlercreutzia* were statistically different from the control only in the male 70% bean group (Figure 6d). In contrast, in the female mice, *Oscillospira*, *B. pseudolongum*, Clostridiaceae (II), *Lactobacillus (I)*, *Dorea*, Peptostreptococcaceae, *R. gnavus*, *Lactococcus*, *Ruminococcus*, Ruminococcaceae (II), *Clostridium (II)*, *Dehalobacterium*, Mogibacteriaceae, *Streptococcus (I)*, *Adlercreutzia*, *Enterococcus*, as well as—starting from 35% bean dose—*C. cocleatum*, Ruminococcaceae (I), Clostridiales (I), and *Allobaculum* create the bean-suppressed eco-group (Figure 6b). Additionally, *Turicibacter*, *Coprococcus*, and *A. muciniphila* were significantly decreased in the 70% bean group compared with both the 0% and 17.5% bean groups. *Coprococcus* was not included in the bean-suppressed female eco-group due to its significantly increased abundance in the 17.5% versus 0% bean group comparison. 

**Figure 7:**
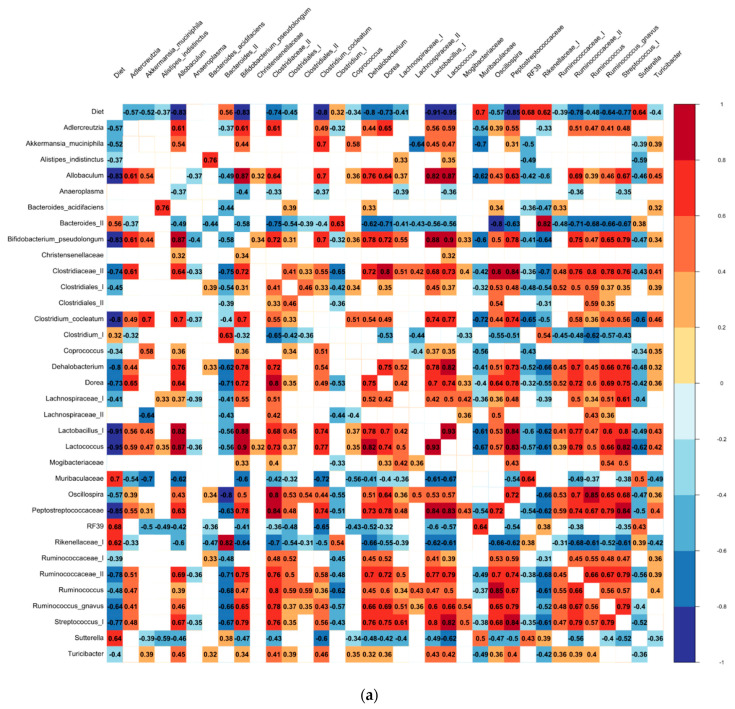

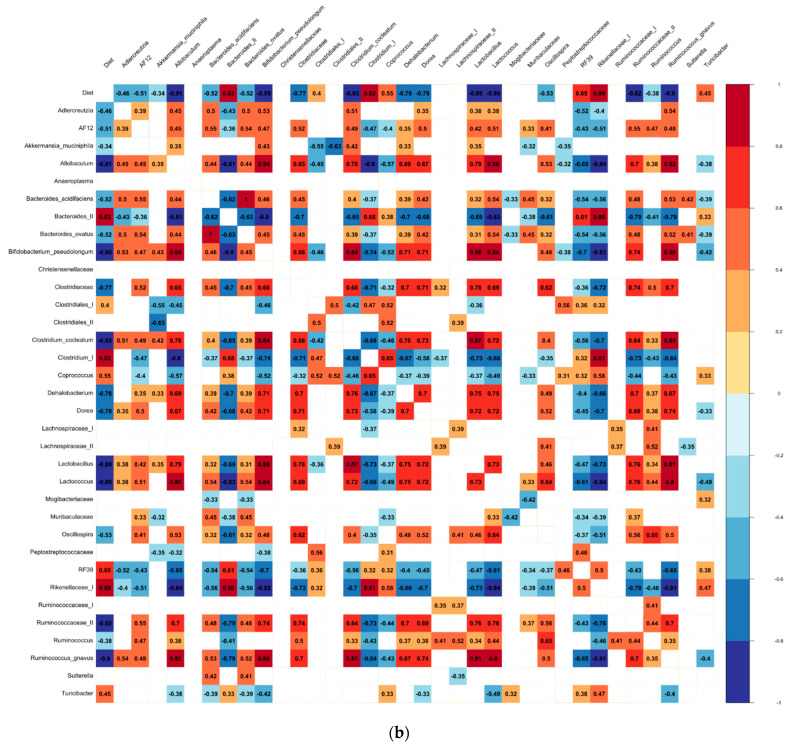
Spearman’s rank-order correlation analysis of the relative abundances of the cecal bacteria identified in the (**a**) female and (**b**) male cohorts. Heatmap represents power of correlation: from the red indicating positive to the blue indicating negative correlation coefficient values. Only correlations with the *p*-value < 0.05 were included in the correlogram.

Finally, the identified bacteria of the bean-induced ecosystem were confirmed in Spearman’s rank-order correlation (Figure 7). Interestingly, the same bacteria identified in the LEfSe runs correlated positively with their respective eco-groups and the bean dose. Muribaculaceae, RF39, *Sutterella*, Rikenellaceae (I), and *Bacteroides (II)* positively correlated with the bean dose, whereas *Lactococcus*, *Lactobacillus (I)*, Peptostreptococcaceae, *Allobaculum*, and *B. pseudolongum* displayed a strong negative association in the female cohort. In the male cohort, the most positively correlated bacteria with the bean dose were Rikenellaceae (I), *Bacteroides (II)*, *Clostridium (I)*, and RF39, whereas *B. pseudolongum*, *Allobaculum*, *R. gnavus*, *Lactococcus*, *Lactobacillus (I)*, *C. cocleatum*, and Ruminococcaceae (II) were the most negatively correlated with the bean dose. 

### 3.4. Predicted Functional Differences of the Microbial Communities

**Figure 8:**
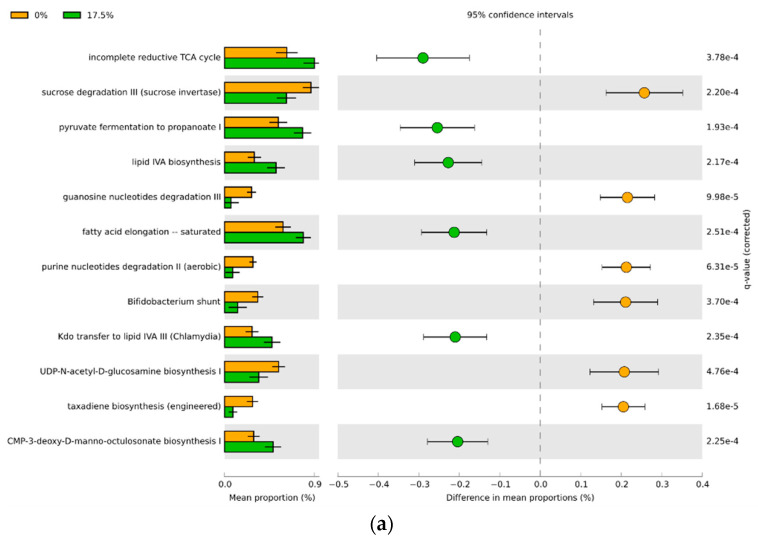

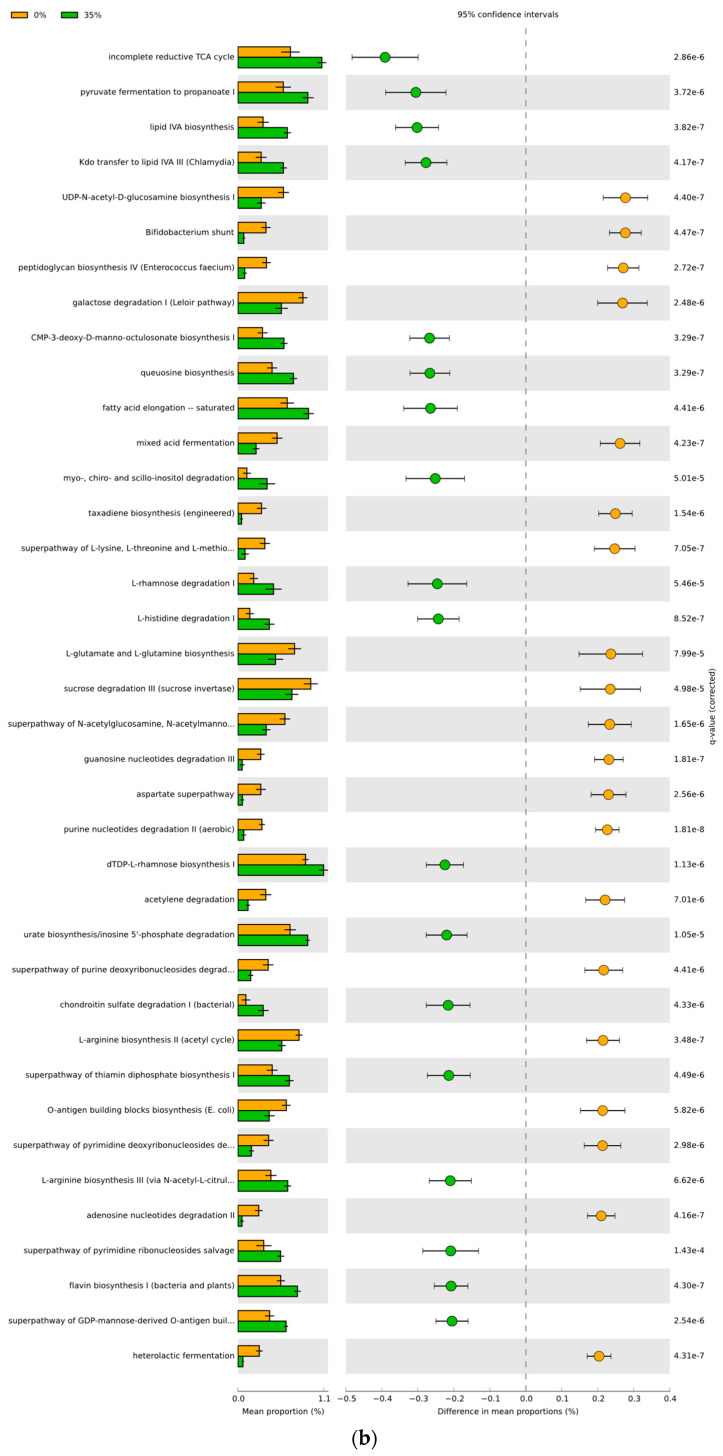

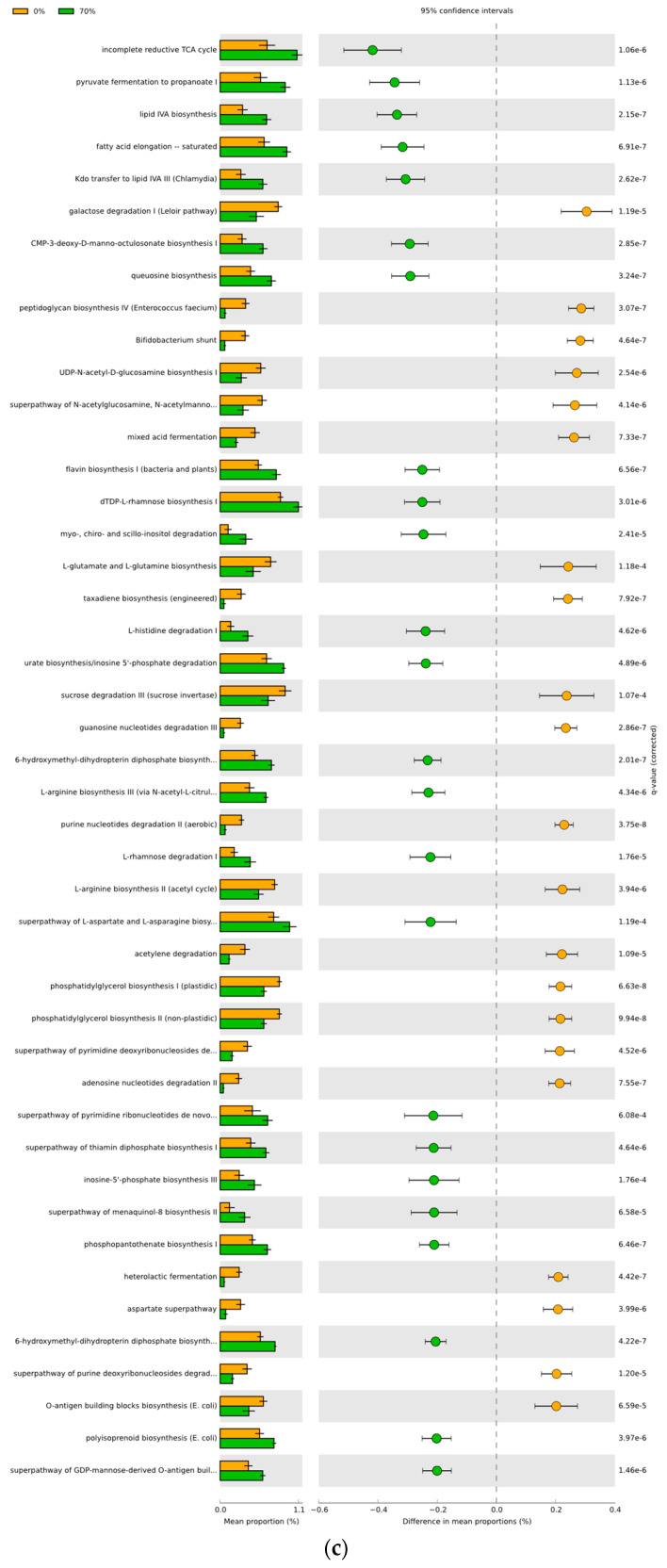
PICRUSt2 results indicating differential predicted functional pathways with 20% of the difference in the mean proportions between the bean-free and the bean-containing diet groups in the female cohort: bean-free diet (0%) is indicated in beige, while bean-based diet samples (**a**–17.5%; **b**–35%, and **c**–70%)—in green. Extended error bar plot indicating the mean proportion of pathways assigned to each group, difference between them, and corrected *p*-value (*q*-value) of each.

**Figure 9:**
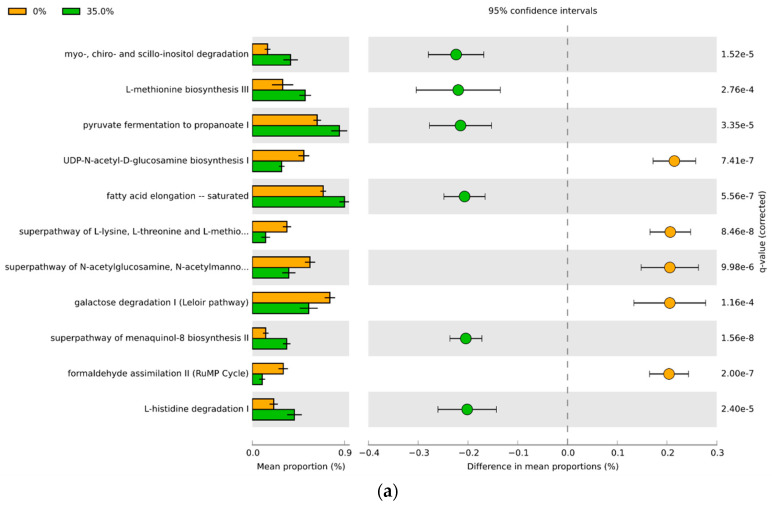

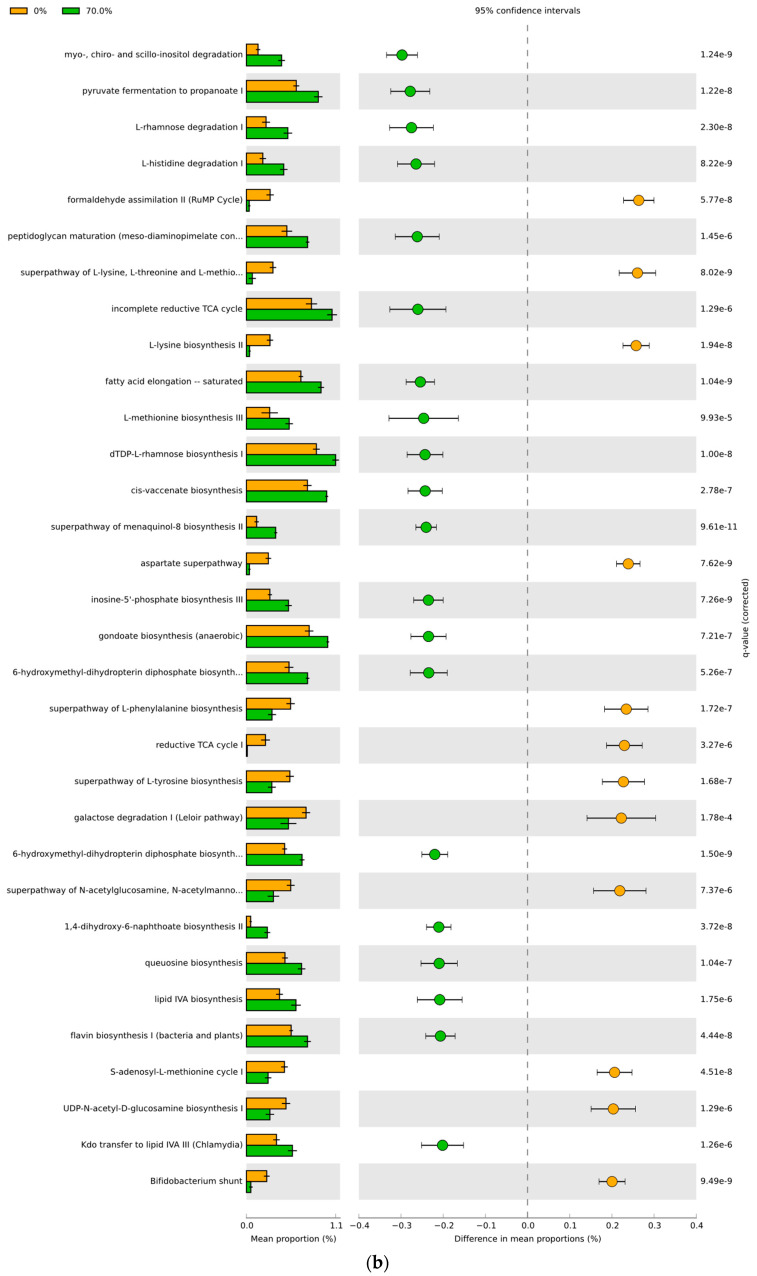
PICRUSt2 results indicating differential predicted functional pathways with 20% of the difference in the mean proportions between the bean-free and the bean-containing diet groups in the male cohort: bean-free diet (0%) is indicated in beige, while bean-based diet samples (**a**–35% and **b**–70%)—in green. Extended error bar plot indicating the mean proportion of pathways assigned to each group, difference between them, and corrected *p*-value (*q*-value) of each.

PICRUSt2 assessment of the predicted metagenomic functions overall showed that females displayed significantly more differentially represented pathways between the bean-free and bean-based diets compared to their male counterparts (Figure 8, Figure 9, Figures S8 and S9). Increasing doses of the dietary bean increased the number of differences in the bacterial metabolic pathways from 152 to 198 compared with the control. Out of these, 12–45 pathways had an effect size of over 20% difference in proportions. The male cecal microbiota responded more modestly upon the introduction of bean: from 95 to 160 differential pathways. There were 11 to 32 pathways with over 20% difference in proportions in the male cohort. Interestingly, males showed no predicted metabolic pathway differences between 0% and 17.5% diet groups, with over 20% difference in proportions. Overall, increasing bean dose in the diet predictably increased the amount of differential microbial functional pathways. 

## 4. Discussion

The diets used in this study were formulated to maintain equivalent macronutrient content—i.e., the percent (weight/weight) of protein, carbohydrate, and fat across the four diet groups. What varied were the qualitative characteristics of the dietary carbohydrate and protein components and the content of bean-associated bioactive compounds. The bean-free diet provided macronutrients from highly refined sources, whereas the common bean diets contained their dose-respective fraction derived from the cooked bean powder. In addition, these diets containing the whole food cooked beans provided a high variety of dietary phytochemicals, such as polyphenols among others, exerting additional effects. Diet formulations were adjusted according to the proximate analysis of the nutritional composition of the cooked bean [53,54,55,56]. Considering that common bean is generally consumed as a whole food, even when used as a recipe ingredient, the diet formulations investigated mimicked the manner in which human populations consume common bean [56]. 

### 4.1. Bean Effects on the Overall Diversity of the Cecal Microbiota

The assessment of microbial diversity under bean consumption indicated that increasing bean dose reduced the variety of bacterial species within samples and that the reduction was more robust in females than in male mice. The decrease in species richness was not expected in the bean fed groups given that a complex mixture of carbohydrate in common bean replaced refined carbohydrate sources. Thus, this observation may reflect either the presence of bacteriostatic factors in the common bean powder and/or that the bacterial taxa induced by feeding bean suppressed the growth of a broad range of co-occurring taxa. The observed differences in sex-associated responses were also unexpected and merit further investigation.

PCoA of three distance matrices (Jaccard, weighted/unweighted UniFrac) showed gradual and statistically significant distancing of the bean-containing samples with each increasing dose away from the bean-free control group. Each diet group was also significantly different between males and females. Samples of the 17.5% bean group localized in a transitioning ellipse between bean-free and bean-based groups of higher doses. Consistent with our findings, recently reported data in humans indicate sex differences in the ratio of total human-associated bacteria, dominated by the colonic microbiota, to the total cell count of referenced humans: 1.3:1 in males and 2.2:1 in females [57]. In mice, sex-dependent as well as sex-by-diet-dependent changes in the gut microbiota composition have been reported and attributed predominantly to hormonal effects, especially that of testosterone [58,59]. 

### 4.2. Biomarkers of the Bean-Induced Ecosystem

Consumption of common bean induced an increase in *Bacteroides (II), Clostridium (I),* Rikenellaceae (I), and RF39, as well as a reduction in counts of *Adlercreutzia*, *Allobaculum*, *B. pseudolongum*, *C. cocleatum*, Clostridiaceae (II), *Dehalobacterium*, *Dorea*, *Lactococcus*, *Oscillospira*, and *R. gnavus*, *Ruminococcus*, Ruminococceae (II) (Figures S6 and S7). These effects, which were dose dependent and observed in both sexes, parallel observations from studies in humans and are also consistent with other studies in mice. Obese humans were reported to exhibit lower abundances of Rikenellaceae [60,61,62], and so did mice [63,64,65]. In mice fed isocaloric yet different types of fiber-based diets, the Rikenellaceae were enhanced under dietary cellulose, inulin, and pectin [66], i.e., either insoluble or soluble types of dietary fiber that are also included in the dietary formulations studied here. Unclassified order RF39 was also detected by LEfSe as enhanced by the bean diets. RF39 was deemed as one of the major gut lineages of the human gut that showed the potential to produce acetate and hydrogen but lacked the capacity for other numerous highly conserved metabolic pathways [67,68]. 

Some dose-dependent biomarkers of the bean-induced changes in the cecal microbiota composition differed between male and female cohorts. The bean-induced changes in the female cohort were distinguished from those in the male one by representatives of Muribaculaceae and *Sutterella*, which were enhanced upon a 35% and higher bean doses (Figure S6a), as well as by the suppressed *A. muciniphila*, unclassified Clostridiales (I), Clostridiaceae (II), *Clostridium (II)*, *Enterococcus*, Mogibacteriaceae, Peptostreptococcaceae, Ruminococcaceae (I), *Streptococcus (I)*, and *Turicibacter* (Figure S7a). When LEfSe was set to determine the sex-dependent biomarkers of each diet group, only Muribaculaceae were also statistically more abundant in females versus males, whereas abundances of *A. muciniphila*, *Sutterella*, Mogibacteriaceae, Peptostreptococcaceae, and Ruminococcaceae (I) were significantly higher in the male cohort (Figure 6). Muribaculaceae are one of the dominant gut bacteria engaged in degradation of dietary carbohydrates and whose abundance is decreased by high-fat diet conditions [30,69] and obesity resistance [70], yet increased upon bean consumption [71,72,73]. Research on *Sutterella* reported in the literature is equivocal in the context of dietary responses and health impacts. Several studies showed increased *Sutterella* in mice fed low-fat diets [74,75], while others showed decreased abundance under similar conditions [75,76]. Interestingly, in our previous report, Muribaculaceae and *Sutterella* were also a member of the pulse-enhanced eco-group, but in the male mice, including those fed 35% of bean [23]. The unclassified *Clostridium* mapped to two different species (marked I and II). In our experiments, bean dose-dependently enhanced *Clostridium (I)* but suppressed the abundance of *Clostridium (II)* (Figures S6a and S7a). Additionally, unclassified Clostridiales (I) were suppressed by bean consumption in the female mice’s cecal microbiota starting at the 35% dose (Figure S7a), but was then enhanced in the male cohort upon a 70% dose of bean (Figure S6b). Clostridia, in general, are associated with various functions as commensal bacteria, ranging from their potential as probiotics to their role in a number of human diseases [77,78]. 

On the other hand, the male cohort contrasted to the female cohort with the bean-enhanced *Coprococcus* and *Clostridiales (I)* (Figure S6b) as well as the suppressed *Parabacteroides, Lactobacillus (I)*, *AF12*, *B. acidifaciens*, and *B. ovatus* (Figure S7b) in their bean-induced microbial ecosystem. *B. ovatus* counts were statistically higher in male compared with female mice, yet *Lactobacillus (I)* were more abundant in the female cohort. *Bacteroides spp.* are obligate anaerobes, found solely in the gastrointestinal tract of mammals, showing great plasticity and adaptability in their commensalism and mutualism [79]. They have received mixed associations with obesity [62,80,81,82] but were shown to utilize and respond positively to dietary fiber, such as inulin and xyloglucan, by *B. acidifaciens* and *B. ovatus*, in particular [83,84,85]. *B. acidifaciens* has also been shown to decrease upon feeding of the insoluble high-fiber diet [86]. 

### 4.3. Potential Impact of the Bean Macronutrients

#### 4.3.1. Protein

Diets with an increased intake of protein, such as the Paleolithic and ketogenic diets, have increased in popularity in recent years [87]. However, several studies have shown quantitatively different effects on the microbiota with consumption of animal versus plant proteins [88]. This study was controlled for total protein in the diet and therefore was designed to evaluate how the source of dietary protein affects the gut microbiota. The quality (biological value) of protein from plant sources is well-known to be lower than that from animal sources [89]. For pulses in general, and for common bean in particular, sulfur-containing amino acids and tryptophan are the first and second most limiting amino acids [90], and they were provided in the diet formulation. In addition, the digestibility of bean protein has been reported to be lower than many protein sources, presumably because of the presence of trypsin and chymotrypsin inhibitors. However, these antinutrient factors are eliminated when bean is soaked and cooked before eating [91,92], which is how the bean was prepared in this study. PICRUSt2 analysis of the predicted microbial function herein demonstrated that bean consumption was not dominated by pathways associated with protein fermentation, aside from significant L-histidine degradation (I), nor branched short-chain fatty acid production. This finding is not surprising given that we have previously reported that mice and rats have the same growth efficiency when pair-fed the highest bean dose studied herein, suggesting similar utilization of dietary protein [25,26]. Additionally, dietary fiber is known to reduce fermentation of protein in the gut of monogastric species [93,94]. Thus, these findings fail to indicate that consuming up to 70% of dietary protein from common bean, when properly processed, would result in negative metabolic effects associated with excessive protein fermentation in the gut [94,95]. However, bean-containing diets were also distinguished by L-arginine biosynthesis (III) via *N*-acetyl-L-citrulline, whereas the bean-free group upregulated L-arginine biosynthesis (II) via acetyl cycle. The bean-free group was also distinguished with significant increases in superpathways of L-lysine, L-threonine, L-methionine biosynthesis (I), L-phenylalanine biosynthesis, L-tyrosine biosynthesis, aspartate superpathway, L-lysine biosynthesis (II), and S-adenosyl-L-methionine cycle (I), and this was more evident in male than female mice. This is consistent with the fact that common bean is rich in the levels of lysine, tyrosine, and phenylalanine [53,96,97]. Interestingly, despite the inclusion of methionine in their diet formulations, bean-based groups were still marked with L-methionine biosynthesis (III) pathway upregulation. 

#### 4.3.2. Carbohydrate

Early reports underestimated the total amount of dietary fiber in common bean and other pulses. However, the adoption of an internationally accepted consensus definition of dietary fiber and of a method for its measurement have revealed that the amount of dietary fiber and protein are similar on the basis of the percent of dry matter [98]. Moreover, we have reported how pulse consumption in both the U.S. and Canada has the potential to close the dietary fiber gap [99]. What this study did was to afford an opportunity to understand how a change in carbohydrate quality from refined to increasing amounts of whole food-derived carbohydrate, that is particularly rich in its fiber content, impacted commonly studied characteristics of the gut microbiota. 

Common bean is a rich source of carbohydrates (60–70% of dry weight) [97]. Among these, the most prevalent prebiotic components are resistant starch, sugar alcohols (such as mannitol, sorbitol, and xylitol), raffinose oligosaccharides (stachyose, raffinose, verbascose), fructose oligosaccharides (kestose and nystose), and long-chain oligosaccharides (inulin) [97,100,101]. The functional profile of the bean-induced ecosystem was, in part, characterized by an incomplete reductive TCA cycle and pyruvate fermentation to propanoate I. Propanoate, or propionate, is one of the main short-chain fatty acids produced by the gut microbial metabolism and is associated with hepatic gluconeogenesis and glucose metabolism, adipocyte leptin signaling and reduced fat accumulation, reduced inflammation via HDAC inhibition, and overall health-promoting properties [102,103,104,105].

A particularly interesting observation was that incorporation of bean into the diet reduces the abundance of Actinomycetota, predominantly represented by *Bifidobacterium pseudolongum.* A characteristic feature of these bacteria is the *Bifidobacterium* shunt that ferments glucose to lactate—also downregulated compared with the bean-free control group. *B. pseudolongum* showed a similar pattern of change together with other lactic acid-producing bacteria, such as *Lactobacillus*, *Lactococcus*, and *Streptococcus.* These bacteria, in particular, are associated with the galactose metabolism [106,107], and we report a concomitant downregulation of the Leloir pathway of galactose degradation (I), homo- and heterolactic fermentation, and others. Interestingly, these bacteria can also metabolize partially hydrolyzed proteins, including casein [64,107,108,109], producing bioactive peptides. In diets with increasing bean doses, casein proportionally declines, which might have contributed to reductions in the abundance of these bacteria. However, metabolizing dietary protein by the gut microbiota generally has a negative connotation [94,110,111,112], and *Bifidobacterium*, *Lactobacillus*, *Lactococcus*, and *Streptococcus* can produce toxic compounds upon metabolizing proteins [94,110,113], including biogenic amines, ammonia, phenol/indoles, etc. Considering our results, bean may protect the mice from fermentation of proteins due to its high content of dietary fiber [94].

#### 4.3.3. Bioactives

Pulse bioactives, some of which have been referred to as antinutrients, may be involved in mediating effects on the gut microbiota [114]. For example, phenols are often poorly absorbed and thus reach the large intestine, where they interact directly with the gut microorganisms. Common bean has been reported to contain various phenolic acids (chlorogenic acid, gallic acid, p-hydroxybenzoic acid, caffeic acid, protocatechuic acid, p-coumaric acid, rosmarinic acid, ferulic acid, sinapic acid, and ellagic acid) and flavonoids (epicatechin, catechin, gallocatechin gallate, epigallocatechin gallate, quercetin, hesperidin, and rutin), as well as various polyphenols [53]. These compounds may simultaneously induce duplibiotic effects, i.e., antimicrobial and prebiotic activities [115]. This could explain the relandscaping of gut bacterial composition that we observed upon increasing the dietary bean dose: fewer taxa were enhanced than suppressed. Metabolomic and metametabolomic studies are needed to further elucidate the role that these low molecular compounds and their microbiota-generated derivatives play in accounting for bean-induced effects on the gut microbial composition. 

## 5. Summary and Implications

This is a deep, comprehensive analysis of the sex- as well as dose-dependent differences in the cecal bacteria composition of mice upon consumption of cooked whole-food bean in the diet when overall macronutrient content was held constant across the treatment groups. Identified eco-groups provide insight into the generalized effects of bean consumption as a whole food on the gut microbiota.

The impact of biological sex on the composition of the bacterial ecosystem that persisted despite an increase in the amount of bean consumed has not been, to our knowledge, reported previously. Our data suggest that females will be more responsive to bean consumption than males at any dose consumed. Given that there are many noted weaknesses in the clinical and population studies of the consumption of common bean and other pulse crops on disease risk, the recognition of potential biological sex differences to pulse consumption in both study design and data analysis has the potential to improve the sensitivity and statistical power of studies in humans. Investigation of the origins of the biological sex differences may also provide insights about common bean-gut microbiota-host interactions important to developing precision approaches to chronic disease prevention and control.

This investigation also has implications for the dose of common bean and other pulses that is required to improve health and well-being as well as mitigate disease risks. Current recommendations appear to be based less on scientific evidence and more on what it is presumed that consumers will eat [6]. The science reported herein indicates the potential value of levels of consumption typical of high pulse consumers identified in both NHANES and its Canadian equivalent [19,116]. Moreover, even higher levels of consumption may hold benefits if mediation is via the gut microbiota. Clearly, more work on target levels of consumption is required, and this must include well-designed and controlled clinical investigations.

Using mice as a preclinical animal model to study the effects of diet on gut microbial composition has both strengths and limitations [31,32,117,118]. This work differs from other reports in that animals were received from the same vendor and housed in the same animal room so that reported differences that can arise from vendor, shipment, and animal husbandry practices were minimized. Consequently, the likelihood that all mice had similar species richness within the gut at the beginning of feeding of the test diet formulations was maximized. We acknowledge that the composition of these eco-groups may change among studies but, in our judgment, this type of assessment redirects focus towards collective changes in the microbial groups rather than the solitary differences in each. Clearly, the next step in advancing understanding of the diet-microbiota-host paradigm requires extension of the research approach beyond the question of defining “who’s home” to determining what the microbial ecosystem is doing and where within the gut the microbiota are exerting effects via yet-to-be-identified chemical mediators that impact the host.

## Figures and Tables

**Table 1:** Experimental diets formulations.

Ingredient	0% Bean	17.5% Bean ^1^	35% Bean ^1^	70% Bean ^1^
	(g/100 g)	(g/100 g)	(g/100 g)	(g/100 g)
Solka-Floc	7.5	4.0	2.0	1.0
White Kidney Bean ^2^	0.0	10.2	20.4	40.8
Corn Starch	15.9	15.2	12.1	2.0
Casein (≥85% protein)	23.5	19.4	15.3	7.0
Cerelose (Dextrose)	7.2	7.2	7.2	7.2
Sucrose	25.0	23.0	22.0	21.0
Vitamin mix ^3^	1.1	1.1	1.1	1.1
DL-Methionine	0.3	0.3	0.3	0.3
L-Tryptophan (Sigma T0254-25G)	0.00	0.02	0.03	0.06
Choline bitartrate (41% choline)	0.2	0.2	0.2	0.2
Mineral mix ^4^	3.8	3.8	3.8	3.8
Corn Oil	11.3	11.3	11.3	11.3
Anhydrous Butter Fat	4.2	4.2	4.2	4.2
***Total (g)***	***100.0***	***100.0***	***100.0***	***100.0***

^1^ Experimental diet modified from the original (bean-free control) diet formulations; all formulations were formulated to be 58.6% carbohydrate, 15.5% fat and 20% protein, *w*/*w*. ^2^ For the bean-fed groups, the whole bean was cooked and processed with the leachate, freeze-dried, and homogenized into a fine powder; total dietary fiber concentration of white kidney bean was 23.5 g/100g; ^3^ Dyets Inc: #310025 AIN-93G vitamin mix; ^4^ Dyets Inc: #210025 AIN-93G mineral mix.

**Table 2:** Relative abundances of phyla per each sex cohort and diet group.

Phyla		Female				Male			
	Bean dose	*0%*	*17.5%*	*35%*	*70%*	*0%*	*17.5%*	*35%*	*70%*
Actinomycetota		2.72	0.61	0.16 ^1,^***	0.09 ^1,^***^; 2,^*	2.48	1.05	0.52 ^1,^***	0.09 ^1,^***^; 2,^***
Bacteroidota		37.30	56.65	66.47 ^1,^***	74.12 ^1,^***^; 2^,**	39.35	49.52	56.32 ^1,^**	63.47 ^1,^***^; 2,^**
Bacillota		43.03	23.85 ^1,^**	20.77 ^1,^***	17.23 ^1,^***	36.29	26.15 ^1,^*	23.55 ^1,^**	22.88 ^1,^**
Pseudomonadota		1.52	1.77	2.21	3.14 ^1,^**^; 2,^**	3.83	5.24	3.99	3.81
Mycoplasmatota		0.02	0.17	0.25 ^1,^*	0.47 ^1,^***^; 2,^*	0.04	0.03	0.11 ^1,^**^; 2,^**	0.13 ^1,^**^; 2,^**
Verrucomicrobiota		15.40	16.95	10.13	4.94 ^1,^*^; 2,^***^; 3,^*	18.02	18.01	15.50	9.62 ^2,^*

^1^ Significantly different from the 0% group; ^2^ Significantly different from the 17.5% group; ^3^ Significantly different from the 35% group; Statistically significant phyla were determined by the Kruskal-Wallis testing of the relative abundances with * *p*-value < 0.05; ** p-value < 0.01; *** *p*-value < 0.001. *p*-values adjusted for multiple comparisons with the Benjamini-Hochberg method.

## Data Availability

Available upon request.

## Supplementary Materials

The following supporting information can be downloaded at: https://www.mdpi.com/article/10.3390/foods11081153/s1, Figure S1: Observed features of α-diversity of the female and male cohorts; Figure S2: Faith’s phylogenetic α-diversity of the female and male cohort; Figure S3: Sex-pairwise comparison of α diversity; Figure S4: Jaccard indices were used to explain β-diversity across the diet groups; Figure S5: Phylogenetic Unweighted UniFrac distances were used to explain β-diversity across diet groups; Figure S6: Heatmap representing hierarchical clustering analysis results at the feature level; Figure S7: Bacterial sex-dependent biomarkers of the female and male cohorts in each diet group according to the LEfSe at the feature level; Figure S8: PICRUSt2 results indicating differential predicted functional pathways between the bean-free and the bean-containing diet groups in the female cohort; Figure S9: PICRUSt2 results indicating differential predicted functional pathways between the bean-free and the bean-containing diet groups in the male cohort.

## References

[1] World Health Organization Noncommunicable Diseases. https://www.who.int/news-room/fact-sheets/detail/noncommunicable-diseases.

[2] Neuhouser M.L. (2019). The importance of healthy dietary patterns in chronic disease prevention. Nutr. Res..

[3] Johnson M. (2019). Diet and nutrition: Implications to cardiometabolic health. J. Cardiol. Cardiovasc. Sci..

[4] Ioannidis J.P.A. (2018). The Challenge of Reforming Nutritional Epidemiologic Research. JAMA J. Am. Med. Assoc..

[5] Mozaffarian D., Rosenberg I., Uauy R. (2018). History of modern nutrition science—Implications for current research, dietary guidelines, and food policy. BMJ.

[6] Cámara M., Giner R.M., González-Fandos E., López-García E., Mañes J., Portillo M.P., Rafecas M., Domínguez L., Martínez J.A. (2021). Food-Based Dietary Guidelines around the World: A Comparative Analysis to Update AESAN Scientific Committee Dietary Recommendations. Nutrients.

[7] U.S. Department of Agriculture and U.S. Department of Health and Human Services. Dietary Guidelines for Americans, 2020–2025. https://www.dietaryguidelines.gov/.

[8] Ha K., Sakaki J.R., Chun O.K. (2021). Nutrient Adequacy Is Associated with Reduced Mortality in US Adults. J. Nutr..

[9] Bailey R.L., Ard J.D., Davis T.A., Naimi T.S., Schneeman B.O., Stang J.S., Dewey K.G., Donovan S.M., Novotny R., Snetselaar L.G. (2021). A Proposed Framework for Identifying Nutrients and Food Components of Public Health Relevance in the Dietary Guidelines for Americans. J. Nutr..

[10] McBurney M.I., Blumberg J.B., Costello R.B., Eggersdorfer M., Erdman J.W., Harris W.S., Johnson E.J., Hazels Mitmesser S., Post R.C., Rai D. (2021). Beyond Nutrient Deficiency—Opportunities to Improve Nutritional Status and Promote Health Modernizing DRIs and Supplementation Recommendations. Nutrients.

[11] Barber T.M., Kabisch S., Pfeiffer A.F.H., Weickert M.O. (2020). The Health Benefits of Dietary Fibre. Nutrients.

[12] Lockyer S., Spiro A., Stanner S. (2016). Dietary fibre and the prevention of chronic disease – Should health professionals be doing more to raise awareness?. Nutr. Bull..

[13] Makki K., Deehan E.C., Walter J., Bäckhed F. (2018). The Impact of Dietary Fiber on Gut Microbiota in Host Health and Disease. Cell Host Microbe.

[14] Tanes C., Bittinger K., Gao Y., Friedman E.S., Nessel L., Paladhi U.R., Chau L., Panfen E., Fischbach M.A., Braun J. (2021). Role of dietary fiber in the recovery of the human gut microbiome and its metabolome. Cell Host Microbe.

[15] So D., Whelan K., Rossi M., Morrison M., Holtmann G., Kelly J.T., Shanahan E.R., Staudacher H.M., Campbell K.L. (2018). Dietary fiber intervention on gut microbiota composition in healthy adults: A systematic review and meta-analysis. Am. J. Clin. Nutr..

[16] Didinger C., Thompson H. (2020). Motivating Pulse-Centric Eating Patterns to Benefit Human and Environmental Well-Being. Nutrients.

[17] Didinger C., Thompson H.J. (2021). Defining Nutritional and Functional Niches of Legumes: A Call for Clarity to Distinguish a Future Role for Pulses in the Dietary Guidelines for Americans. Nutrients.

[18] Thompson H.J. (2021). The Dietary Guidelines for Americans (2020–2025): Pulses, Dietary Fiber, and Chronic Disease Risk—A Call for Clarity and Action. Nutrients.

[19] Mitchell D.C., Marinangeli C.P.F., Pigat S., Bompola F., Campbell J., Pan Y., Curran J.M., Cai D.J., Jaconis S.Y., Rumney J. (2021). Pulse Intake Improves Nutrient Density among US Adult Consumers. Nutrients.

[20] Viguiliouk E., Mejia S.B., Kendall C.W., Sievenpiper J.L. (2017). Can pulses play a role in improving cardiometabolic health? Evidence from systematic reviews and meta-analyses. Ann. N. Y. Acad. Sci..

[21] Ferreira H., Vasconcelos M., Gil A.M., Pinto E. (2021). Benefits of pulse consumption on metabolism and health: A systematic review of randomized controlled trials. Crit. Rev. Food Sci. Nutr..

[22] Kazemi M., Buddemeyer S., Fassett C.M., Gans W.M., Johnston K.M., Lungu E., Savelle R.L., Tolani P.N., Dahl W.J., Dahl W.J. (2019). Pulses and Prevention and Management of Chronic Disease. Health Benefits of Pulses.

[23] Lutsiv T., Weir T.L., McGinley J.N., Neil E.S., Wei Y., Thompson H.J. (2021). Compositional Changes of the High-Fat Diet-Induced Gut Microbiota upon Consumption of Common Pulses. Nutrients.

[24] McGinley J.N., Fitzgerald V.K., Neil E.S., Omerigic H.M., Heuberger A.L., Weir T.L., McGee R., Vandemark G., Thompson H.J. (2020). Pulse Crop Effects on Gut Microbial Populations, Intestinal Function, and Adiposity in a Mouse Model of Diet-Induced Obesity. Nutrients.

[25] Thompson H.J., McGinley J.N., Neil E.S., Brick M.A. (2017). Beneficial Effects of Common Bean on Adiposity and Lipid Metabolism. Nutrients.

[26] Neil E.S., McGinley J.N., Fitzgerald V.K., Lauck C.A., Tabke J.A., Streeter-McDonald M.R., Yao L., Broeckling C.D., Weir T.L., Foster M.T. (2019). White Kidney Bean (*Phaseolus vulgaris* L.) Consumption Reduces Fat Accumulation in a Polygenic Mouse Model of Obesity. Nutrients.

[27] Jiang W., Zhu Z., McGinley J.N., El Bayoumy K., Manni A., Thompson H.J. (2012). Identification of a Molecular Signature Underlying Inhibition of Mammary Carcinoma Growth by Dietary N-3 Fatty Acids. Cancer Res..

[28] Mensack M.M., McGinley J.N., Thompson H.J. (2012). Metabolomic analysis of the effects of edible dry beans (*Phaseolus vulgaris* L.) on tissue lipid metabolism and carcinogenesis in rats. Br. J. Nutr..

[29] Oren A., Garrity G.M. (2021). Valid publication of the names of forty-two phyla of prokaryotes. Int. J. Syst. Evol. Microbiol..

[30] Lagkouvardos I., Lesker T.R., Hitch T.C.A., Gálvez E.J.C., Smit N., Neuhaus K., Wang J., Baines J.F., Abt B., Stecher B. (2019). Sequence and cultivation study of Muribaculaceae reveals novel species, host preference, and functional potential of this yet undescribed family. Microbiome.

[31] Zhang C., Franklin C.L., Ericsson A.C. (2021). Consideration of Gut Microbiome in Murine Models of Diseases. Microorganisms.

[32] Hugenholtz F., De Vos W.M. (2018). Mouse models for human intestinal microbiota research: A critical evaluation. Cell. Mol. Life Sci..

[33] Chu D.-T., Malinowska E., Jura M., Kozak L.P. (2017). C57BL/6J mice as a polygenic developmental model of diet-induced obesity. Physiol. Rep..

[34] Jang H. (2017). High-Fat Diets for Diet-Induced Obesity (DIO) Models. https://www.google.com.hk/url?sa=t&rct=j&q=&esrc=s&source=web&cd=&cad=rja&uact=8&ved=2ahUKEwi3j8DO0JX3AhXhDaYKHXs0Br8QFnoECAYQAQ&url=https%3A%2F%2Fresearchdiets.com%2Fsystem%2Frefinery%2Fresources%2FW1siZiIsIjIwMTkvMTEvMjIvMjh0emRubzVjOF9SRElfb2Jlc2l0eV93ZWIzLnBkZiJdXQ%2FRDI_obesity_web3.pdf&usg=AOvVaw117IvZ5aJSByvrn0bdS_d3.

[35] Lee D.M., Battson M.L., Jarrell D.K., Hou S., Ecton K.E., Weir T.L., Gentile C.L. (2018). SGLT2 inhibition via dapagliflozin improves generalized vascular dysfunction and alters the gut microbiota in type 2 diabetic mice. Cardiovasc. Diabetol..

[36] Bolyen E., Rideout J.R., Dillon M.R., Bokulich N.A., Abnet C.C., Al-Ghalith G.A., Alexander H., Alm E.J., Arumugam M., Asnicar F. (2019). Reproducible, interactive, scalable and extensible microbiome data science using QIIME 2. Nat. Biotechnol..

[37] Callahan B.J., Mcmurdie P.J., Rosen M.J., Han A.W., Johnson A.J.A., Holmes S.P. (2016). DADA2: High-resolution sample inference from Illumina amplicon data. Nat. Methods.

[38] Bokulich N.A., Kaehler B.D., Rideout J.R., Dillon M., Bolyen E., Knight R., Huttley G.A., Gregory Caporaso J. (2018). Optimizing taxonomic classification of marker-gene amplicon sequences with QIIME 2’s q2-feature-classifier plugin. Microbiome.

[39] McDonald D., Price M.N., Goodrich J., Nawrocki E.P., DeSantis T.Z., Probst A., Andersen G.L., Knight R., Hugenholtz P. (2012). An improved Greengenes taxonomy with explicit ranks for ecological and evolutionary analyses of bacteria and archaea. ISME J..

[40] Price M.N., Dehal P.S., Arkin A.P. (2010). FastTree 2—Approximately Maximum-Likelihood Trees for Large Alignments. PLoS ONE.

[41] Katoh K., Standley D.M. (2013). MAFFT Multiple Sequence Alignment Software Version 7: Improvements in Performance and Usability. Mol. Biol. Evol..

[42] Dhariwal A., Chong J., Habib S., King I.L., Agellon L.B., Xia J. (2017). MicrobiomeAnalyst: A web-based tool for comprehensive statistical, visual and meta-analysis of microbiome data. Nucleic Acids Res..

[43] Chong J., Liu P., Zhou G., Xia J. (2020). Using MicrobiomeAnalyst for comprehensive statistical, functional, and meta-analysis of microbiome data. Nat. Protoc..

[44] Foster Z.S.L., Sharpton T.J., Grünwald N.J. (2017). Metacoder: An R package for visualization and manipulation of community taxonomic diversity data. PLoS Comput. Biol..

[45] Segata N., Izard J., Waldron L., Gevers D., Miropolsky L., Garrett W.S., Huttenhower C. (2011). Metagenomic biomarker discovery and explanation. Genome Biol..

[46] Douglas G.M., Maffei V.J., Zaneveld J.R., Yurgel S.N., Brown J.R., Taylor C.M., Huttenhower C., Langille M.G.I. (2020). PICRUSt2 for prediction of metagenome functions. Nat. Biotechnol..

[47] Barbera P., Kozlov A.M., Czech L., Morel B., Darriba D., Flouri T., Stamatakis A. (2019). EPA-ng: Massively Parallel Evolutionary Placement of Genetic Sequences. Syst. Biol..

[48] Czech L., Barbera P., Stamatakis A. (2020). Genesis and Gappa: Processing, analyzing and visualizing phylogenetic (placement) data. Bioinformatics.

[49] Louca S., Doebeli M. (2017). Efficient comparative phylogenetics on large trees. Bioinformatics.

[50] Ye Y., Doak T.G. (2009). A Parsimony Approach to Biological Pathway Reconstruction/Inference for Genomes and Metagenomes. PLoS Comput. Biol..

[51] Caspi R., Billington R., Fulcher C.A., Keseler I.M., Kothari A., Krummenacker M., Latendresse M., Midford P.E., Ong Q., Ong W.K. (2018). The MetaCyc database of metabolic pathways and enzymes. Nucleic Acids Res..

[52] Parks D.H., Tyson G.W., Hugenholtz P., Beiko R.G. (2014). STAMP: Statistical analysis of taxonomic and functional profiles. Bioinformatics.

[53] Ganesan K., Xu B. (2017). Polyphenol-Rich Dry Common Beans (*Phaseolus vulgaris* L.) and Their Health Benefits. Int. J. Mol. Sci..

[54] de Almeida Costa G.E., da Silva Queiroz-Monici K., Reis S.M.P.M., de Oliveira A.C. (2006). Chemical composition, dietary fibre and resistant starch contents of raw and cooked pea, common bean, chickpea and lentil legumes. Food Chem..

[55] Kleintop A.E., Echeverria D., Brick L.A., Thompson H.J., Brick M.A. (2013). Adaptation of the AOAC 2011.25 Integrated Total Dietary Fiber Assay To Determine the Dietary Fiber and Oligosaccharide Content of Dry Edible Beans. J. Agric. Food Chem..

[56] Didinger C., Foster M.T., Bunning M., Thompson H.J. (2021). Nutrition and Human Health Benefits of Dry Beans and Other Pulses. Dry Beans and Pulses.

[57] Sender R., Fuchs S., Milo R. (2016). Revised Estimates for the Number of Human and Bacteria Cells in the Body. PLoS Biol..

[58] Org E., Mehrabian M., Parks B.W., Shipkova P., Liu X., Drake T.A., Lusis A.J. (2016). Sex differences and hormonal effects on gut microbiota composition in mice. Gut Microbes.

[59] Kim Y.S., Unno T., Kim B.-Y., Park M.-S. (2020). Sex Differences in Gut Microbiota. World J. Men’s Health.

[60] Koo S.H., Chu C.W., Khoo J.J.C., Cheong M., Soon G.H., Ho E.X.P., Law N.M., De Sessions P.F., Fock K.M., Ang T.L. (2019). A pilot study to examine the association between human gut microbiota and the host’s central obesity. JGH Open.

[61] Del Chierico F., Abbatini F., Russo A., Quagliariello A., Reddel S., Capoccia D., Caccamo R., Ginanni Corradini S., Nobili V., De Peppo F. (2018). Gut Microbiota Markers in Obese Adolescent and Adult Patients: Age-Dependent Differential Patterns. Front. Microbiol..

[62] Crovesy L., Masterson D., Rosado E.L. (2020). Profile of the gut microbiota of adults with obesity: A systematic review. Eur. J. Clin. Nutr..

[63] Ye J., Zhao Y., Chen X., Zhou H., Yang Y., Zhang X., Huang Y., Zhang N., Lui E.M., Xiao M. (2021). Pu-erh tea ameliorates obesity and modulates gut microbiota in high fat diet fed mice. Food Res. Int..

[64] Li A., Wang N., Li N., Li B., Yan F., Song Y., Hou J., Huo G. (2021). Modulation effect of chenpi extract on gut microbiota in high-fat diet-induced obese C57BL/6 mice. J. Food Biochem..

[65] Liu Y., Gao Y., Ma F., Sun M., Mu G., Tuo Y. (2020). The ameliorative effect of Lactobacillus plantarum Y44 oral administration on inflammation and lipid metabolism in obese mice fed with a high fat diet. Food Funct..

[66] Murga-Garrido S.M., Hong Q., Cross T.-W.L., Hutchison E.R., Han J., Thomas S.P., Vivas E.I., Denu J., Ceschin D.G., Tang Z.-Z. (2021). Gut microbiome variation modulates the effects of dietary fiber on host metabolism. Microbiome.

[67] Wang Y., Huang J.-M., Zhou Y.-L., Almeida A., Finn R.D., Danchin A., He L.-S. (2020). Phylogenomics of expanding uncultured environmental Tenericutes provides insights into their pathogenicity and evolutionary relationship with Bacilli. BMC Genom..

[68] Nayfach S., Shi Z.J., Seshadri R., Pollard K.S., Kyrpides N.C. (2019). New insights from uncultivated genomes of the global human gut microbiome. Nature.

[69] Ding Y., Song Z., Li H., Chang L., Pan T., Gu X., He X., Fan Z. (2019). Honokiol Ameliorates High-Fat-Diet-Induced Obesity of Different Sexes of Mice by Modulating the Composition of the Gut Microbiota. Front. Immunol..

[70] Cao W., Chin Y., Chen X., Mi Y., Xue C., Wang Y., Tang Q. (2019). The role of gut microbiota in the resistance to obesity in mice fed a high fat diet. Int. J. Food Sci. Nutr..

[71] Monk J.M., Wu W., Lepp D., Pauls K.P., Robinson L.E., Power K.A. (2021). Navy Bean Supplementation in Established High-Fat Diet-Induced Obesity Attenuates the Severity of the Obese Inflammatory Phenotype. Nutrients.

[72] Tan Y., Tam C., Meng S., Zhang Y., Alves P., Yokoyama W. (2021). Cooked Black Turtle Beans Ameliorate Insulin Resistance and Restore Gut Microbiota in C57BL/6J Mice on High-Fat Diets. Foods.

[73] Mariana J.C.G., da Silva J.S., Assis A., de Mejia E.G., Mantovani H.C., Martino H.S.D. (2021). Common Bean (*Phaseolus vulgaris* L.) Flour Can Improve the Gut Microbiota Composition and Function in Mice Fed a High-Fat Diet. Curr. Dev. Nutr..

[74] Jang L.-G., Choi G., Kim S.-W., Kim B.-Y., Lee S., Park H. (2019). The combination of sport and sport-specific diet is associated with characteristics of gut microbiota: An observational study. J. Int. Soc. Sports Nutr..

[75] Wang L., Ren B., Hui Y., Chu C., Zhao Z., Zhang Y., Zhao B., Shi R., Ren J., Dai X. (2020). Methionine Restriction Regulates Cognitive Function in High-Fat Diet-Fed Mice: Roles of Diurnal Rhythms of SCFAs Producing- and Inflammation-Related Microbes. Mol. Nutr. Food Res..

[76] Hwang N., Eom T., Gupta S.K., Jeong S.-Y., Jeong D.-Y., Kim Y.S., Lee J.-H., Sadowsky M.J., Unno T. (2017). Genes and Gut Bacteria Involved in Luminal Butyrate Reduction Caused by Diet and Loperamide. Genes.

[77] Wei G., Ye Y., Yan X., Chao X., Yang F., Wang M., Zhang W., Yuan C., Zeng Q. (2020). Effect of banana pulp dietary fibers on metabolic syndrome and gut microbiota diversity in high-fat diet mice. J. Food Biochem..

[78] Mallozzi M., Viswanathan V., Vedantam G. (2010). Spore-forming Bacilli and Clostridia in human disease. Future Microbiol..

[79] Wexler A.G., Goodman A.L. (2017). An insider’s perspective: Bacteroides as a window into the microbiome. Nat. Microbiol..

[80] Yoshida N., Yamashita T., Osone T., Hosooka T., Shinohara M., Kitahama S., Sasaki K., Sasaki D., Yoneshiro T., Suzuki T. (2021). *Bacteroides* spp. promotes branched-chain amino acid catabolism in brown fat and inhibits obesity. iScience.

[81] Castaner O., Goday A., Park Y.-M., Lee S.-H., Magkos F., Shiow S.-A.T.E., Schröder H. (2018). The Gut Microbiome Profile in Obesity: A Systematic Review. Int. J. Endocrinol..

[82] Tseng C.H., Wu C.Y. (2019). The gut microbiome in obesity. J. Formos. Med. Assoc..

[83] Chijiiwa R., Hosokawa M., Kogawa M., Nishikawa Y., Ide K., Sakanashi C., Takahashi K., Takeyama H. (2020). Single-cell genomics of uncultured bacteria reveals dietary fiber responders in the mouse gut microbiota. Microbiome.

[84] Holscher H.D. (2017). Dietary fiber and prebiotics and the gastrointestinal microbiota. Gut Microbes.

[85] Hemsworth G.R., Thompson A.J., Stepper J., Sobala Ł.F., Coyle T., Larsbrink J., Spadiut O., Goddard-Borger E.D., Stubbs K.A., Brumer H. (2016). Structural dissection of a complex *Bacteroides ovatus* gene locus conferring xyloglucan metabolism in the human gut. Open Biol..

[86] Nakajima A., Sasaki T., Itoh K., Kitahara T., Takema Y., Hiramatsu K., Ishikawa D., Shibuya T., Kobayashi O., Osada T. (2020). A Soluble Fiber Diet Increases Bacteroides fragilis Group Abundance and Immunoglobulin A Production in the Gut. Appl. Environ. Microbiol..

[87] Sukkar S.G., Muscaritoli M. (2021). A Clinical Perspective of Low Carbohydrate Ketogenic Diets: A Narrative Review. Front. Nutr..

[88] Tomova A., Bukovsky I., Rembert E., Yonas W., Alwarith J., Barnard N.D., Kahleova H. (2019). The Effects of Vegetarian and Vegan Diets on Gut Microbiota. Front. Nutr..

[89] Lonnie M., Hooker E., Brunstrom J.M., Corfe B.M., Green M.A., Watson A.W., Williams E.A., Stevenson E.J., Penson S., Johnstone A.M. (2018). Protein for Life: Review of Optimal Protein Intake, Sustainable Dietary Sources and the Effect on Appetite in Ageing Adults. Nutrients.

[90] Derbyshire E., Delange J., Tiwari B.K., Gowen A., McKenna B. (2021). Chapter 2—The nutritional value of whole pulses and pulse fractions. Pulse Foods.

[91] Maqbool N., Sofi S.A., Makroo H.A., Mir S.A., Majid D., Dar B. (2021). Cooking methods affect eating quality, bio-functional components, antinutritional compounds and sensory attributes of selected vegetables. Ital. J. Food Sci..

[92] Wainaina I., Wafula E., Sila D., Kyomugasho C., Grauwet T., Van Loey A., Hendrickx M. (2021). Thermal treatment of common beans (*Phaseolus vulgaris* L.): Factors determining cooking time and its consequences for sensory and nutritional quality. Compr. Rev. Food Sci. Food Saf..

[93] Jha R., Fouhse J.M., Tiwari U.P., Li L., Willing B.P. (2019). Dietary Fiber and Intestinal Health of Monogastric Animals. Front. Vet. Sci..

[94] Diether N.E., Willing B.P. (2019). Microbial Fermentation of Dietary Protein: An Important Factor in Diet–Microbe–Host Interaction. Microorganisms.

[95] Yao C.K., Muir J.G., Gibson P.R. (2016). Review article: Insights into colonic protein fermentation, its modulation and potential health implications. Aliment. Pharmacol. Ther..

[96] Margier M., Georgé S., Hafnaoui N., Remond D., Nowicki M., Du Chaffaut L., Amiot M.-J., Reboul E. (2018). Nutritional Composition and Bioactive Content of Legumes: Characterization of Pulses Frequently Consumed in France and Effect of the Cooking Method. Nutrients.

[97] Hall C., Hillen C., Garden Robinson J. (2017). Composition, Nutritional Value, and Health Benefits of Pulses. Cereal Chem..

[98] Division Naval Air Facility Atsugi (2017). FAO/INFOODS Global Database for Pulses on Dry Matter Basis.

[99] Thompson H.J., Brick M.A. (2016). Perspective: Closing the Dietary Fiber Gap: An Ancient Solution for a 21st Century Problem. Adv. Nutr. Int. Rev. J..

[100] Siva N., Thavarajah P., Thavarajah D. (2020). Prebiotic carbohydrate concentrations of common bean and chickpea change during cooking, cooling, and reheating. J. Food Sci..

[101] Siva N., Thavarajah P., Kumar S., Thavarajah D. (2019). Variability in Prebiotic Carbohydrates in Different Market Classes of Chickpea, Common Bean, and Lentil Collected From the American Local Market. Front. Nutr..

[102] Koh A., De Vadder F., Kovatcheva-Datchary P., Bäckhed F. (2016). From Dietary Fiber to Host Physiology: Short-Chain Fatty Acids as Key Bacterial Metabolites. Cell.

[103] Xiang S., Ye K., Li M., Ying J., Wang H., Han J., Shi L., Xiao J., Shen Y., Feng X. (2021). Xylitol enhances synthesis of propionate in the colon via cross-feeding of gut microbiota. Microbiome.

[104] Zaibi M.S., Stocker C.J., O’Dowd J., Davies A., Bellahcene M., Cawthorne M.A., Brown A.J., Smith D.M., Arch J.R. (2010). Roles of GPR41 and GPR43 in leptin secretory responses of murine adipocytes to short chain fatty acids. FEBS Lett..

[105] Kumar J., Rani K., Datt C. (2020). Molecular link between dietary fibre, gut microbiota and health. Mol. Biol. Rep..

[106] Iskandar C.F., Cailliez-Grimal C., Borges F., Revol-Junelles A.-M. (2019). Review of lactose and galactose metabolism in Lactic Acid Bacteria dedicated to expert genomic annotation. Trends Food Sci. Technol..

[107] Pessione E. (2012). Lactic acid bacteria contribution to gut microbiota complexity: Lights and shadows. Front. Cell. Infect. Microbiol..

[108] Zhao F., Song S., Ma Y., Xu X., Zhou G., Li C. (2019). A Short-Term Feeding of Dietary Casein Increases Abundance of Lactococcus lactis and Upregulates Gene Expression Involving Obesity Prevention in Cecum of Young Rats Compared with Dietary Chicken Protein. Front. Microbiol..

[109] Centanni M., Lawley B., Butts C.A., Roy N.C., Lee J., Kelly W.J., Tannock G.W., McBain A.J. (2018). Bifidobacterium pseudolongum in the Ceca of Rats Fed Hi-Maize Starch Has Characteristics of a Keystone Species in Bifidobacterial Blooms. Appl. Environ. Microbiol..

[110] Oliphant K., Allen-Vercoe E. (2019). Macronutrient metabolism by the human gut microbiome: Major fermentation by-products and their impact on host health. Microbiome.

[111] Ma N., Tian Y., Wu Y., Ma X. (2017). Contributions of the Interaction Between Dietary Protein and Gut Microbiota to Intestinal Health. Curr. Protein Pept. Sci..

[112] Zhao J., Zhang X., Liu H., Brown M.A., Qiao S. (2018). Dietary Protein and Gut Microbiota Composition and Function. Curr. Protein Pept. Sci..

[113] Barbieri F., Montanari C., Gardini F., Tabanelli G. (2019). Biogenic Amine Production by Lactic Acid Bacteria: A Review. Foods.

[114] Giuberti G., Tava A., Mennella G., Pecetti L., Masoero F., Sparvoli F., Lo Fiego A., Campion B. (2019). Nutrients’ and Antinutrients’ Seed Content in Common Bean (*Phaseolus vulgaris* L.) Lines Carrying Mutations Affecting Seed Composition. Agronomy.

[115] Rodríguez-Daza M.C., Pulido-Mateos E.C., Lupien-Meilleur J., Guyonnet D., Desjardins Y., Roy D. (2021). Polyphenol-Mediated Gut Microbiota Modulation: Toward Prebiotics and Further. Front. Nutr..

[116] Mudryj A.N., Yu N., Hartman T.J., Mitchell D.C., Lawrence F.R., Aukema H.M. (2012). Pulse consumption in Canadian adults influences nutrient intakes. Br. J. Nutr..

[117] Kim D., Hofstaedter C.E., Zhao C., Mattei L., Tanes C., Clarke E., Lauder A., Sherrill-Mix S., Chehoud C., Kelsen J. (2017). Optimizing methods and dodging pitfalls in microbiome research. Microbiome.

[118] Laukens D., Brinkman B.M., Raes J., De Vos M., Vandenabeele P. (2016). Heterogeneity of the gut microbiome in mice: Guidelines for optimizing experimental design. FEMS Microbiol. Rev..

