# Supplementary material for: Relandscaping the Gut Microbiota with a Whole Food: Dose–Response Effects to Common Bean
Source: Foods. 2022 Apr 15;11(8):1153. doi: 10.3390/foods11081153 (PMC9025344; doi:10.3390/foods11081153)
Supplement: Supplementary file 1 [file foods-11-01153-s001.zip › foods-1655675-supplementary.pdf]

Supplementary Materials

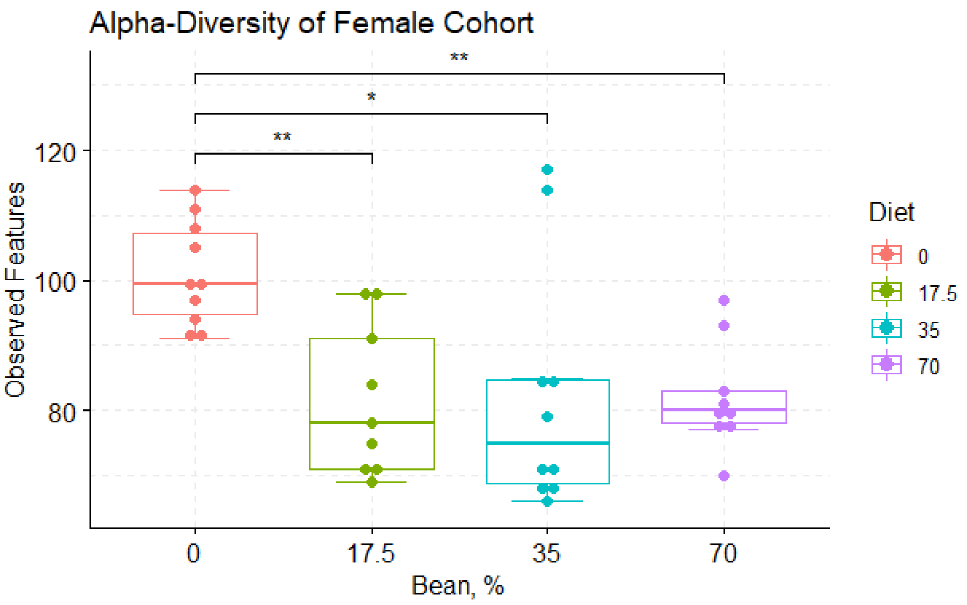

a

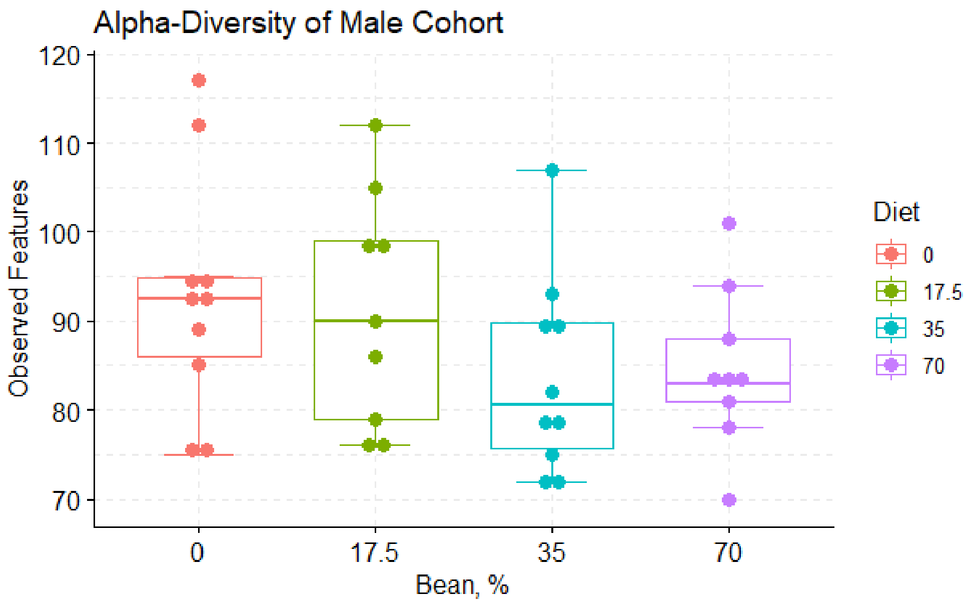

b

Figure S1. (a) Observed features of α-diversity of the female cohort. Statistically significant differences were determined by the Kruskal-Wallis test (p-value = 0.006) with * q-value < 0.05; ** q-value < 0.01; **(b)** Observed features of α-diversity of the male cohort. Statistically significant differences were tested with the Kruskal-Wallis method (p-value = 0.265).

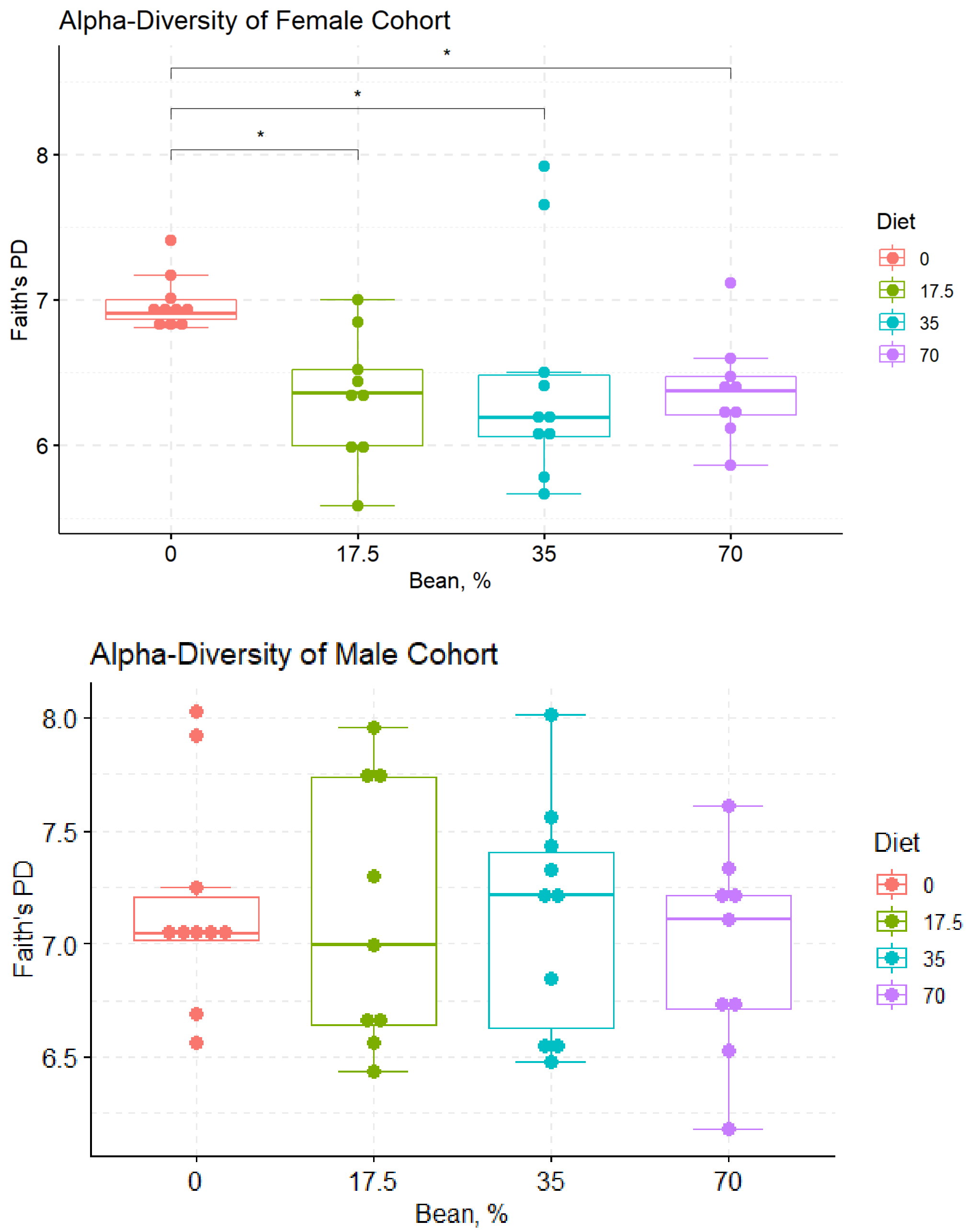

Figure S2. (a) Faith's phylogenetic α-diversity of the female cohort. Statistically significant differences were determined by the Kruskal-Wallis test (p-value = 0.008) with * q-value < 0.05; ** q-value < 0.01; **(b)** Faith's phylogenetic α-diversity of the male cohort. Statistically significant differences were tested with the Kruskal-Wallis method (p-value = 0.941).

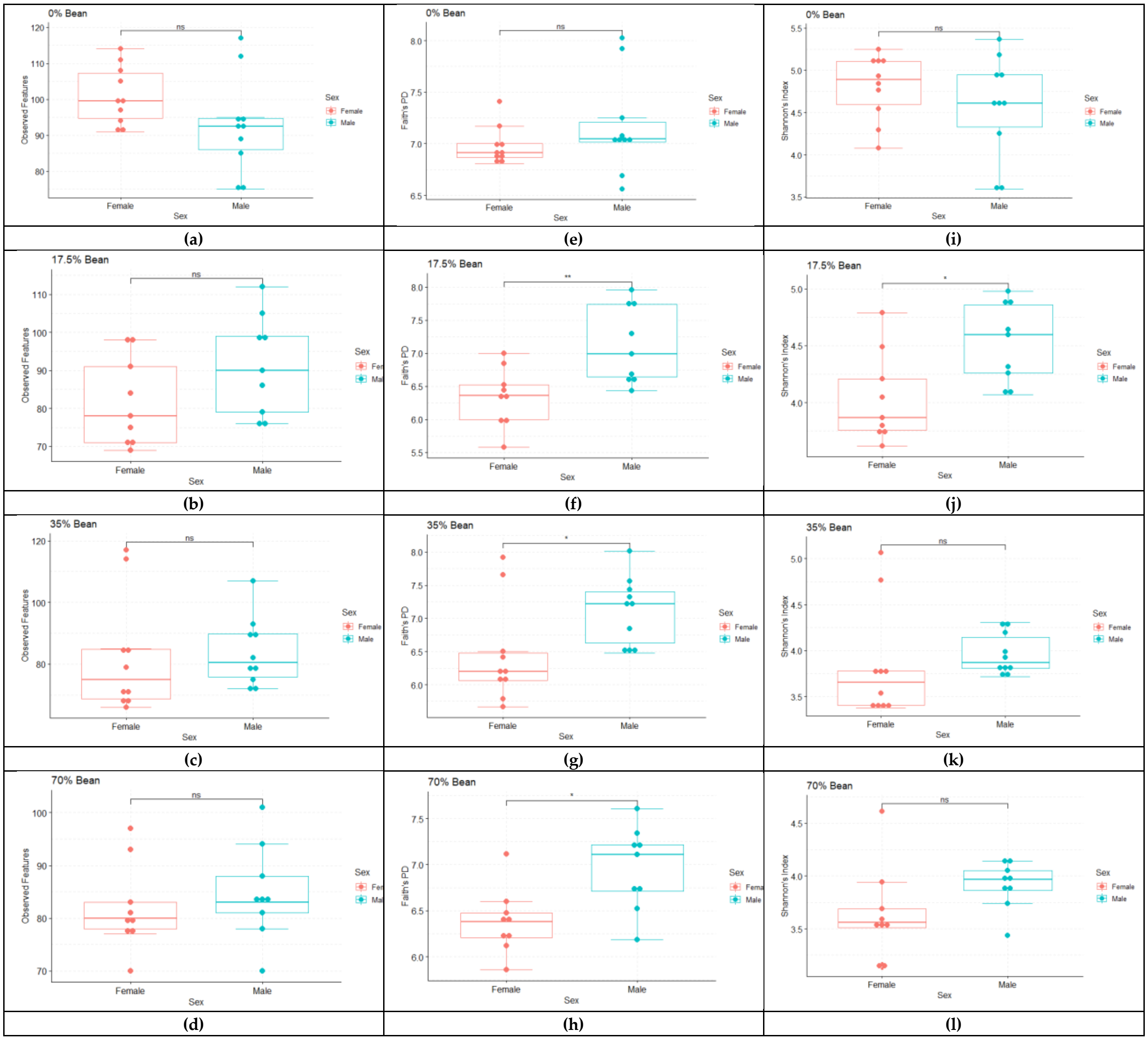

Figure S3. Sex-pairwise comparison of α-diversity. Statistically significant differences were determined by the Wilcoxon signed-rank tests with * p-value < 0.05; ** p-value < 0.01; **(a—d)** Observed α-diversity features; **(e—h)** Faith's phylogenetic α-diversity; **(i—l)** Shannon's indices of α diversity.

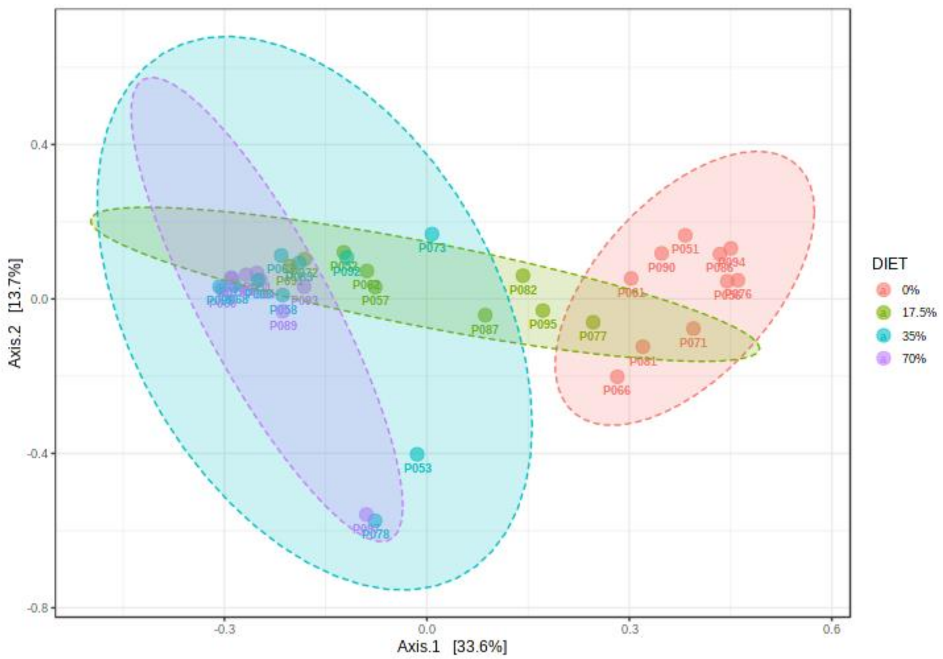

a

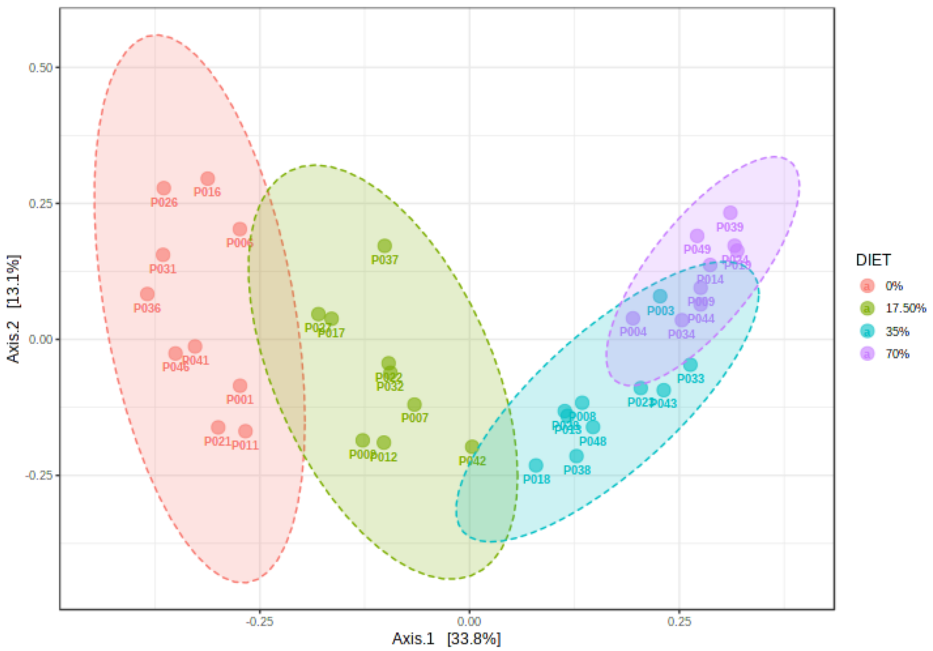

b

Figure S4. Jaccard indices were used to explain β-diversity across the diet groups. Differences between the samples were tested by the permutational multivariate analysis of variance (PERMANOVA): samples are colored and grouped by their corresponding diet group into ellipses representing 95% confidence intervals. **(a)** in the female cohort, pseudo-*F*-value = 7.251; *p*-value < 0.001. All diet groups statistically differed from one another; **(b)** in the male cohort, pseudo-*F*-value = 8.85; *p*-value < 0.001. All diet groups statistically differed from one another.

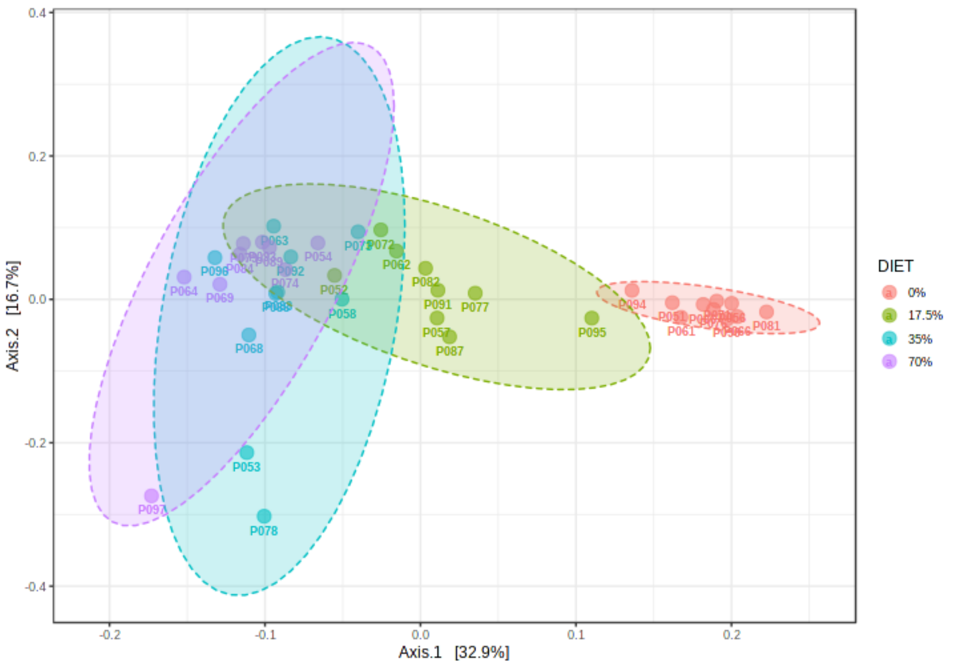

a

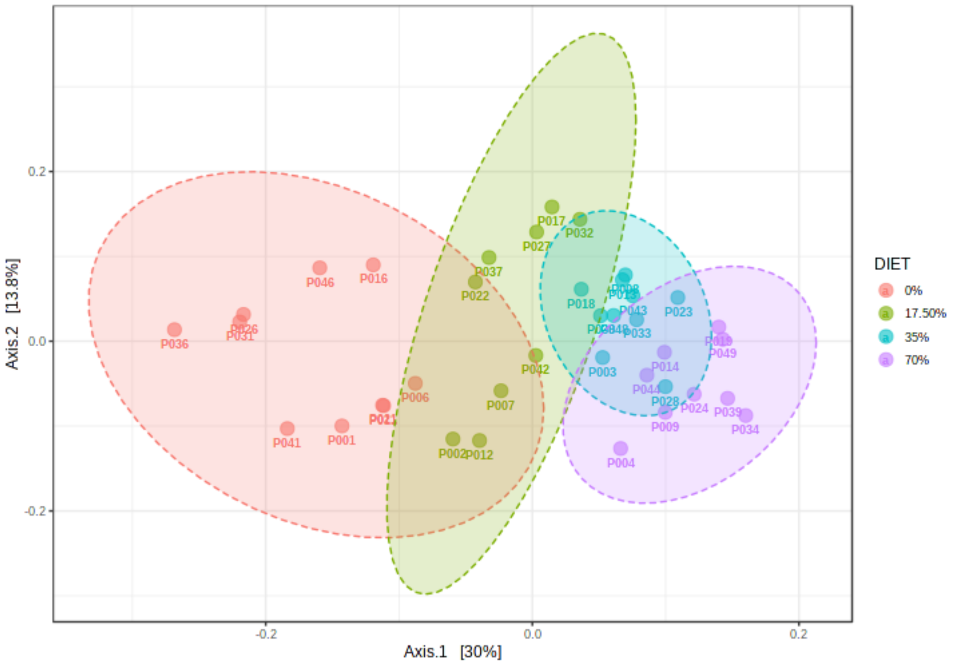

b

Figure S5. Phylogenetic Unweighted UniFrac distances were used to explain β-diversity across diet groups. Differences between the samples were tested by the permutational multivariate analysis of variance (PERMANOVA): samples are colored and grouped by their corresponding diet group into ellipses representing 95% confidence intervals. **(a)** in the female cohort, pseudo-*F*-value = 7.236; *p*-value < 0.001. All diet groups differed from one another, except for 35% vs 70% groups; **(b)** in the male cohort, pseudo-*F*-value = 7.192; *p*-value < 0.001. All diet groups differed from one another.

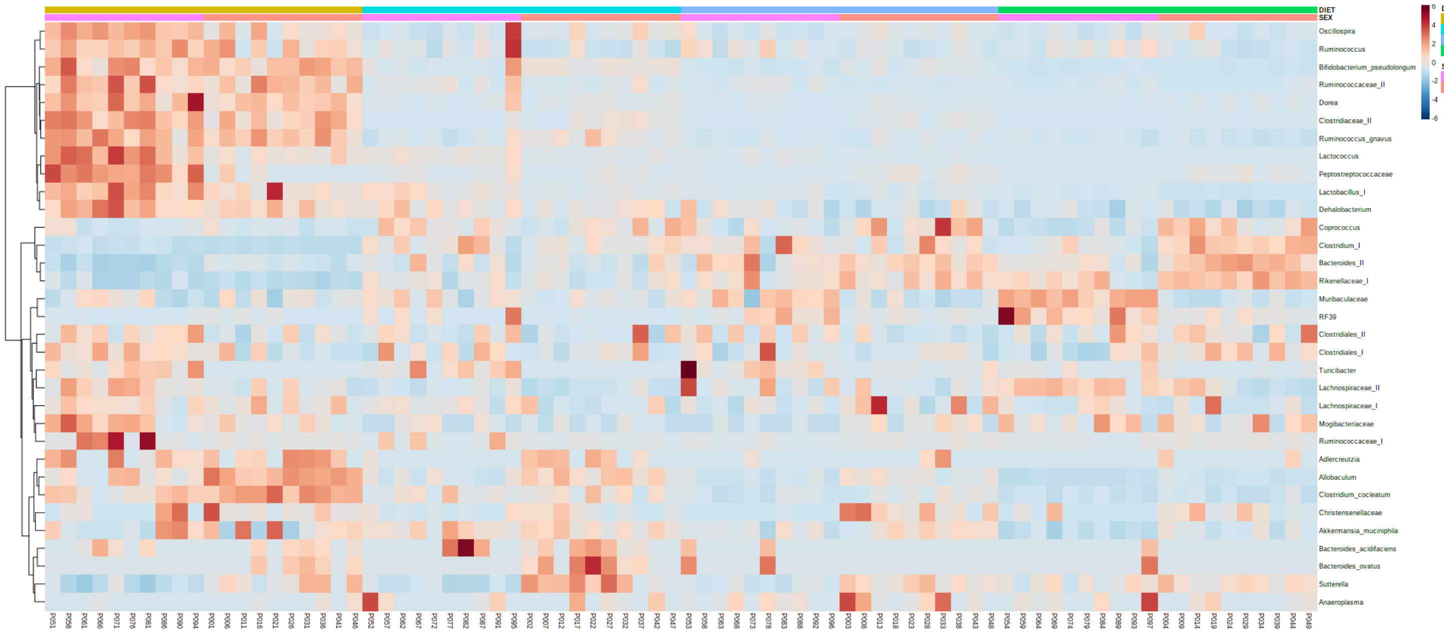

Figure S6. Heatmap representing hierarchical clustering analysis results at the feature level. Clusters were organized by the diet group factor using Ward's algorithm and Minkowski's distance measure.

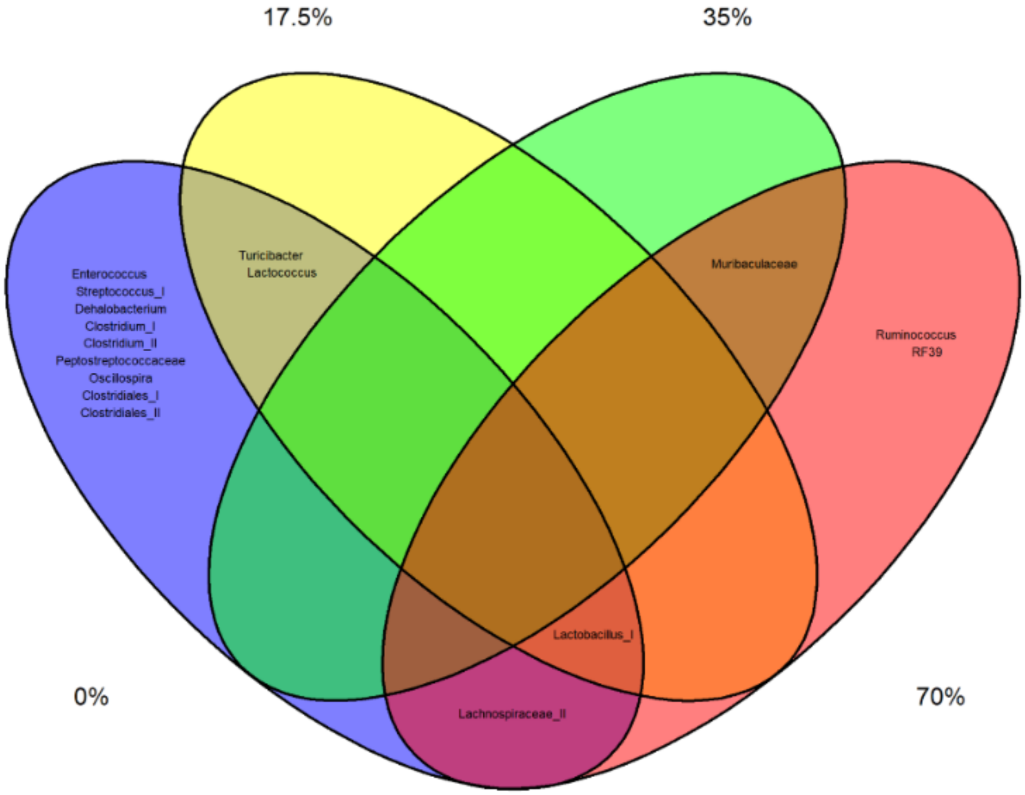

a

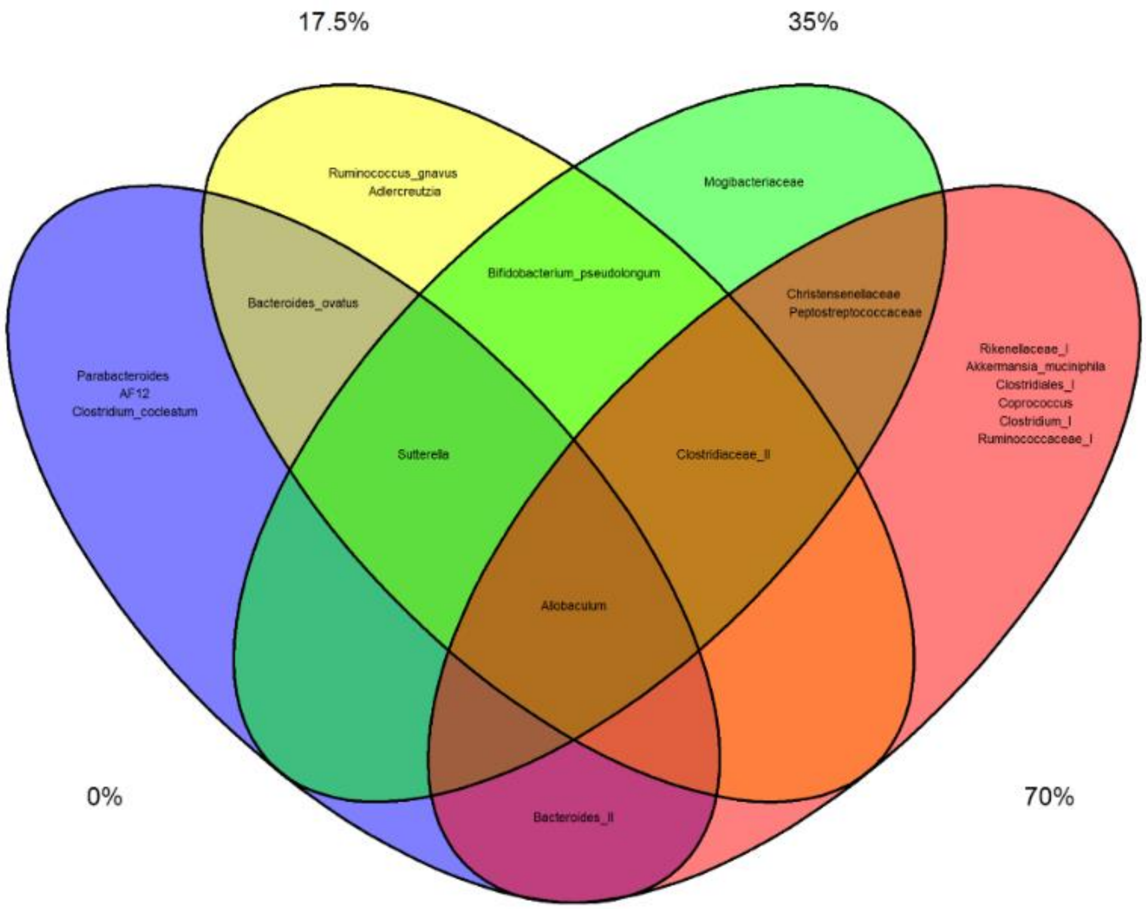

b

Figure S7. Bacterial sex-dependent biomarkers of the **(a)** female and **(b)** male cohorts in each diet group according to the pairwise linear discriminant analysis (LDA) of effect size (LEfSe) at the feature level. All represented bacteria were statistically significant (LDA score > |2.0|, FDR-adjusted *p*-value < 0.05).

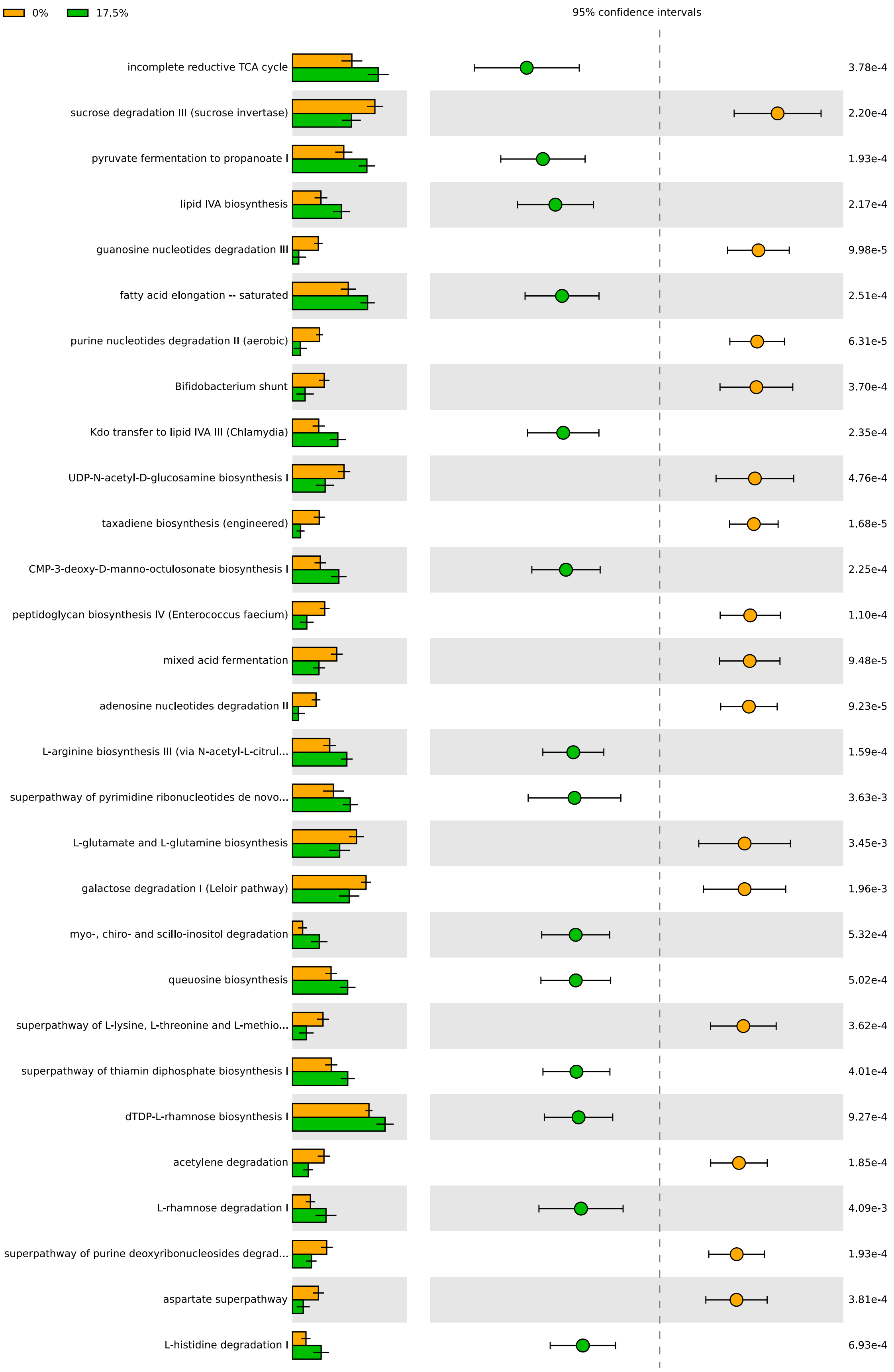

0%
17.5%
95% confidence intervals
incomplete reductive TCA cycle
3.78e-4
sucrose degradation III (sucrose invertase)
2.20e-4
pyruvate fermentation to propanoate I
1.93e-4
lipid IVA biosynthesis
2.17e-4
guanosine nucleotides degradation III
9.98e-5
fatty acid elongation -- saturated
2.51e-4
purine nucleotides degradation II (aerobic)
6.31e-5
Bifidobacterium shunt
3.70e-4
Kdo transfer to lipid IVA III (Chlamydia)
2.35e-4
UDP-N-acetyl-D-glucosamine biosynthesis I
4.76e-4
taxadiene biosynthesis (engineered)
1.68e-5
CMP-3-deoxy-D-manno-octulosonate biosynthesis I
2.25e-4
peptidoglycan biosynthesis IV (Enterococcus faecium)
1.10e-4
mixed acid fermentation
9.48e-5
adenosine nucleotides degradation II
9.23e-5
L-arginine biosynthesis III (via N-acetyl-L-citrul...
1.59e-4
superpathway of pyrimidine ribonucleotides de novo...
3.63e-3
L-glutamate and L-glutamine biosynthesis
3.45e-3
galactose degradation I (Leloir pathway)
1.96e-3
myo-, chiro- and scillo-inositol degradation
5.32e-4
queuosine biosynthesis
5.02e-4
superpathway of L-lysine, L-threonine and L-methio...
3.62e-4
superpathway of thiamin diphosphate biosynthesis I
4.01e-4
dTDP-L-rhamnose biosynthesis I
9.27e-4
acetylene degradation
1.85e-4
L-rhamnose degradation I
4.09e-3
superpathway of purine deoxyribonucleosides degrad...
1.93e-4
aspartate superpathway
3.81e-4
L-histidine degradation I
6.93e-4

a.

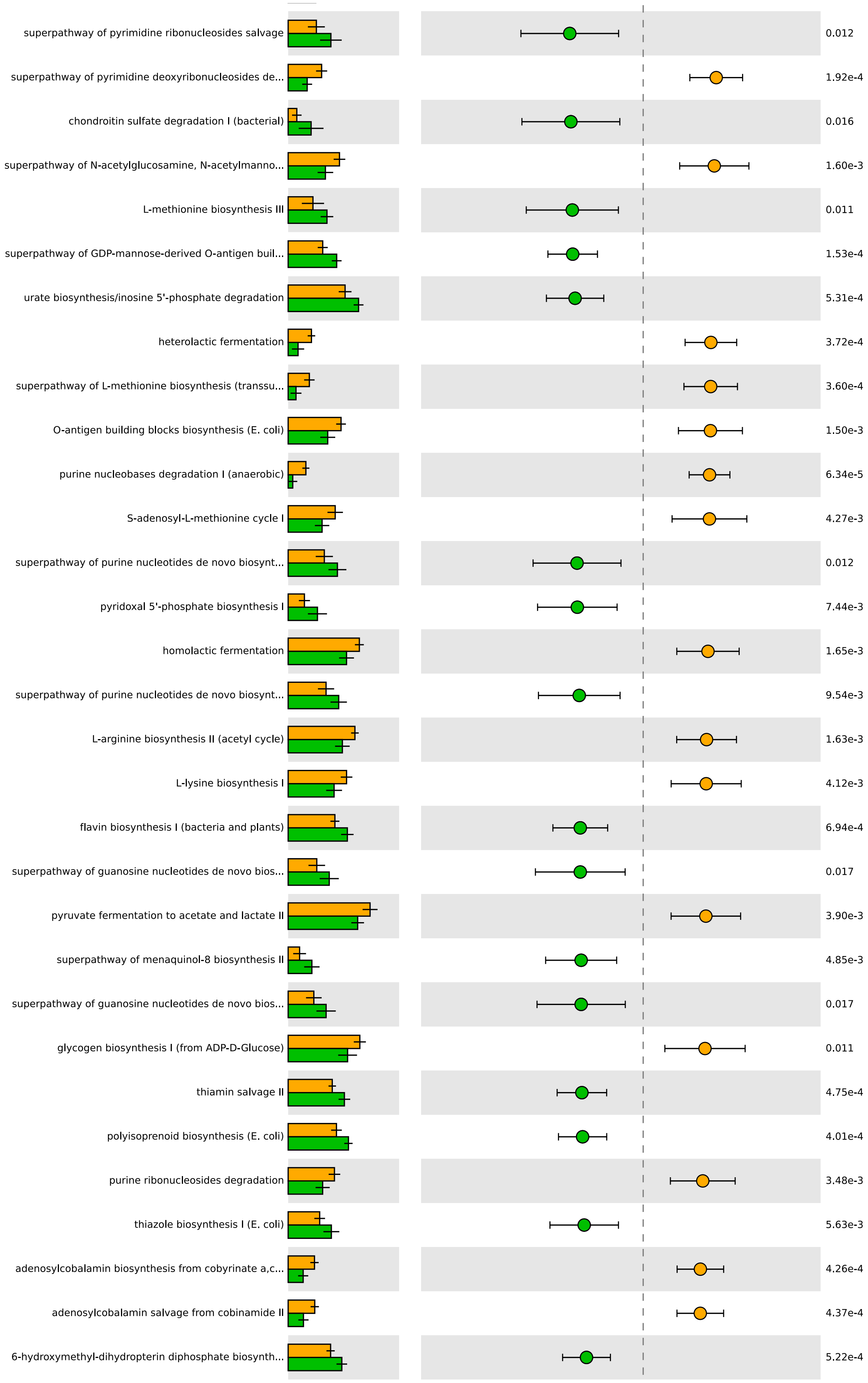
superpathway of pyrimidine ribonucleosides salvage
0.012
superpathway of pyrimidine deoxyribonucleosides de...
1.92e-4
chondroitin sulfate degradation I (bacterial)
0.016
superpathway of N-acetylglucosamine, N-acetylmanno...
1.60e-3
L-methionine biosynthesis III
0.011
superpathway of GDP-mannose-derived O-antigen buil...
1.53e-4
urate biosynthesis/inosine 5'-phosphate degradation
5.31e-4
heterolactic fermentation
3.72e-4
superpathway of L-methionine biosynthesis (transsu...
3.60e-4
O-antigen building blocks biosynthesis (E. coli)
1.50e-3
purine nucleobases degradation I (anaerobic)
6.34e-5
S-adenosyl-L-methionine cycle I
4.27e-3
superpathway of purine nucleotides de novo biosynt...
0.012
pyridoxal 5'-phosphate biosynthesis I
7.44e-3
homolactic fermentation
1.65e-3
superpathway of purine nucleotides de novo biosynt...
9.54e-3
L-arginine biosynthesis II (acetyl cycle)
1.63e-3
L-lysine biosynthesis I
4.12e-3
flavin biosynthesis I (bacteria and plants)
6.94e-4
superpathway of guanosine nucleotides de novo bios...
0.017
pyruvate fermentation to acetate and lactate II
3.90e-3
superpathway of menaquinol-8 biosynthesis II
4.85e-3
superpathway of guanosine nucleotides de novo bios...
0.017
glycogen biosynthesis I (from ADP-D-Glucose)
0.011
thiamin salvage II
4.75e-4
polyisoprenoid biosynthesis (E. coli)
4.01e-4
purine ribonucleosides degradation
3.48e-3
thiazole biosynthesis I (E. coli)
5.63e-3
adenosylcobalamin biosynthesis from cobyrinate a,c...
4.26e-4
adenosylcobalamin salvage from cobinamide II
4.37e-4
6-hydroxymethyl-dihydropterin diphosphate biosynth...
5.22e-4

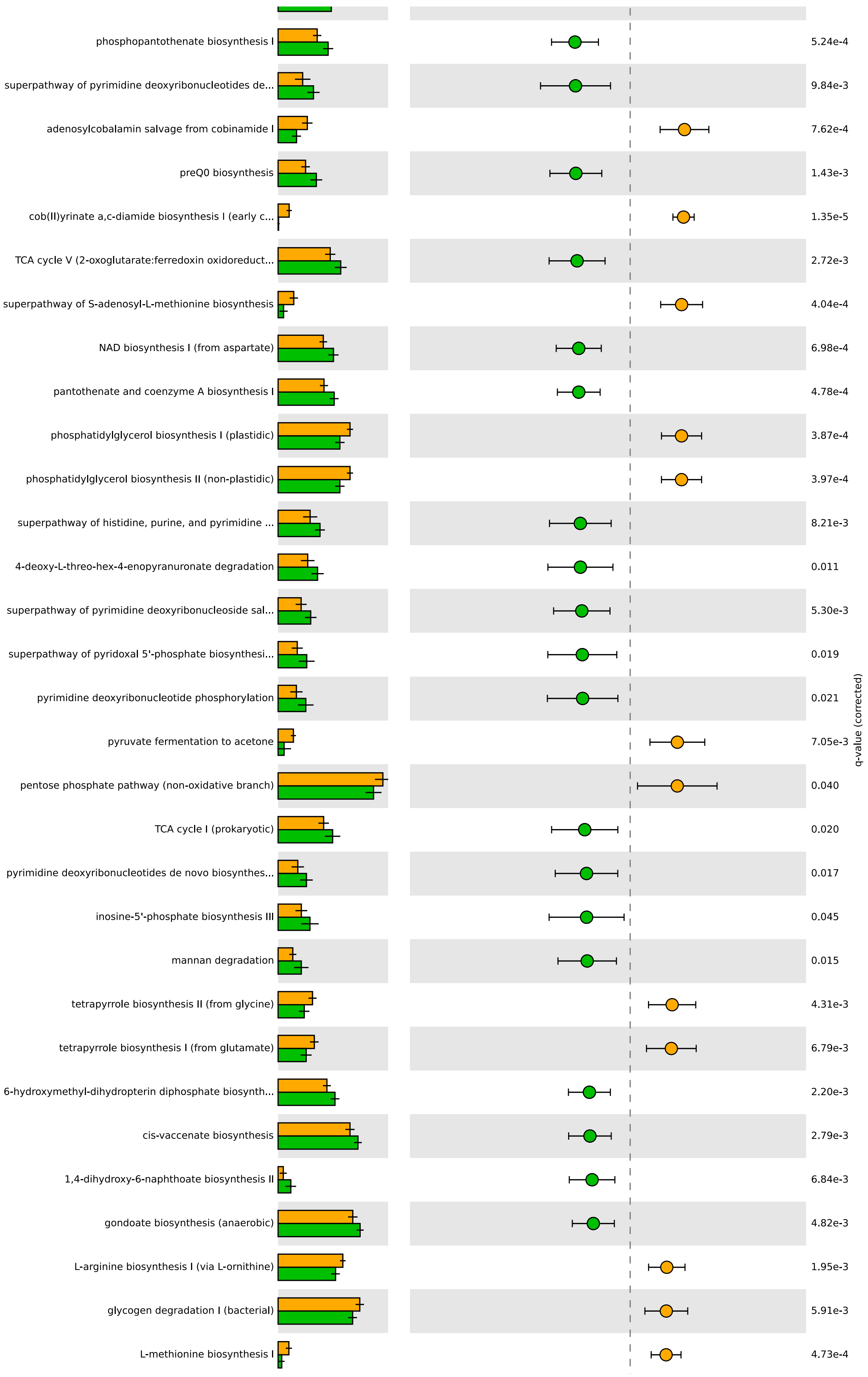

phosphopantothenate biosynthesis I
superpathway of pyrimidine deoxyribonucleotides de...
adenosylcobalamin salvage from cobinamide I
preQ0 biosynthesis
cob(II)yrinate a,c-diamide biosynthesis I (early c...
TCA cycle V (2-oxoglutarate:ferredoxin oxidoreduct...
superpathway of S-adenosyl-L-methionine biosynthesis
NAD biosynthesis I (from aspartate)
pantothenate and coenzyme A biosynthesis I
phosphatidylglycerol biosynthesis I (plastidic)
phosphatidylglycerol biosynthesis II (non-plastidic)
superpathway of histidine, purine, and pyrimidine ...
4-deoxy-L-threo-hex-4-enopyranuronate degradation
superpathway of pyrimidine deoxyribonucleoside sal...
superpathway of pyridoxal 5'-phosphate biosynthesi...
pyrimidine deoxyribonucleotide phosphorylation
pyruvate fermentation to acetone
pentose phosphate pathway (non-oxidative branch)
TCA cycle I (prokaryotic)
pyrimidine deoxyribonucleotides de novo biosynthes...
inosine-5'-phosphate biosynthesis III
mannan degradation
tetrapyrrole biosynthesis II (from glycine)
tetrapyrrole biosynthesis I (from glutamate)
6-hydroxymethyl-dihydropterin diphosphate biosynth...
cis-vaccenate biosynthesis
1,4-dihydroxy-6-naphthoate biosynthesis II
gondoate biosynthesis (anaerobic)
L-arginine biosynthesis I (via L-ornithine)
glycogen degradation I (bacterial)
L-methionine biosynthesis I
5.24e-4
9.84e-3
7.62e-4
1.43e-3
1.35e-5
2.72e-3
4.04e-4
6.98e-4
4.78e-4
3.87e-4
3.97e-4
8.21e-3
0.011
5.30e-3
0.019
0.021
7.05e-3
0.040
0.020
0.017
0.045
0.015
4.31e-3
6.79e-3
2.20e-3
2.79e-3
6.84e-3
4.82e-3
1.95e-3
5.91e-3
4.73e-4
q-value (corrected)

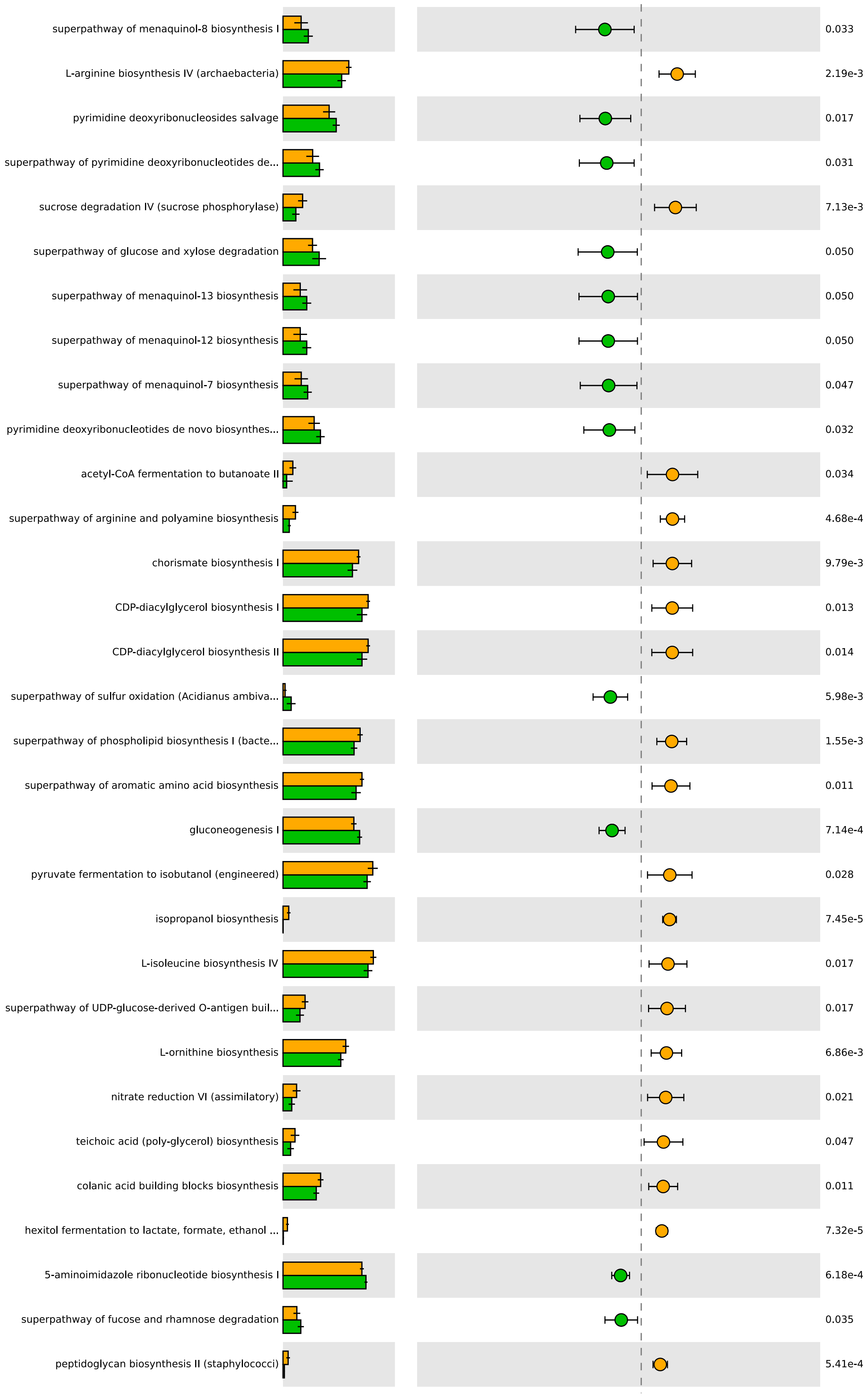
superpathway of menaquinol-8 biosynthesis I
0.033
L-arginine biosynthesis IV (archaebacteria)
2.19e-3
pyrimidine deoxyribonucleosides salvage
0.017
superpathway of pyrimidine deoxyribonucleotides de...
0.031
sucrose degradation IV (sucrose phosphorylase)
7.13e-3
superpathway of glucose and xylose degradation
0.050
superpathway of menaquinol-13 biosynthesis
0.050
superpathway of menaquinol-12 biosynthesis
0.050
superpathway of menaquinol-7 biosynthesis
0.047
pyrimidine deoxyribonucleotides de novo biosynthes...
0.032
acetyl-CoA fermentation to butanoate II
0.034
superpathway of arginine and polyamine biosynthesis
4.68e-4
chorismate biosynthesis I
9.79e-3
CDP-diacylglycerol biosynthesis I
0.013
CDP-diacylglycerol biosynthesis II
0.014
superpathway of sulfur oxidation (Acidianus ambiva...
5.98e-3
superpathway of phospholipid biosynthesis I (bacte...
1.55e-3
superpathway of aromatic amino acid biosynthesis
0.011
gluconeogenesis I
7.14e-4
pyruvate fermentation to isobutanol (engineered)
0.028
isopropanol biosynthesis
7.45e-5
L-isoleucine biosynthesis IV
0.017
superpathway of UDP-glucose-derived O-antigen buil...
0.017
L-ornithine biosynthesis
6.86e-3
nitrate reduction VI (assimilatory)
0.021
teichoic acid (poly-glycerol) biosynthesis
0.047
colanic acid building blocks biosynthesis
0.011
hexitol fermentation to lactate, formate, ethanol ...
7.32e-5
5-aminoimidazole ribonucleotide biosynthesis I
6.18e-4
superpathway of fucose and rhamnose degradation
0.035
peptidoglycan biosynthesis II (staphylococci)
5.41e-4

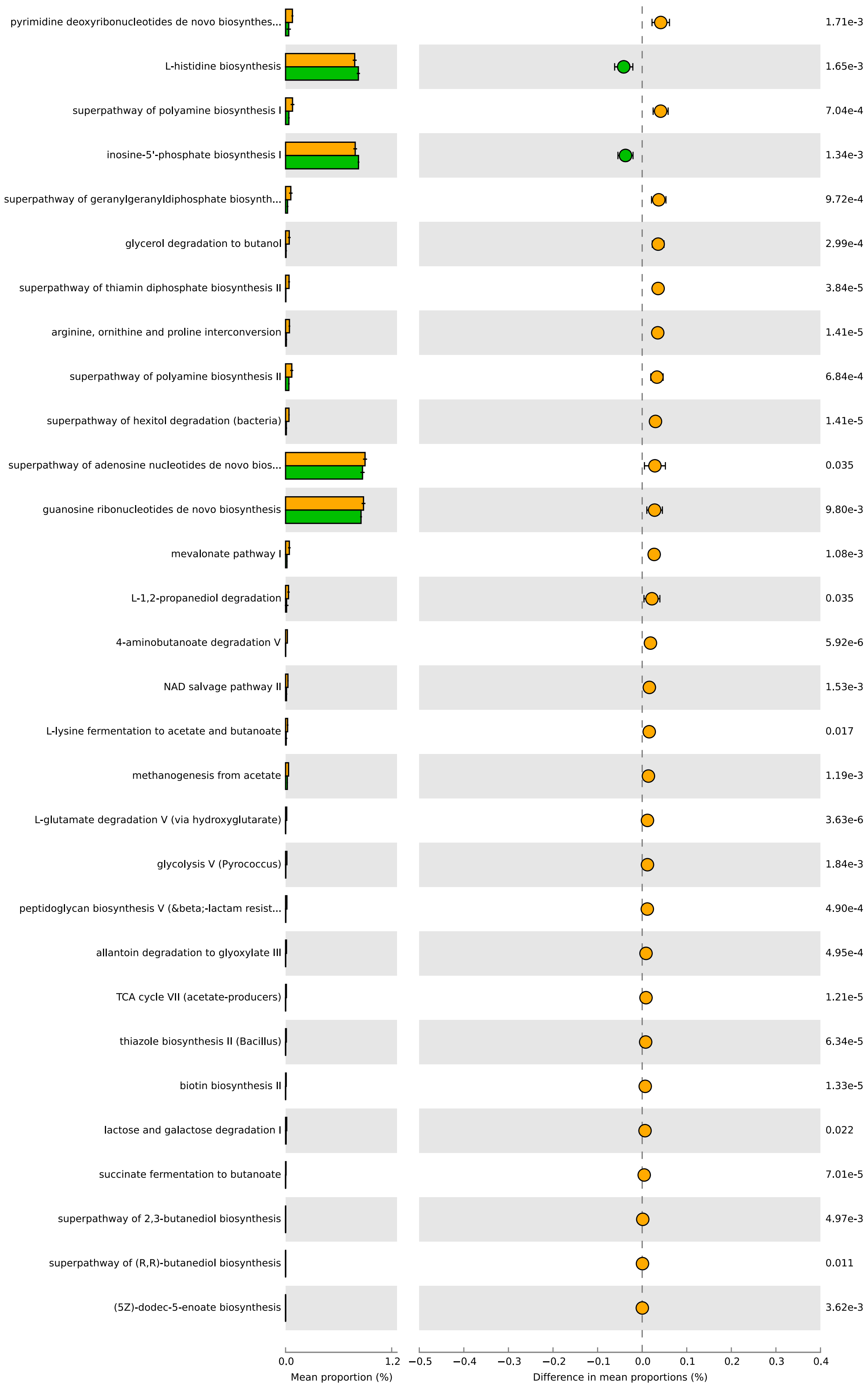

pyrimidine deoxyribonucleotides de novo biosynthes...
L-histidine biosynthesis
superpathway of polyamine biosynthesis I
inosine-5'-phosphate biosynthesis I
superpathway of geranylgeranyldiphosphate biosynth...
glycerol degradation to butanol
superpathway of thiamin diphosphate biosynthesis II
arginine, ornithine and proline interconversion
superpathway of polyamine biosynthesis II
superpathway of hexitol degradation (bacteria)
superpathway of adenosine nucleotides de novo bios...
guanosine ribonucleotides de novo biosynthesis
mevalonate pathway I
L-1,2-propanediol degradation
4-aminobutanoate degradation V
NAD salvage pathway II
L-lysine fermentation to acetate and butanoate
methanogenesis from acetate
L-glutamate degradation V (via hydroxyglutarate)
glycolysis V (Pyrococcus)
peptidoglycan biosynthesis V (β-lactam resist...
allantoin degradation to glyoxylate III
TCA cycle VII (acetate-producers)
thiazole biosynthesis II (Bacillus)
biotin biosynthesis II
lactose and galactose degradation I
succinate fermentation to butanoate
superpathway of 2,3-butanediol biosynthesis
superpathway of (R,R)-butanediol biosynthesis
(5Z)-dodec-5-enoate biosynthesis
1.71e-3
1.65e-3
7.04e-4
1.34e-3
9.72e-4
2.99e-4
3.84e-5
1.41e-5
6.84e-4
1.41e-5
0.035
9.80e-3
1.08e-3
0.035
5.92e-6
1.53e-3
0.017
1.19e-3
3.63e-6
1.84e-3
4.90e-4
4.95e-4
1.21e-5
6.34e-5
1.33e-5
0.022
7.01e-5
4.97e-3
0.011
3.62e-3
0.0
1.2
Mean proportion (%)
−0.5
−0.4
−0.3
−0.2
−0.1
0.0
0.1
0.2
0.3
0.4
Difference in mean proportions (%)

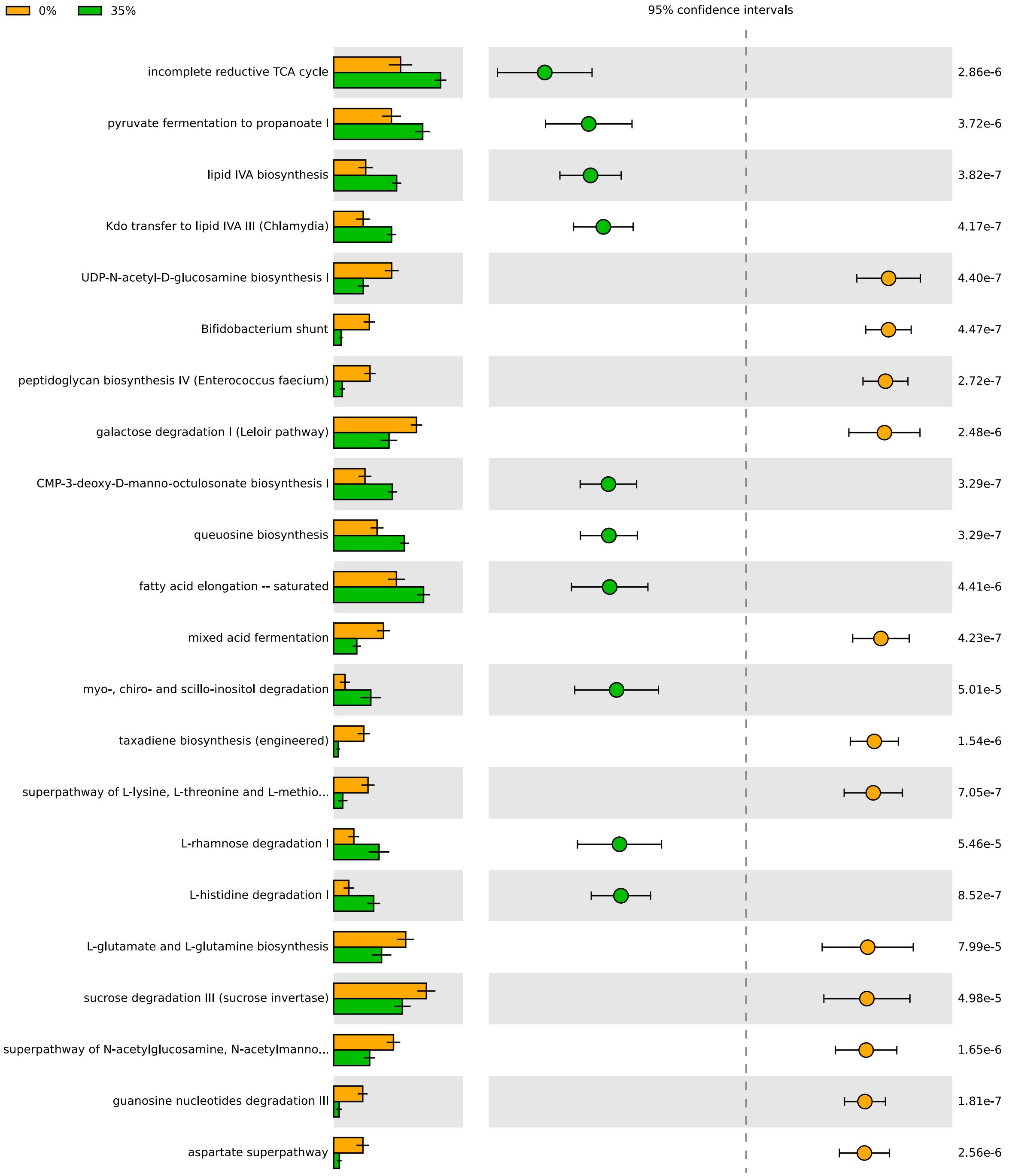

0%
35%
95% confidence intervals
incomplete reductive TCA cycle
2.86e-6
pyruvate fermentation to propanoate I
3.72e-6
lipid IVA biosynthesis
3.82e-7
Kdo transfer to lipid IVA III (Chlamydia)
4.17e-7
UDP-N-acetyl-D-glucosamine biosynthesis I
4.40e-7
Bifidobacterium shunt
4.47e-7
peptidoglycan biosynthesis IV (Enterococcus faecium)
2.72e-7
galactose degradation I (Leloir pathway)
2.48e-6
CMP-3-deoxy-D-manno-octulosonate biosynthesis I
3.29e-7
queuosine biosynthesis
3.29e-7
fatty acid elongation -- saturated
4.41e-6
mixed acid fermentation
4.23e-7
myo-, chiro- and scillo-inositol degradation
5.01e-5
taxadiene biosynthesis (engineered)
1.54e-6
superpathway of L-lysine, L-threonine and L-methio...
7.05e-7
L-rhamnose degradation I
5.46e-5
L-histidine degradation I
8.52e-7
L-glutamate and L-glutamine biosynthesis
7.99e-5
sucrose degradation III (sucrose invertase)
4.98e-5
superpathway of N-acetylglucosamine, N-acetylmanno...
1.65e-6
guanosine nucleotides degradation III
1.81e-7
aspartate superpathway
2.56e-6

b.

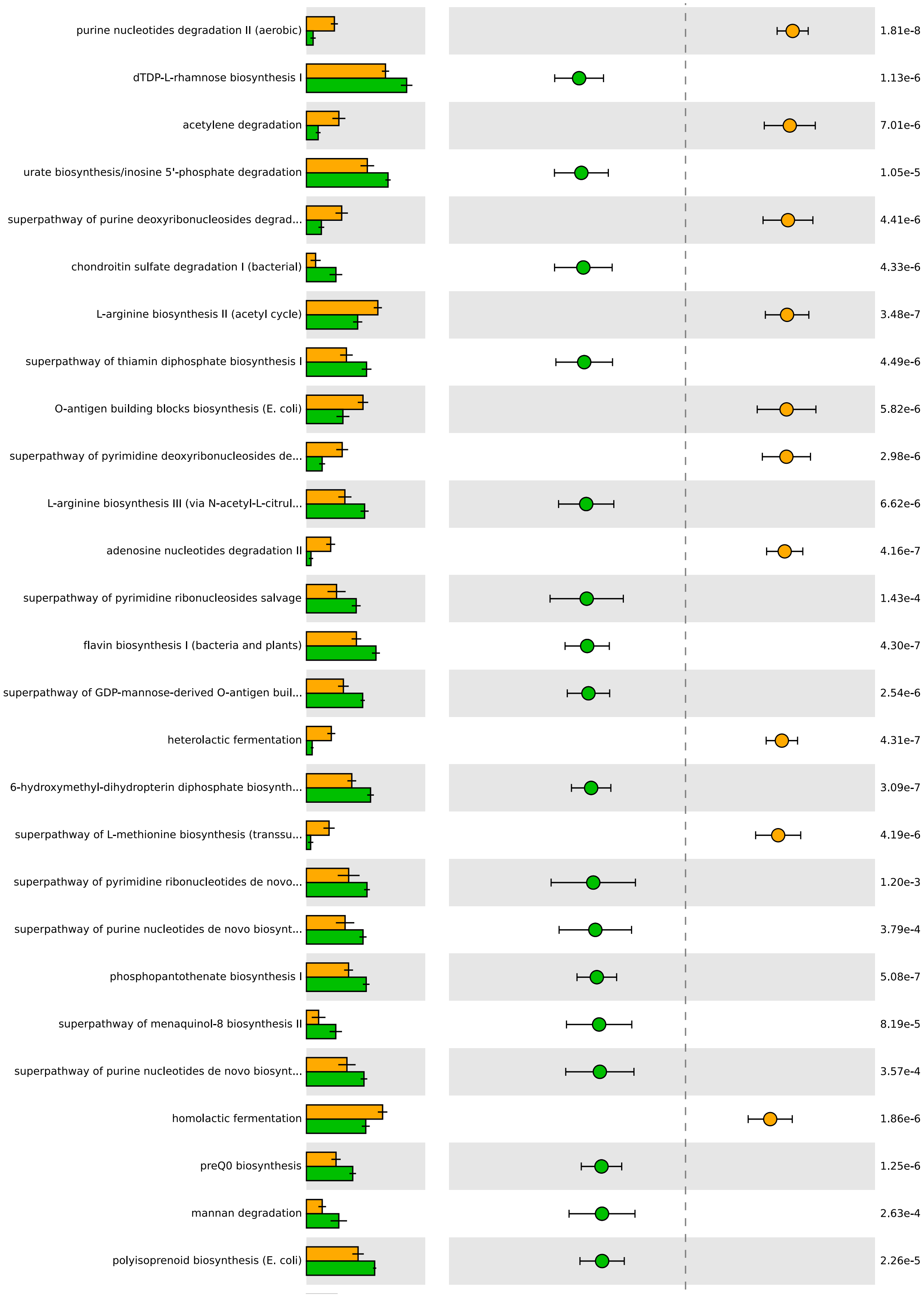

purine nucleotides degradation II (aerobic)
1.81e-8
dTDP-L-rhamnose biosynthesis I
1.13e-6
acetylene degradation
7.01e-6
urate biosynthesis/inosine 5'-phosphate degradation
1.05e-5
superpathway of purine deoxyribonucleosides degrad...
4.41e-6
chondroitin sulfate degradation I (bacterial)
4.33e-6
L-arginine biosynthesis II (acetyl cycle)
3.48e-7
superpathway of thiamin diphosphate biosynthesis I
4.49e-6
O-antigen building blocks biosynthesis (E. coli)
5.82e-6
superpathway of pyrimidine deoxyribonucleosides de...
2.98e-6
L-arginine biosynthesis III (via N-acetyl-L-citrul...
6.62e-6
adenosine nucleotides degradation II
4.16e-7
superpathway of pyrimidine ribonucleosides salvage
1.43e-4
flavin biosynthesis I (bacteria and plants)
4.30e-7
superpathway of GDP-mannose-derived O-antigen buil...
2.54e-6
heterolactic fermentation
4.31e-7
6-hydroxymethyl-dihydropterin diphosphate biosynth...
3.09e-7
superpathway of L-methionine biosynthesis (transsu...
4.19e-6
superpathway of pyrimidine ribonucleotides de novo...
1.20e-3
superpathway of purine nucleotides de novo biosynt...
3.79e-4
phosphopantothenate biosynthesis I
5.08e-7
superpathway of menaquinol-8 biosynthesis II
8.19e-5
superpathway of purine nucleotides de novo biosynt...
3.57e-4
homolactic fermentation
1.86e-6
preQ0 biosynthesis
1.25e-6
mannan degradation
2.63e-4
polyisoprenoid biosynthesis (E. coli)
2.26e-5

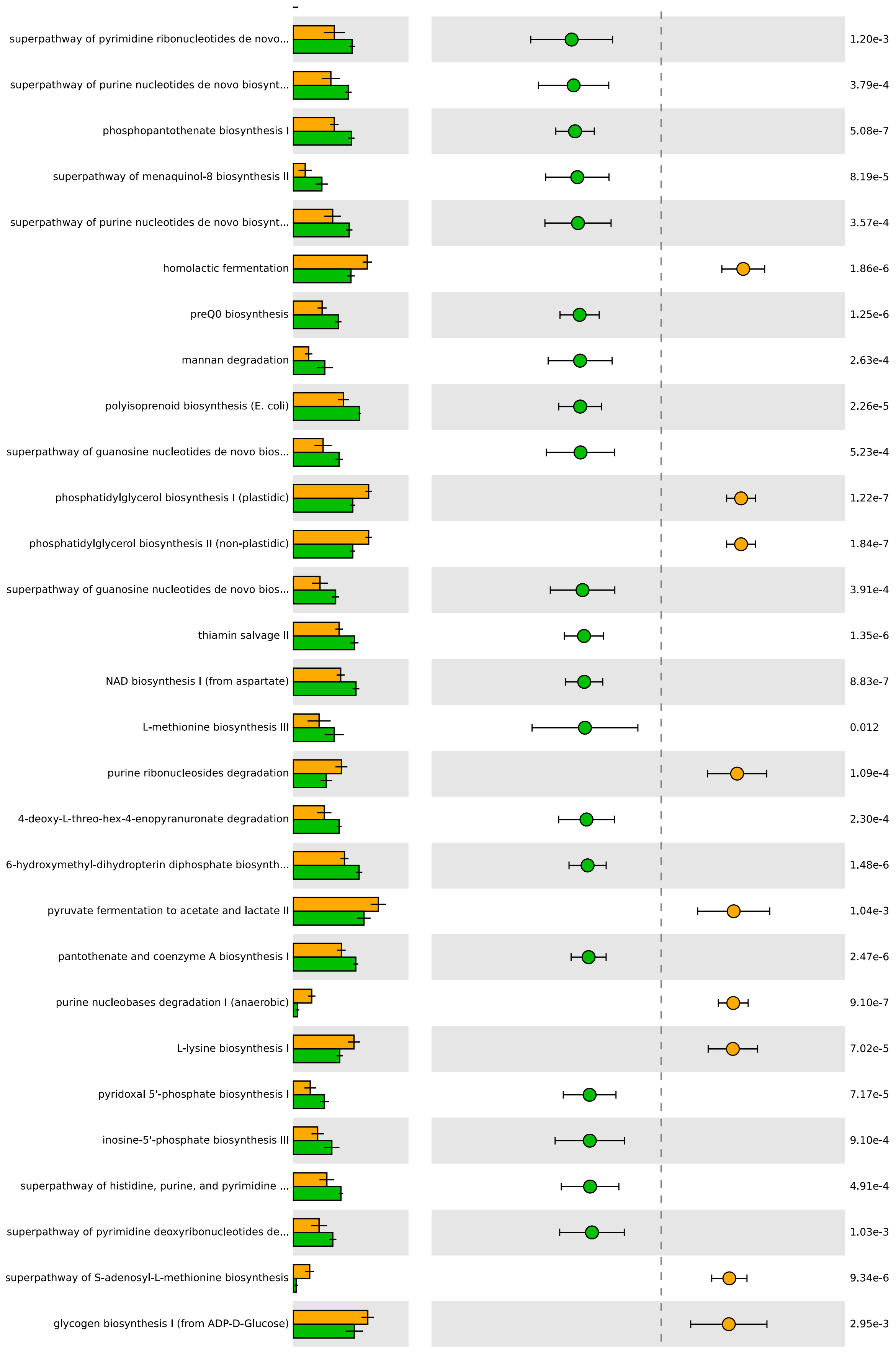

superpathway of pyrimidine ribonucleotides de novo... 1.20e-3
superpathway of purine nucleotides de novo biosynt... 3.79e-4
phosphopantothenate biosynthesis I 5.08e-7
superpathway of menaquinol-8 biosynthesis II 8.19e-5
superpathway of purine nucleotides de novo biosynt... 3.57e-4
homolactic fermentation 1.86e-6
preQ0 biosynthesis 1.25e-6
mannan degradation 2.63e-4
polyisoprenoid biosynthesis (E. coli) 2.26e-5
superpathway of guanosine nucleotides de novo bios... 5.23e-4
phosphatidylglycerol biosynthesis I (plastidic) 1.22e-7
phosphatidylglycerol biosynthesis II (non-plastidic) 1.84e-7
superpathway of guanosine nucleotides de novo bios... 3.91e-4
thiamin salvage II 1.35e-6
NAD biosynthesis I (from aspartate) 8.83e-7
L-methionine biosynthesis III 0.012
purine ribonucleosides degradation 1.09e-4
4-deoxy-L-threo-hex-4-enopyranuronate degradation 2.30e-4
6-hydroxymethyl-dihydropterin diphosphate biosynth... 1.48e-6
pyruvate fermentation to acetate and lactate II 1.04e-3
pantothenate and coenzyme A biosynthesis I 2.47e-6
purine nucleobases degradation I (anaerobic) 9.10e-7
L-lysine biosynthesis I 7.02e-5
pyridoxal 5'-phosphate biosynthesis I 7.17e-5
inosine-5'-phosphate biosynthesis III 9.10e-4
superpathway of histidine, purine, and pyrimidine ... 4.91e-4
superpathway of pyrimidine deoxyribonucleotides de... 1.03e-3
superpathway of S-adenosyl-L-methionine biosynthesis 9.34e-6
glycogen biosynthesis I (from ADP-D-Glucose) 2.95e-3

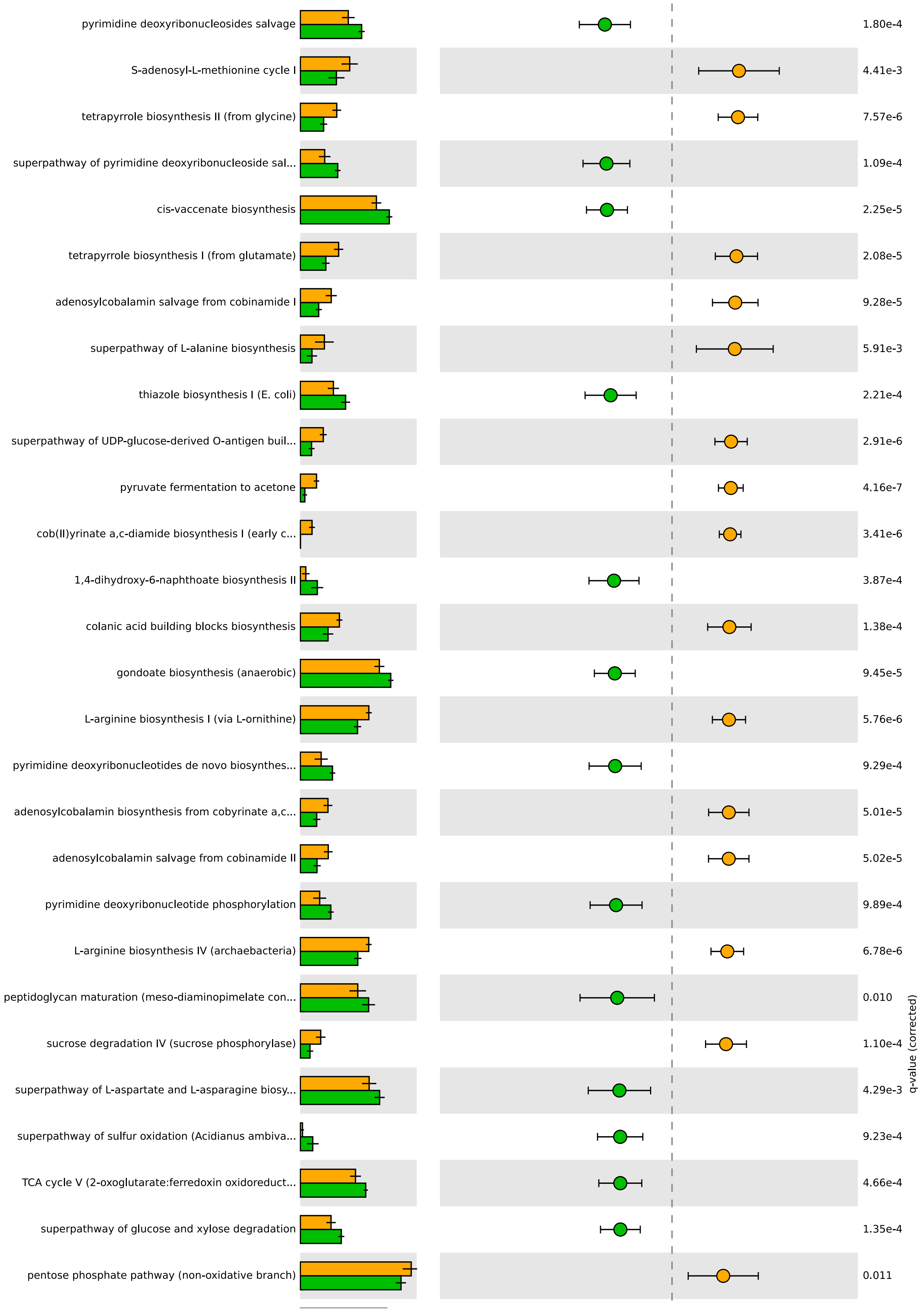

pyrimidine deoxyribonucleosides salvage
S-adenosyl-L-methionine cycle I
tetrapyrrole biosynthesis II (from glycine)
superpathway of pyrimidine deoxyribonucleoside sal...
cis-vaccenate biosynthesis
tetrapyrrole biosynthesis I (from glutamate)
adenosylcobalamin salvage from cobinamide I
superpathway of L-alanine biosynthesis
thiazole biosynthesis I (E. coli)
superpathway of UDP-glucose-derived O-antigen buil...
pyruvate fermentation to acetone
cob(II)yrinate a,c-diamide biosynthesis I (early c...
1,4-dihydroxy-6-naphthoate biosynthesis II
colanic acid building blocks biosynthesis
gondoate biosynthesis (anaerobic)
L-arginine biosynthesis I (via L-ornithine)
pyrimidine deoxyribonucleotides de novo biosynthes...
adenosylcobalamin biosynthesis from cobyrinate a,c...
adenosylcobalamin salvage from cobinamide II
pyrimidine deoxyribonucleotide phosphorylation
L-arginine biosynthesis IV (archaebacteria)
peptidoglycan maturation (meso-diaminopimelate con...
sucrose degradation IV (sucrose phosphorylase)
superpathway of L-aspartate and L-asparagine biosy...
superpathway of sulfur oxidation (Acidianus ambiva...
TCA cycle V (2-oxoglutarate:ferredoxin oxidoreduct...
superpathway of glucose and xylose degradation
pentose phosphate pathway (non-oxidative branch)
1.80e-4
4.41e-3
7.57e-6
1.09e-4
2.25e-5
2.08e-5
9.28e-5
5.91e-3
2.21e-4
2.91e-6
4.16e-7
3.41e-6
3.87e-4
1.38e-4
9.45e-5
5.76e-6
9.29e-4
5.01e-5
5.02e-5
9.89e-4
6.78e-6
0.010
1.10e-4
4.29e-3
9.23e-4
4.66e-4
1.35e-4
0.011
q-value (corrected)

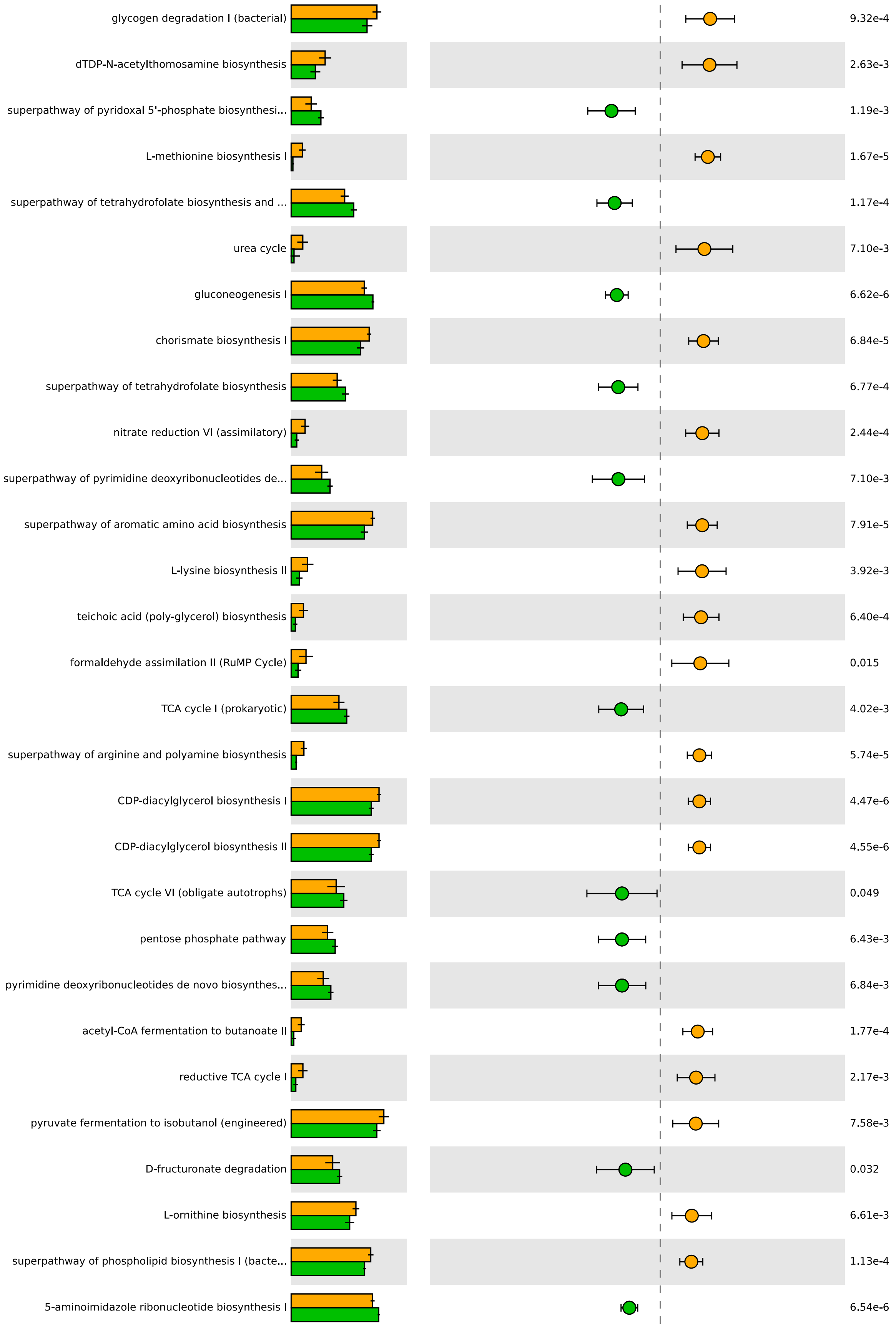
glycogen degradation I (bacterial)
dTDP-N-acetylthomosamine biosynthesis
superpathway of pyridoxal 5'-phosphate biosynthesi...
L-methionine biosynthesis I
superpathway of tetrahydrofolate biosynthesis and ...
urea cycle
gluconeogenesis I
chorismate biosynthesis I
superpathway of tetrahydrofolate biosynthesis
nitrate reduction VI (assimilatory)
superpathway of pyrimidine deoxyribonucleotides de...
superpathway of aromatic amino acid biosynthesis
L-lysine biosynthesis II
teichoic acid (poly-glycerol) biosynthesis
formaldehyde assimilation II (RuMP Cycle)
TCA cycle I (prokaryotic)
superpathway of arginine and polyamine biosynthesis
CDP-diacylglycerol biosynthesis I
CDP-diacylglycerol biosynthesis II
TCA cycle VI (obligate autotrophs)
pentose phosphate pathway
pyrimidine deoxyribonucleotides de novo biosynthes...
acetyl-CoA fermentation to butanoate II
reductive TCA cycle I
pyruvate fermentation to isobutanol (engineered)
D-fructuronate degradation
L-ornithine biosynthesis
superpathway of phospholipid biosynthesis I (bacte...
5-aminoimidazole ribonucleotide biosynthesis I
9.32e-4
2.63e-3
1.19e-3
1.67e-5
1.17e-4
7.10e-3
6.62e-6
6.84e-5
6.77e-4
2.44e-4
7.10e-3
7.91e-5
3.92e-3
6.40e-4
0.015
4.02e-3
5.74e-5
4.47e-6
4.55e-6
0.049
6.43e-3
6.84e-3
1.77e-4
2.17e-3
7.58e-3
0.032
6.61e-3
1.13e-4
6.54e-6

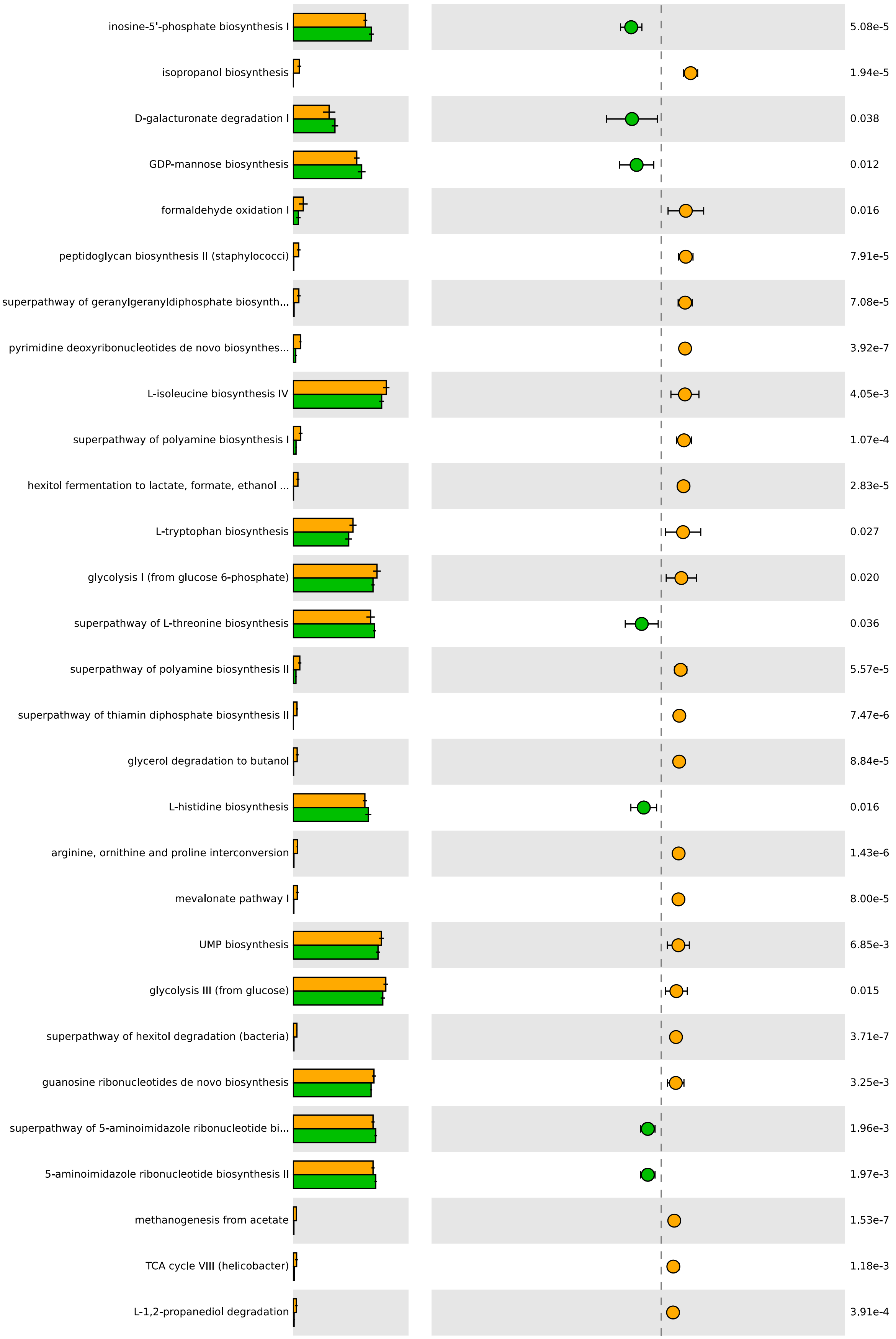
inosine-5'-phosphate biosynthesis I
isopropanol biosynthesis
D-galacturonate degradation I
GDP-mannose biosynthesis
formaldehyde oxidation I
peptidoglycan biosynthesis II (staphylococci)
superpathway of geranylgeranyldiphosphate biosynth...
pyrimidine deoxyribonucleotides de novo biosynthes...
L-isoleucine biosynthesis IV
superpathway of polyamine biosynthesis I
hexitol fermentation to lactate, formate, ethanol ...
L-tryptophan biosynthesis
glycolysis I (from glucose 6-phosphate)
superpathway of L-threonine biosynthesis
superpathway of polyamine biosynthesis II
superpathway of thiamin diphosphate biosynthesis II
glycerol degradation to butanol
L-histidine biosynthesis
arginine, ornithine and proline interconversion
mevalonate pathway I
UMP biosynthesis
glycolysis III (from glucose)
superpathway of hexitol degradation (bacteria)
guanosine ribonucleotides de novo biosynthesis
superpathway of 5-aminoimidazole ribonucleotide bi...
5-aminoimidazole ribonucleotide biosynthesis II
methanogenesis from acetate
TCA cycle VIII (helicobacter)
L-1,2-propanediol degradation
5.08e-5
1.94e-5
0.038
0.012
0.016
7.91e-5
7.08e-5
3.92e-7
4.05e-3
1.07e-4
2.83e-5
0.027
0.020
0.036
5.57e-5
7.47e-6
8.84e-5
0.016
1.43e-6
8.00e-5
6.85e-3
0.015
3.71e-7
3.25e-3
1.96e-3
1.97e-3
1.53e-7
1.18e-3
3.91e-4

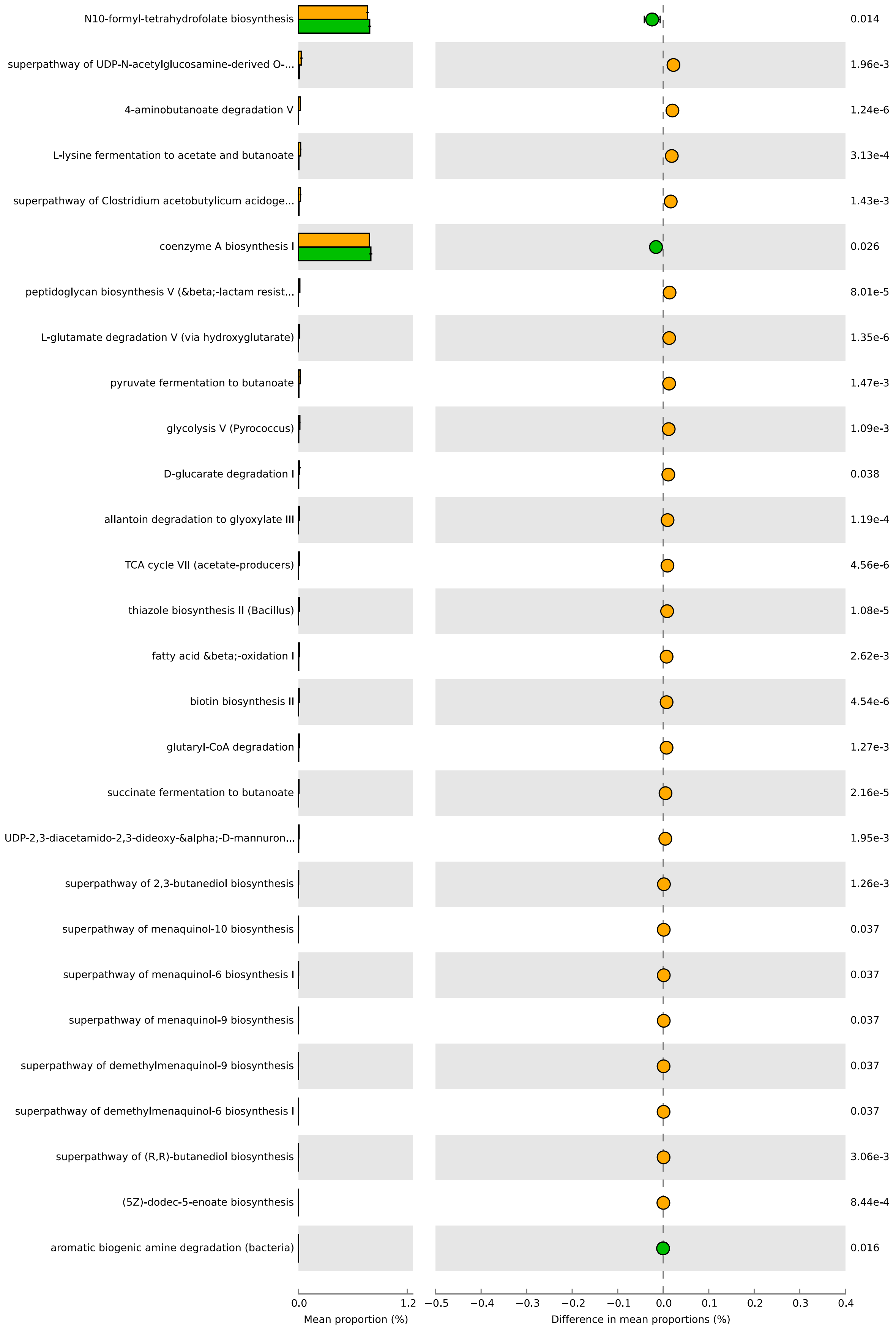

N10-formyl-tetrahydrofolate biosynthesis
superpathway of UDP-N-acetylglucosamine-derived O-...
4-aminobutanoate degradation V
L-lysine fermentation to acetate and butanoate
superpathway of Clostridium acetobutylicum acidoge...
coenzyme A biosynthesis I
peptidoglycan biosynthesis V (β-lactam resist...
L-glutamate degradation V (via hydroxyglutarate)
pyruvate fermentation to butanoate
glycolysis V (Pyrococcus)
D-glucarate degradation I
allantoin degradation to glyoxylate III
TCA cycle VII (acetate-producers)
thiazole biosynthesis II (Bacillus)
fatty acid β-oxidation I
biotin biosynthesis II
glutaryl-CoA degradation
succinate fermentation to butanoate
UDP-2,3-diacetamido-2,3-dideoxy-α-D-mannuron...
superpathway of 2,3-butanediol biosynthesis
superpathway of menaquinol-10 biosynthesis
superpathway of menaquinol-6 biosynthesis I
superpathway of menaquinol-9 biosynthesis
superpathway of demethylmenaquinol-9 biosynthesis
superpathway of demethylmenaquinol-6 biosynthesis I
superpathway of (R,R)-butanediol biosynthesis
(5Z)-dodec-5-enoate biosynthesis
aromatic biogenic amine degradation (bacteria)
0.014
1.96e-3
1.24e-6
3.13e-4
1.43e-3
0.026
8.01e-5
1.35e-6
1.47e-3
1.09e-3
0.038
1.19e-4
4.56e-6
1.08e-5
2.62e-3
4.54e-6
1.27e-3
2.16e-5
1.95e-3
1.26e-3
0.037
0.037
0.037
0.037
0.037
3.06e-3
8.44e-4
0.016
0.0
1.2
Mean proportion (%)
−0.5
−0.4
−0.3
−0.2
−0.1
0.0
0.1
0.2
0.3
0.4
Difference in mean proportions (%)

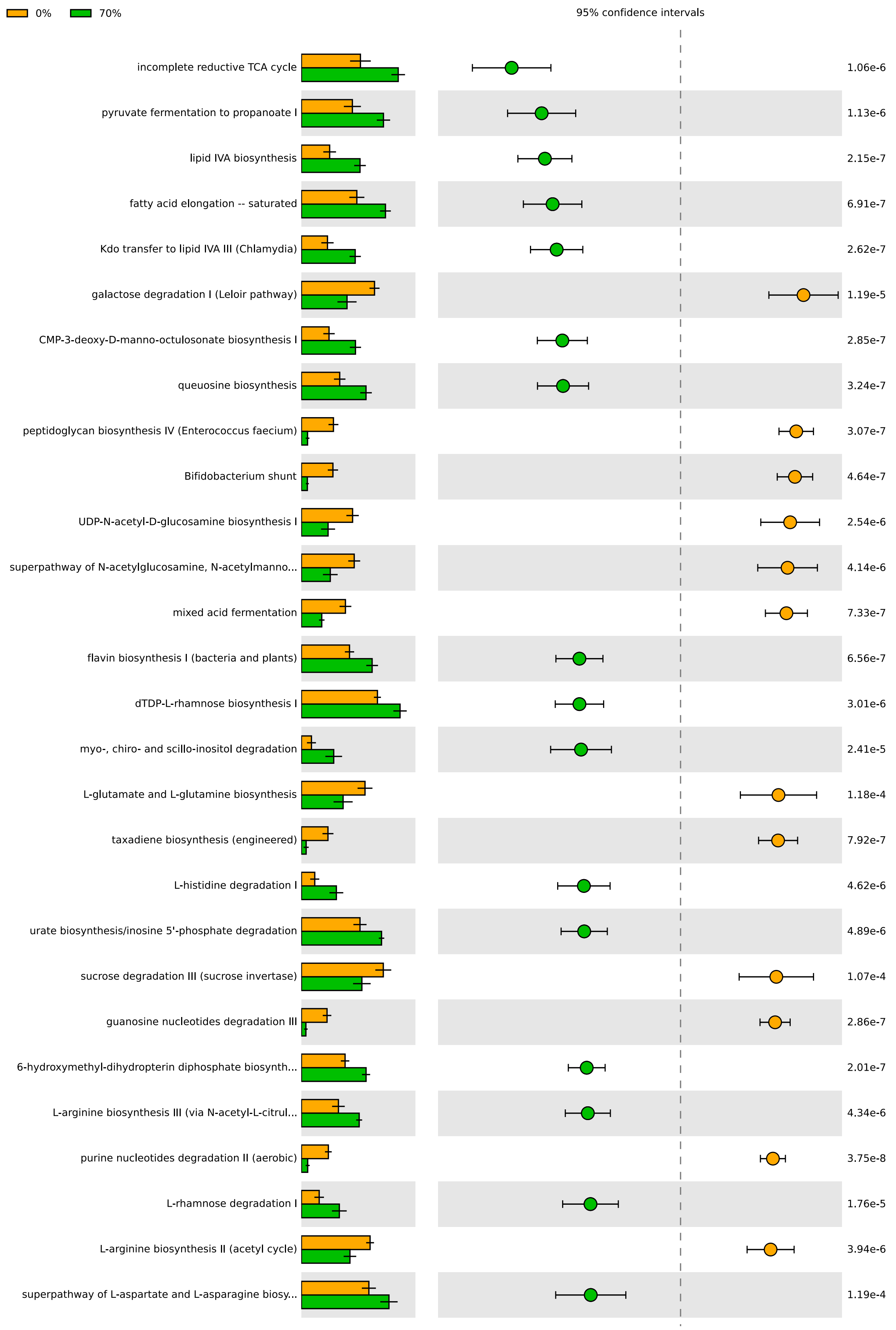
0%
70%
95% confidence intervals
incomplete reductive TCA cycle
1.06e-6
pyruvate fermentation to propanoate I
1.13e-6
lipid IVA biosynthesis
2.15e-7
fatty acid elongation -- saturated
6.91e-7
Kdo transfer to lipid IVA III (Chlamydia)
2.62e-7
galactose degradation I (Leloir pathway)
1.19e-5
CMP-3-deoxy-D-manno-octulosonate biosynthesis I
2.85e-7
queuosine biosynthesis
3.24e-7
peptidoglycan biosynthesis IV (Enterococcus faecium)
3.07e-7
Bifidobacterium shunt
4.64e-7
UDP-N-acetyl-D-glucosamine biosynthesis I
2.54e-6
superpathway of N-acetylglucosamine, N-acetylmanno...
4.14e-6
mixed acid fermentation
7.33e-7
flavin biosynthesis I (bacteria and plants)
6.56e-7
dTDP-L-rhamnose biosynthesis I
3.01e-6
myo-, chiro- and scillo-inositol degradation
2.41e-5
L-glutamate and L-glutamine biosynthesis
1.18e-4
taxadiene biosynthesis (engineered)
7.92e-7
L-histidine degradation I
4.62e-6
urate biosynthesis/inosine 5'-phosphate degradation
4.89e-6
sucrose degradation III (sucrose invertase)
1.07e-4
guanosine nucleotides degradation III
2.86e-7
6-hydroxymethyl-dihydropterin diphosphate biosynth...
2.01e-7
L-arginine biosynthesis III (via N-acetyl-L-citrul...
4.34e-6
purine nucleotides degradation II (aerobic)
3.75e-8
L-rhamnose degradation I
1.76e-5
L-arginine biosynthesis II (acetyl cycle)
3.94e-6
superpathway of L-aspartate and L-asparagine biosy...
1.19e-4

c.

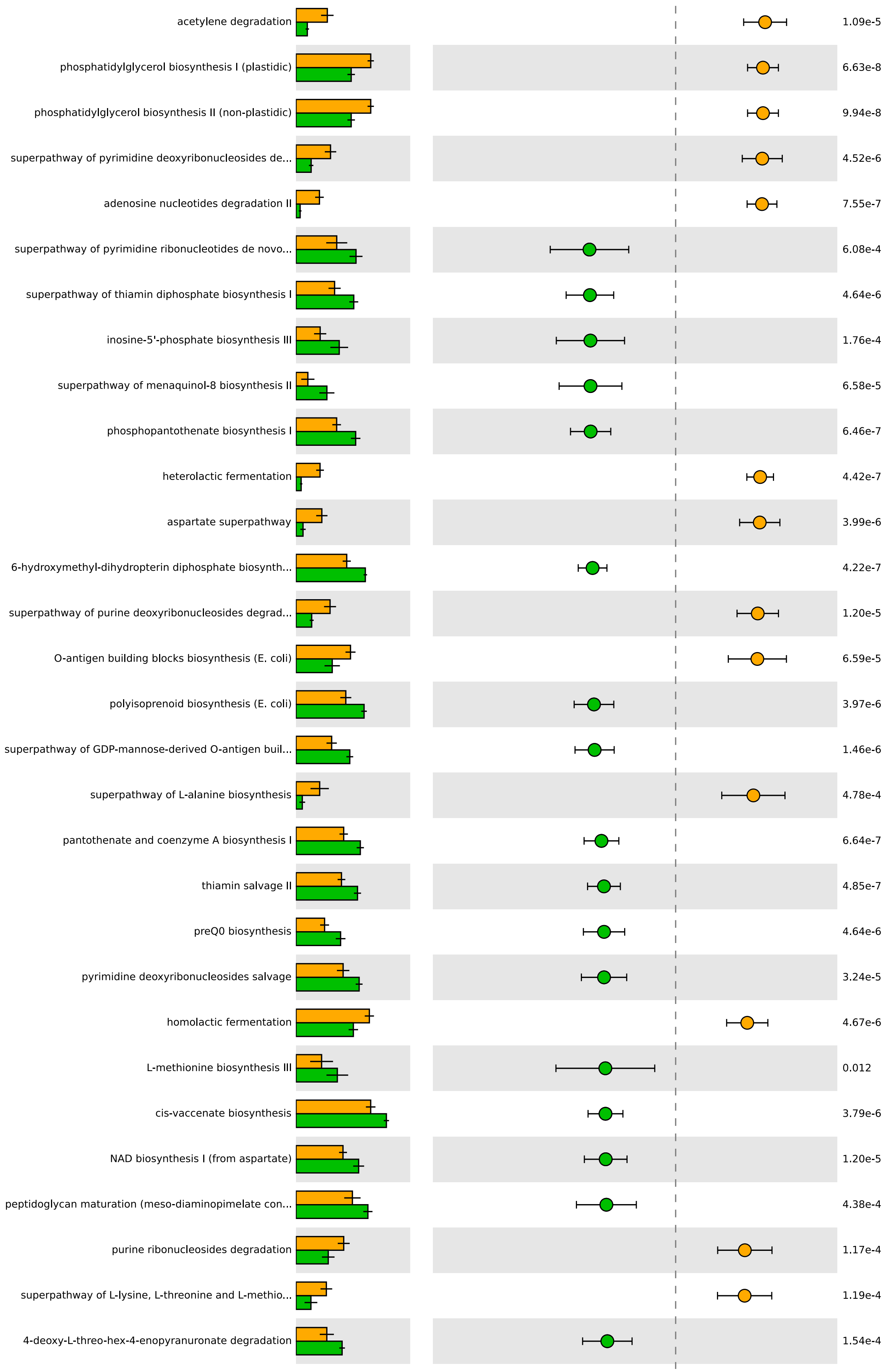

acetylene degradation
phosphatidylglycerol biosynthesis I (plastidic)
phosphatidylglycerol biosynthesis II (non-plastidic)
superpathway of pyrimidine deoxyribonucleosides de...
adenosine nucleotides degradation II
superpathway of pyrimidine ribonucleotides de novo...
superpathway of thiamin diphosphate biosynthesis I
inosine-5'-phosphate biosynthesis III
superpathway of menaquinol-8 biosynthesis II
phosphopantothenate biosynthesis I
heterolactic fermentation
aspartate superpathway
6-hydroxymethyl-dihydropterin diphosphate biosynth...
superpathway of purine deoxyribonucleosides degrad...
O-antigen building blocks biosynthesis (E. coli)
polyisoprenoid biosynthesis (E. coli)
superpathway of GDP-mannose-derived O-antigen buil...
superpathway of L-alanine biosynthesis
pantothenate and coenzyme A biosynthesis I
thiamin salvage II
preQ0 biosynthesis
pyrimidine deoxyribonucleosides salvage
homolactic fermentation
L-methionine biosynthesis III
cis-vaccenate biosynthesis
NAD biosynthesis I (from aspartate)
peptidoglycan maturation (meso-diaminopimelate con...
purine ribonucleosides degradation
superpathway of L-lysine, L-threonine and L-methio...
4-deoxy-L-threo-hex-4-enopyranuronate degradation
1.09e-5
6.63e-8
9.94e-8
4.52e-6
7.55e-7
6.08e-4
4.64e-6
1.76e-4
6.58e-5
6.46e-7
4.42e-7
3.99e-6
4.22e-7
1.20e-5
6.59e-5
3.97e-6
1.46e-6
4.78e-4
6.64e-7
4.85e-7
4.64e-6
3.24e-5
4.67e-6
0.012
3.79e-6
1.20e-5
4.38e-4
1.17e-4
1.19e-4
1.54e-4

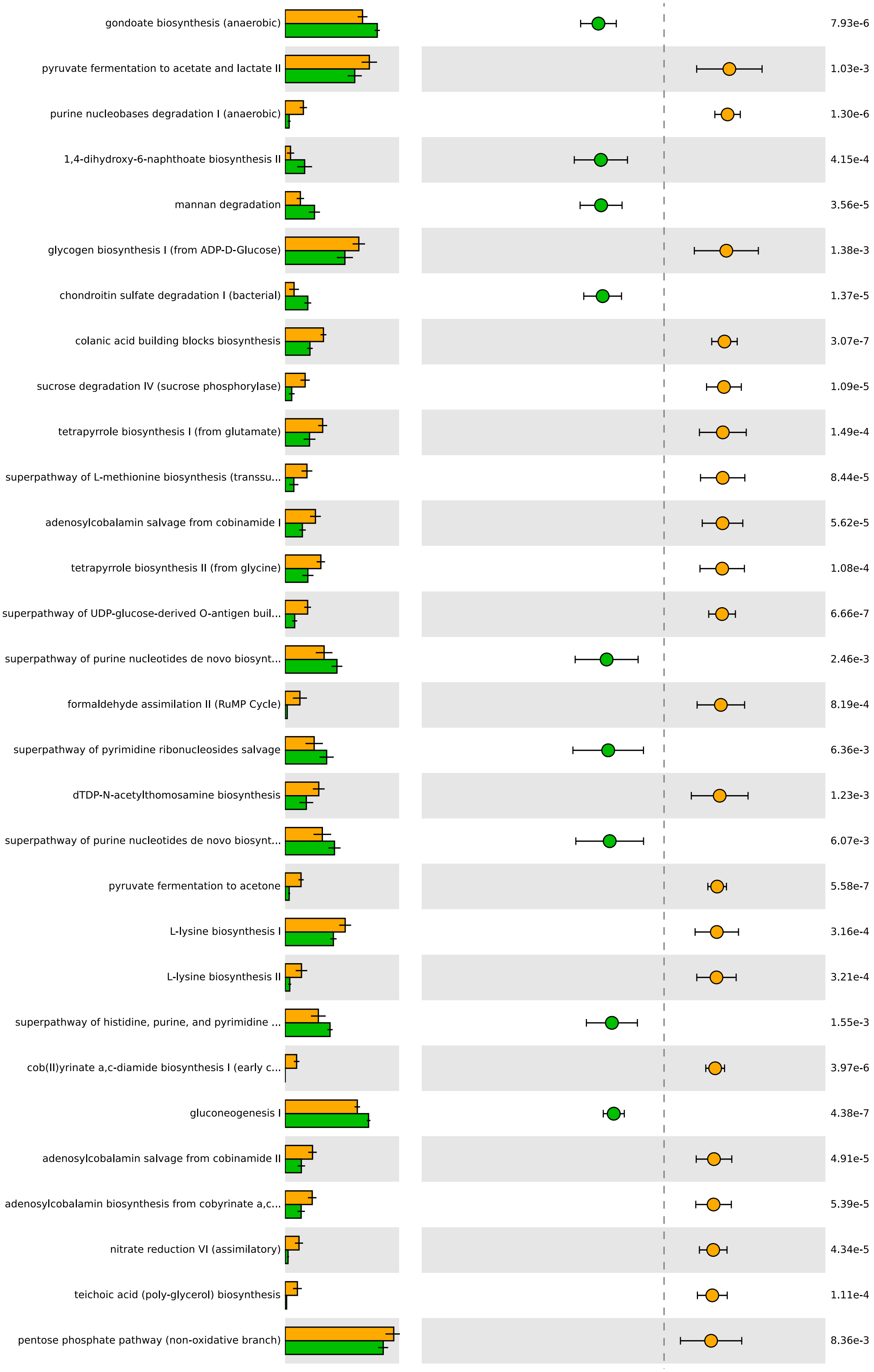

gondoate biosynthesis (anaerobic)
7.93e-6
pyruvate fermentation to acetate and lactate II
1.03e-3
purine nucleobases degradation I (anaerobic)
1.30e-6
1,4-dihydroxy-6-naphthoate biosynthesis II
4.15e-4
mannan degradation
3.56e-5
glycogen biosynthesis I (from ADP-D-Glucose)
1.38e-3
chondroitin sulfate degradation I (bacterial)
1.37e-5
colanic acid building blocks biosynthesis
3.07e-7
sucrose degradation IV (sucrose phosphorylase)
1.09e-5
tetrapyrrole biosynthesis I (from glutamate)
1.49e-4
superpathway of L-methionine biosynthesis (transsu...
8.44e-5
adenosylcobalamin salvage from cobinamide I
5.62e-5
tetrapyrrole biosynthesis II (from glycine)
1.08e-4
superpathway of UDP-glucose-derived O-antigen buil...
6.66e-7
superpathway of purine nucleotides de novo biosynt...
2.46e-3
formaldehyde assimilation II (RuMP Cycle)
8.19e-4
superpathway of pyrimidine ribonucleosides salvage
6.36e-3
dTDP-N-acetylthomosamine biosynthesis
1.23e-3
superpathway of purine nucleotides de novo biosynt...
6.07e-3
pyruvate fermentation to acetone
5.58e-7
L-lysine biosynthesis I
3.16e-4
L-lysine biosynthesis II
3.21e-4
superpathway of histidine, purine, and pyrimidine ...
1.55e-3
cob(II)yrinate a,c-diamide biosynthesis I (early c...
3.97e-6
gluconeogenesis I
4.38e-7
adenosylcobalamin salvage from cobinamide II
4.91e-5
adenosylcobalamin biosynthesis from cobyrinate a,c...
5.39e-5
nitrate reduction VI (assimilatory)
4.34e-5
teichoic acid (poly-glycerol) biosynthesis
1.11e-4
pentose phosphate pathway (non-oxidative branch)
8.36e-3

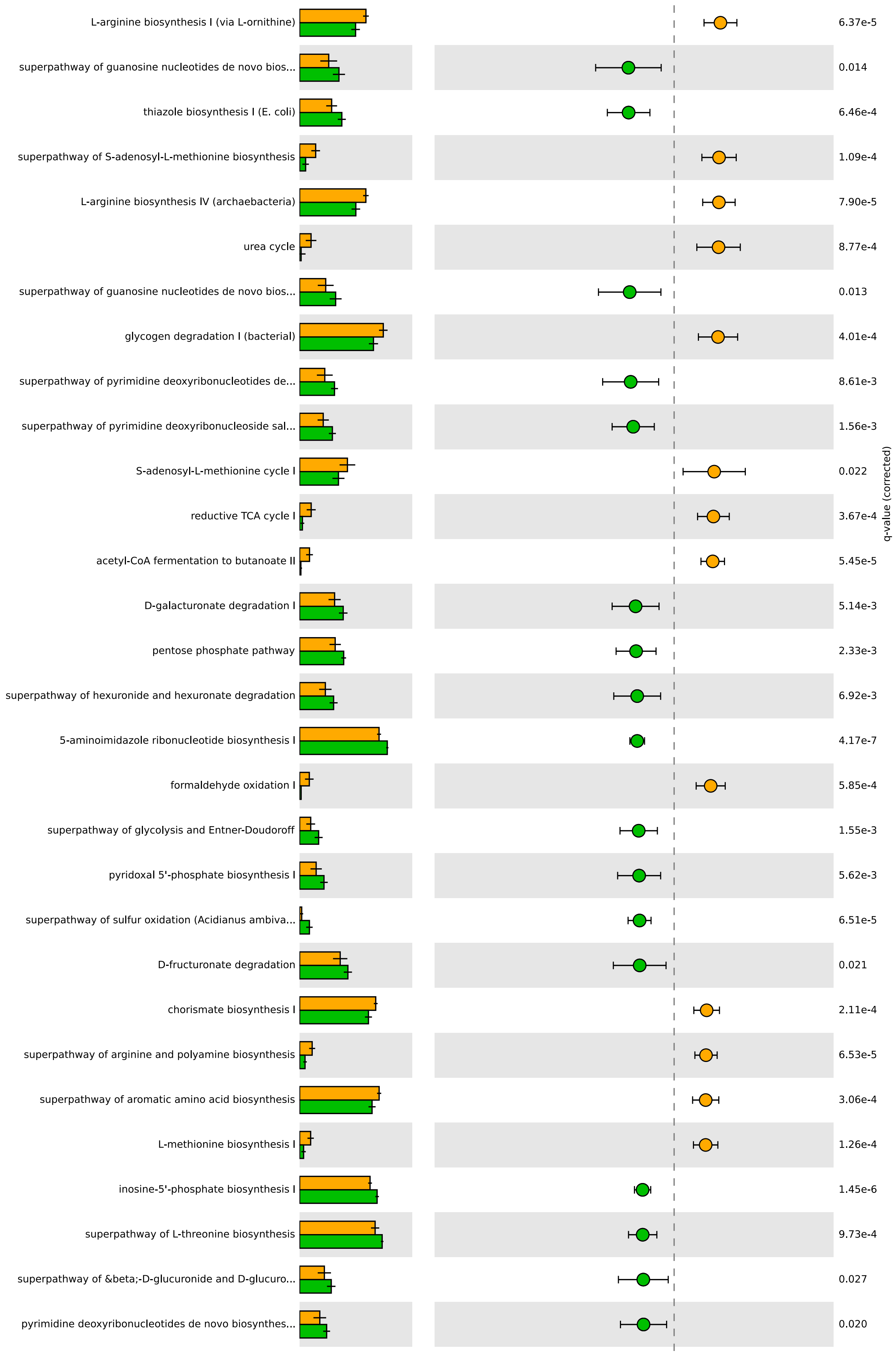
L-arginine biosynthesis I (via L-ornithine)
superpathway of guanosine nucleotides de novo bios...
thiazole biosynthesis I (E. coli)
superpathway of S-adenosyl-L-methionine biosynthesis
L-arginine biosynthesis IV (archaebacteria)
urea cycle
superpathway of guanosine nucleotides de novo bios...
glycogen degradation I (bacterial)
superpathway of pyrimidine deoxyribonucleotides de...
superpathway of pyrimidine deoxyribonucleoside sal...
S-adenosyl-L-methionine cycle I
reductive TCA cycle I
acetyl-CoA fermentation to butanoate II
D-galacturonate degradation I
pentose phosphate pathway
superpathway of hexuronide and hexuronate degradation
5-aminoimidazole ribonucleotide biosynthesis I
formaldehyde oxidation I
superpathway of glycolysis and Entner-Doudoroff
pyridoxal 5'-phosphate biosynthesis I
superpathway of sulfur oxidation (Acidianus ambiva...
D-fructuronate degradation
chorismate biosynthesis I
superpathway of arginine and polyamine biosynthesis
superpathway of aromatic amino acid biosynthesis
L-methionine biosynthesis I
inosine-5'-phosphate biosynthesis I
superpathway of L-threonine biosynthesis
superpathway of β-D-glucuronide and D-glucuro...
pyrimidine deoxyribonucleotides de novo biosynthes...
6.37e-5
0.014
6.46e-4
1.09e-4
7.90e-5
8.77e-4
0.013
4.01e-4
8.61e-3
1.56e-3
0.022
3.67e-4
5.45e-5
5.14e-3
2.33e-3
6.92e-3
4.17e-7
5.85e-4
1.55e-3
5.62e-3
6.51e-5
0.021
2.11e-4
6.53e-5
3.06e-4
1.26e-4
1.45e-6
9.73e-4
0.027
0.020
q-value (corrected)

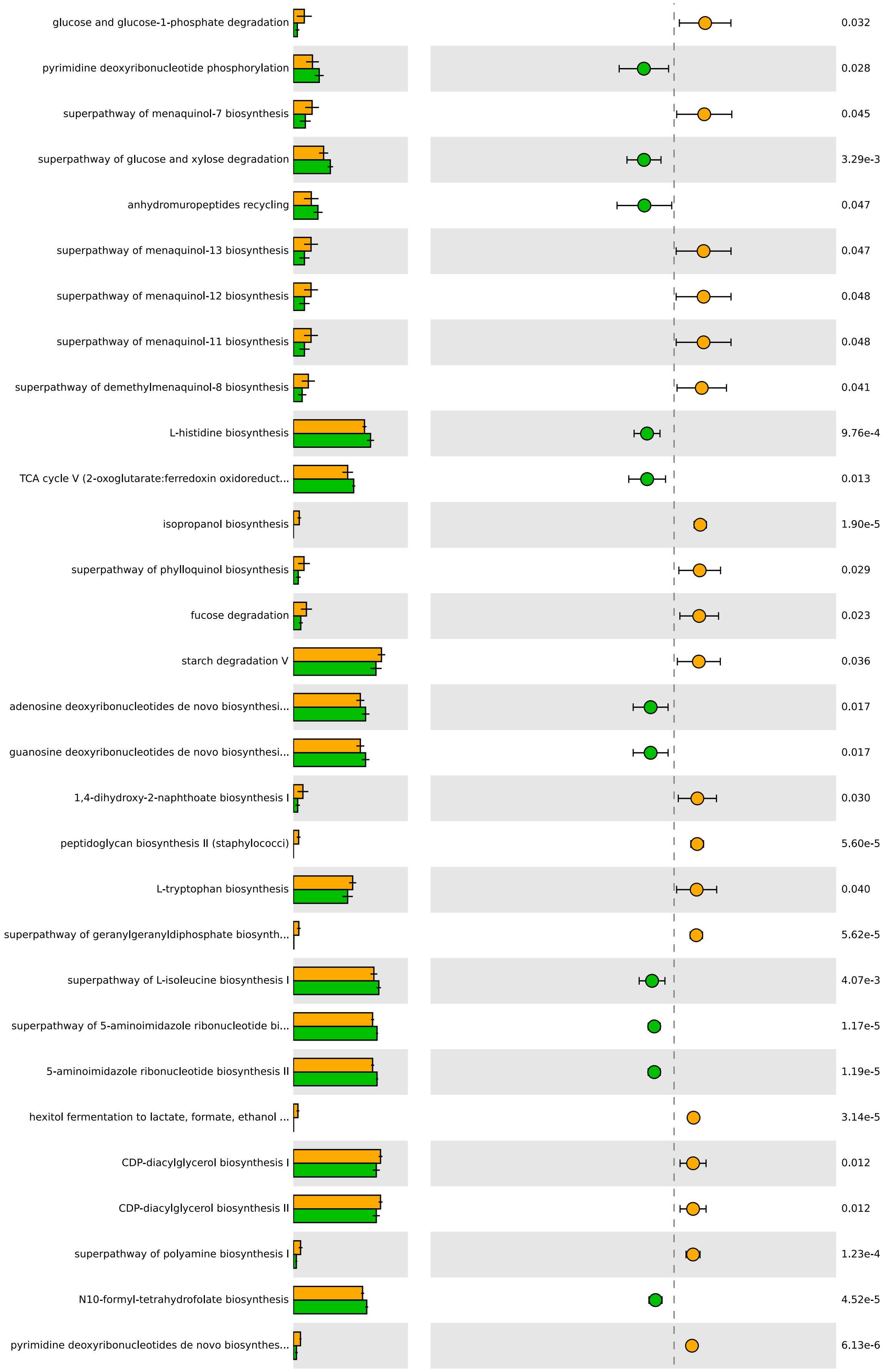
glucose and glucose-1-phosphate degradation
pyrimidine deoxyribonucleotide phosphorylation
superpathway of menaquinol-7 biosynthesis
superpathway of glucose and xylose degradation
anhydromuropeptides recycling
superpathway of menaquinol-13 biosynthesis
superpathway of menaquinol-12 biosynthesis
superpathway of menaquinol-11 biosynthesis
superpathway of demethylmenaquinol-8 biosynthesis
L-histidine biosynthesis
TCA cycle V (2-oxoglutarate:ferredoxin oxidoreduct...
isopropanol biosynthesis
superpathway of phylloquinol biosynthesis
fucose degradation
starch degradation V
adenosine deoxyribonucleotides de novo biosynthesi...
guanosine deoxyribonucleotides de novo biosynthesi...
1,4-dihydroxy-2-naphthoate biosynthesis I
peptidoglycan biosynthesis II (staphylococci)
L-tryptophan biosynthesis
superpathway of geranylgeranyldiphosphate biosynth...
superpathway of L-isoleucine biosynthesis I
superpathway of 5-aminoimidazole ribonucleotide bi...
5-aminoimidazole ribonucleotide biosynthesis II
hexitol fermentation to lactate, formate, ethanol ...
CDP-diacylglycerol biosynthesis I
CDP-diacylglycerol biosynthesis II
superpathway of polyamine biosynthesis I
N10-formyl-tetrahydrofolate biosynthesis
pyrimidine deoxyribonucleotides de novo biosynthes...
0.032
0.028
0.045
3.29e-3
0.047
0.047
0.048
0.048
0.041
9.76e-4
0.013
1.90e-5
0.029
0.023
0.036
0.017
0.017
0.030
5.60e-5
0.040
5.62e-5
4.07e-3
1.17e-5
1.19e-5
3.14e-5
0.012
0.012
1.23e-4
4.52e-5
6.13e-6

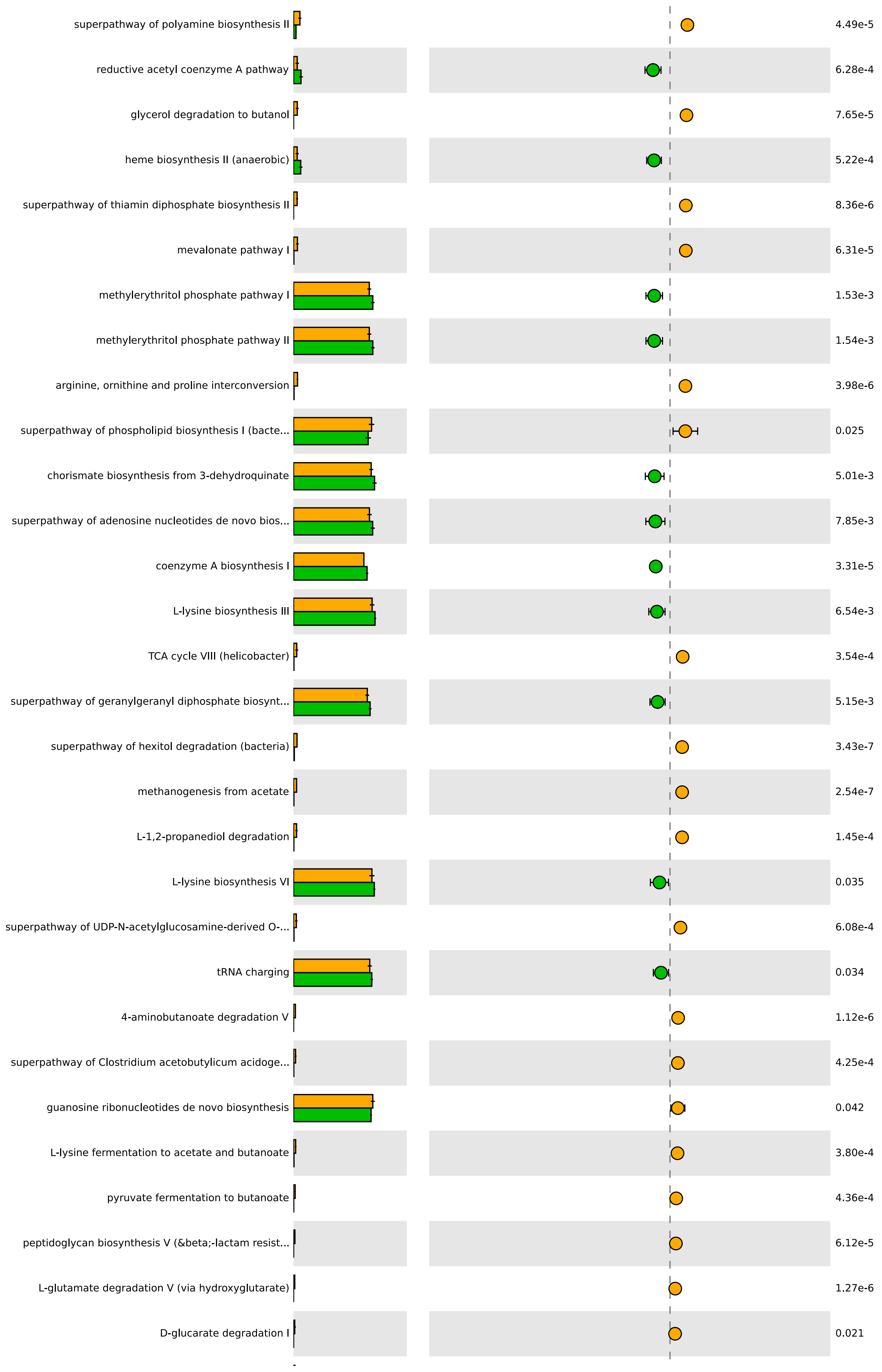
superpathway of polyamine biosynthesis II
reductive acetyl coenzyme A pathway
glycerol degradation to butanol
heme biosynthesis II (anaerobic)
superpathway of thiamin diphosphate biosynthesis II
mevalonate pathway I
methylerythritol phosphate pathway I
methylerythritol phosphate pathway II
arginine, ornithine and proline interconversion
superpathway of phospholipid biosynthesis I (bacte...
chorismate biosynthesis from 3-dehydroquinate
superpathway of adenosine nucleotides de novo bios...
coenzyme A biosynthesis I
L-lysine biosynthesis III
TCA cycle VIII (helicobacter)
superpathway of geranylgeranyl diphosphate biosynt...
superpathway of hexitol degradation (bacteria)
methanogenesis from acetate
L-1,2-propanediol degradation
L-lysine biosynthesis VI
superpathway of UDP-N-acetylglucosamine-derived O-...
tRNA charging
4-aminobutanoate degradation V
superpathway of Clostridium acetobutylicum acidoge...
guanosine ribonucleotides de novo biosynthesis
L-lysine fermentation to acetate and butanoate
pyruvate fermentation to butanoate
peptidoglycan biosynthesis V (β-lactam resist...
L-glutamate degradation V (via hydroxyglutarate)
D-glucarate degradation I
4.49e-5
6.28e-4
7.65e-5
5.22e-4
8.36e-6
6.31e-5
1.53e-3
1.54e-3
3.98e-6
0.025
5.01e-3
7.85e-3
3.31e-5
6.54e-3
3.54e-4
5.15e-3
3.43e-7
2.54e-7
1.45e-4
0.035
6.08e-4
0.034
1.12e-6
4.25e-4
0.042
3.80e-4
4.36e-4
6.12e-5
1.27e-6
0.021

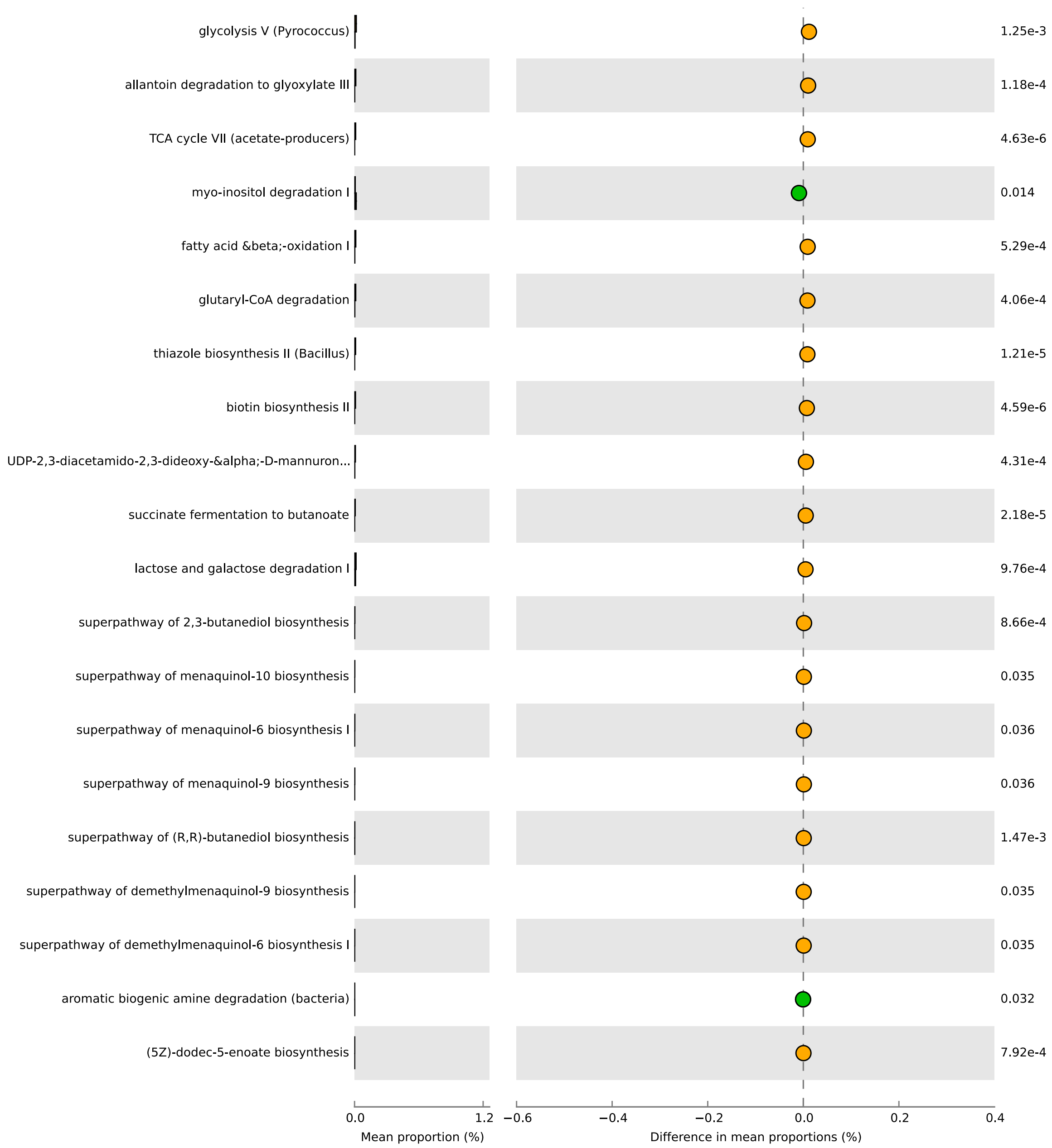

Figure S8. PICRUSt2 results indicating differential predicted functional pathways between the bean-free and the bean-containing diet groups in the female cohort: **(a)** 152 pathways in 0% vs 17.5% bean; **(b)** 183 pathways in 0% vs 35% bean; **(c)** 198 pathways in the 0% vs 70% bean. The bean-free diet (*0%*) is indicated in beige, while the bean-based diet samples (*17.5%; 35%*, and *70%*)—in green. Extended error bar plot indicating the mean proportion of pathways assigned to each group, difference between them, and corrected *p*-value (*q*-value) of each.

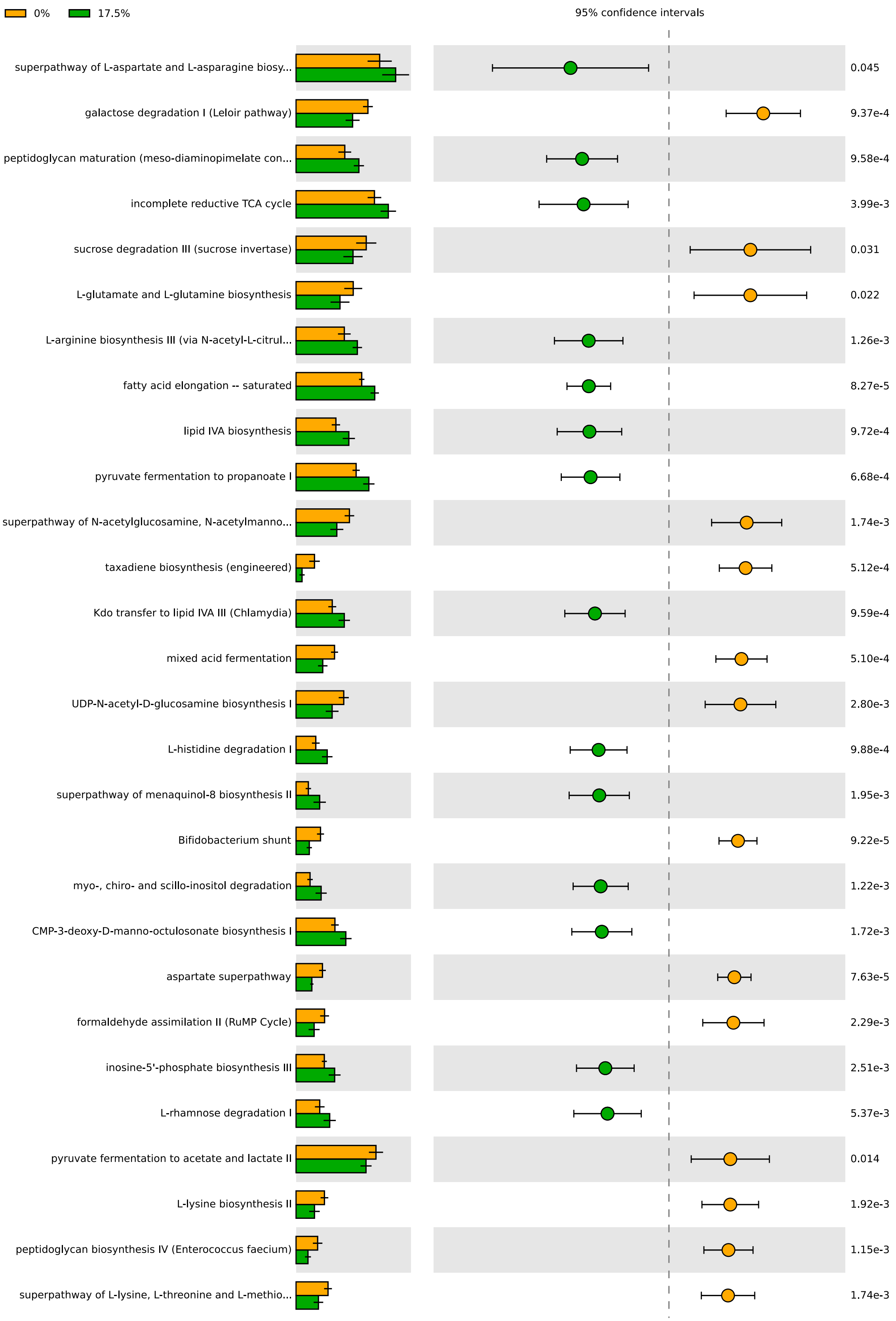
0%
17.5%
95% confidence intervals
superpathway of L-aspartate and L-asparagine biosy...
0.045
galactose degradation I (Leloir pathway)
9.37e-4
peptidoglycan maturation (meso-diaminopimelate con...
9.58e-4
incomplete reductive TCA cycle
3.99e-3
sucrose degradation III (sucrose invertase)
0.031
L-glutamate and L-glutamine biosynthesis
0.022
L-arginine biosynthesis III (via N-acetyl-L-citrul...
1.26e-3
fatty acid elongation -- saturated
8.27e-5
lipid IVA biosynthesis
9.72e-4
pyruvate fermentation to propanoate I
6.68e-4
superpathway of N-acetylglucosamine, N-acetylmanno...
1.74e-3
taxadiene biosynthesis (engineered)
5.12e-4
Kdo transfer to lipid IVA III (Chlamydia)
9.59e-4
mixed acid fermentation
5.10e-4
UDP-N-acetyl-D-glucosamine biosynthesis I
2.80e-3
L-histidine degradation I
9.88e-4
superpathway of menaquinol-8 biosynthesis II
1.95e-3
Bifidobacterium shunt
9.22e-5
myo-, chiro- and scillo-inositol degradation
1.22e-3
CMP-3-deoxy-D-manno-octulosonate biosynthesis I
1.72e-3
aspartate superpathway
7.63e-5
formaldehyde assimilation II (RuMP Cycle)
2.29e-3
inosine-5'-phosphate biosynthesis III
2.51e-3
L-rhamnose degradation I
5.37e-3
pyruvate fermentation to acetate and lactate II
0.014
L-lysine biosynthesis II
1.92e-3
peptidoglycan biosynthesis IV (Enterococcus faecium)
1.15e-3
superpathway of L-lysine, L-threonine and L-methio...
1.74e-3

a.

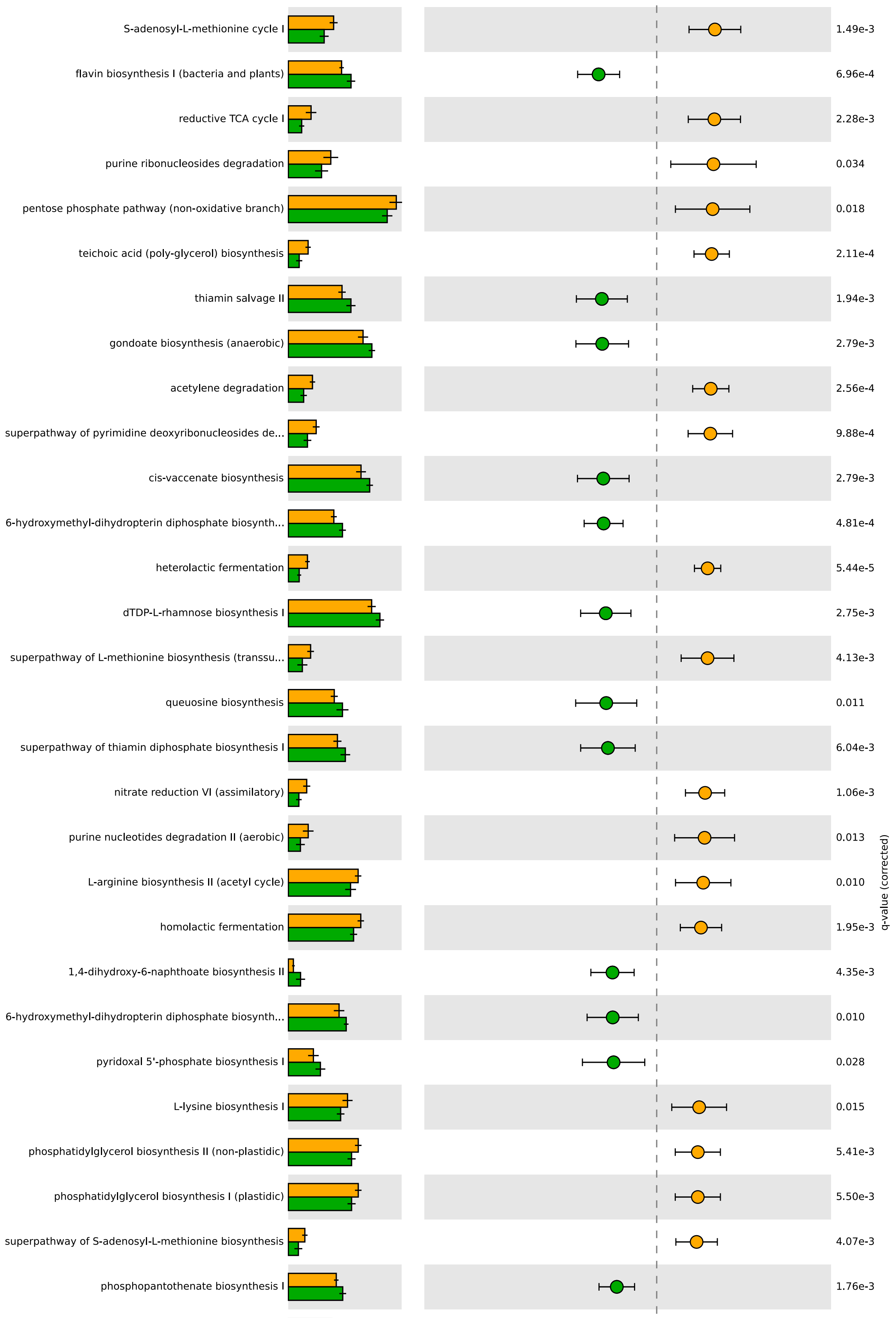
S-adenosyl-L-methionine cycle I
flavin biosynthesis I (bacteria and plants)
reductive TCA cycle I
purine ribonucleosides degradation
pentose phosphate pathway (non-oxidative branch)
teichoic acid (poly-glycerol) biosynthesis
thiamin salvage II
gondoate biosynthesis (anaerobic)
acetylene degradation
superpathway of pyrimidine deoxyribonucleosides de...
cis-vaccenate biosynthesis
6-hydroxymethyl-dihydropterin diphosphate biosynth...
heterolactic fermentation
dTDP-L-rhamnose biosynthesis I
superpathway of L-methionine biosynthesis (transsu...
queuosine biosynthesis
superpathway of thiamin diphosphate biosynthesis I
nitrate reduction VI (assimilatory)
purine nucleotides degradation II (aerobic)
L-arginine biosynthesis II (acetyl cycle)
homolactic fermentation
1,4-dihydroxy-6-naphthoate biosynthesis II
6-hydroxymethyl-dihydropterin diphosphate biosynth...
pyridoxal 5'-phosphate biosynthesis I
L-lysine biosynthesis I
phosphatidylglycerol biosynthesis II (non-plastidic)
phosphatidylglycerol biosynthesis I (plastidic)
superpathway of S-adenosyl-L-methionine biosynthesis
phosphopantothenate biosynthesis I
1.49e-3
6.96e-4
2.28e-3
0.034
0.018
2.11e-4
1.94e-3
2.79e-3
2.56e-4
9.88e-4
2.79e-3
4.81e-4
5.44e-5
2.75e-3
4.13e-3
0.011
6.04e-3
1.06e-3
0.013
0.010
1.95e-3
4.35e-3
0.010
0.028
0.015
5.41e-3
5.50e-3
4.07e-3
1.76e-3
q-value (corrected)

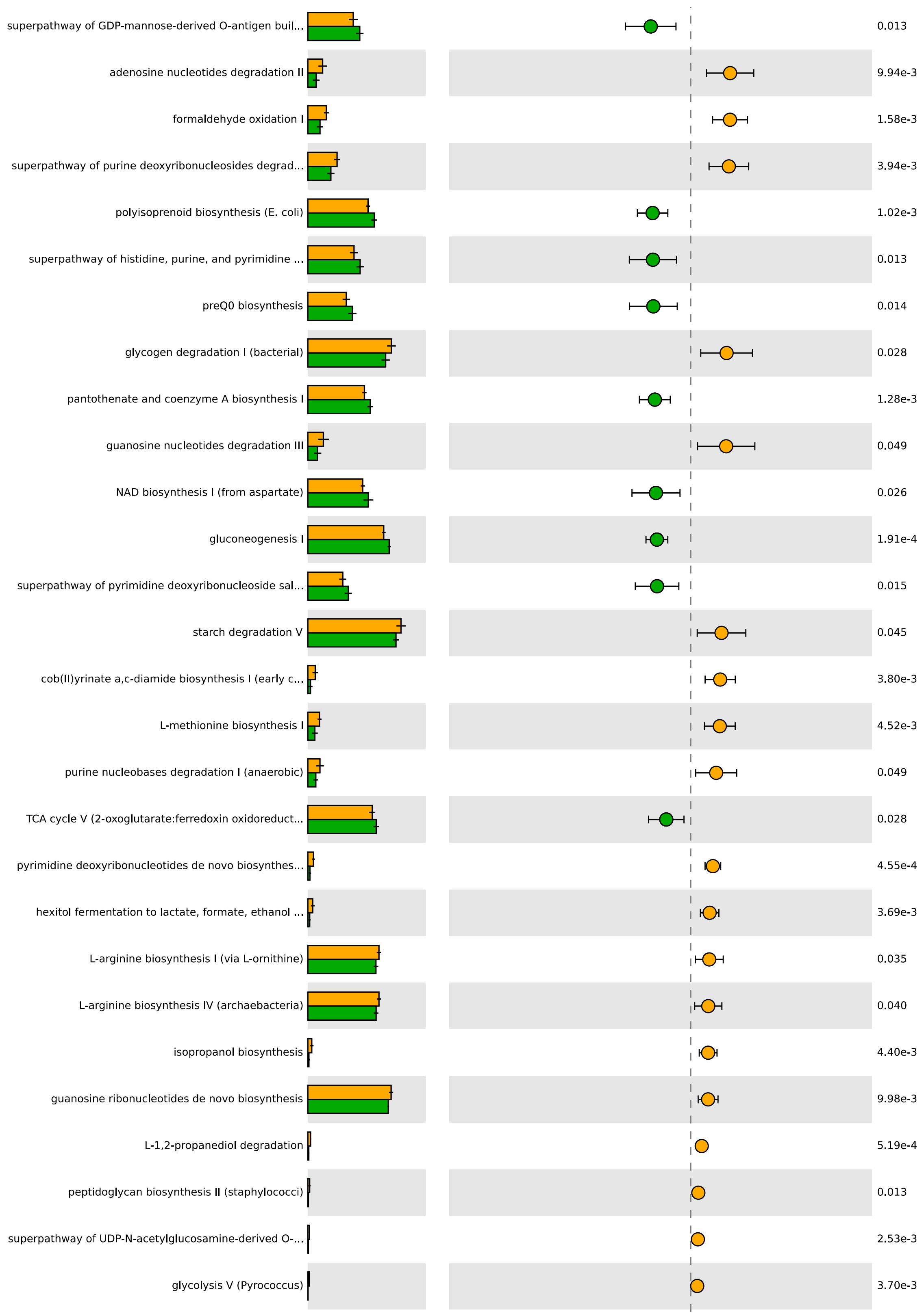
superpathway of GDP-mannose-derived O-antigen buil...
0.013
adenosine nucleotides degradation II
9.94e-3
formaldehyde oxidation I
1.58e-3
superpathway of purine deoxyribonucleosides degrad...
3.94e-3
polyisoprenoid biosynthesis (E. coli)
1.02e-3
superpathway of histidine, purine, and pyrimidine ...
0.013
preQ0 biosynthesis
0.014
glycogen degradation I (bacterial)
0.028
pantothenate and coenzyme A biosynthesis I
1.28e-3
guanosine nucleotides degradation III
0.049
NAD biosynthesis I (from aspartate)
0.026
gluconeogenesis I
1.91e-4
superpathway of pyrimidine deoxyribonucleoside sal...
0.015
starch degradation V
0.045
cob(II)yrinate a,c-diamide biosynthesis I (early c...
3.80e-3
L-methionine biosynthesis I
4.52e-3
purine nucleobases degradation I (anaerobic)
0.049
TCA cycle V (2-oxoglutarate:ferredoxin oxidoreduct...
0.028
pyrimidine deoxyribonucleotides de novo biosynthes...
4.55e-4
hexitol fermentation to lactate, formate, ethanol ...
3.69e-3
L-arginine biosynthesis I (via L-ornithine)
0.035
L-arginine biosynthesis IV (archaebacteria)
0.040
isopropanol biosynthesis
4.40e-3
guanosine ribonucleotides de novo biosynthesis
9.98e-3
L-1,2-propanediol degradation
5.19e-4
peptidoglycan biosynthesis II (staphylococci)
0.013
superpathway of UDP-N-acetylglucosamine-derived O-...
2.53e-3
glycolysis V (Pyrococcus)
3.70e-3

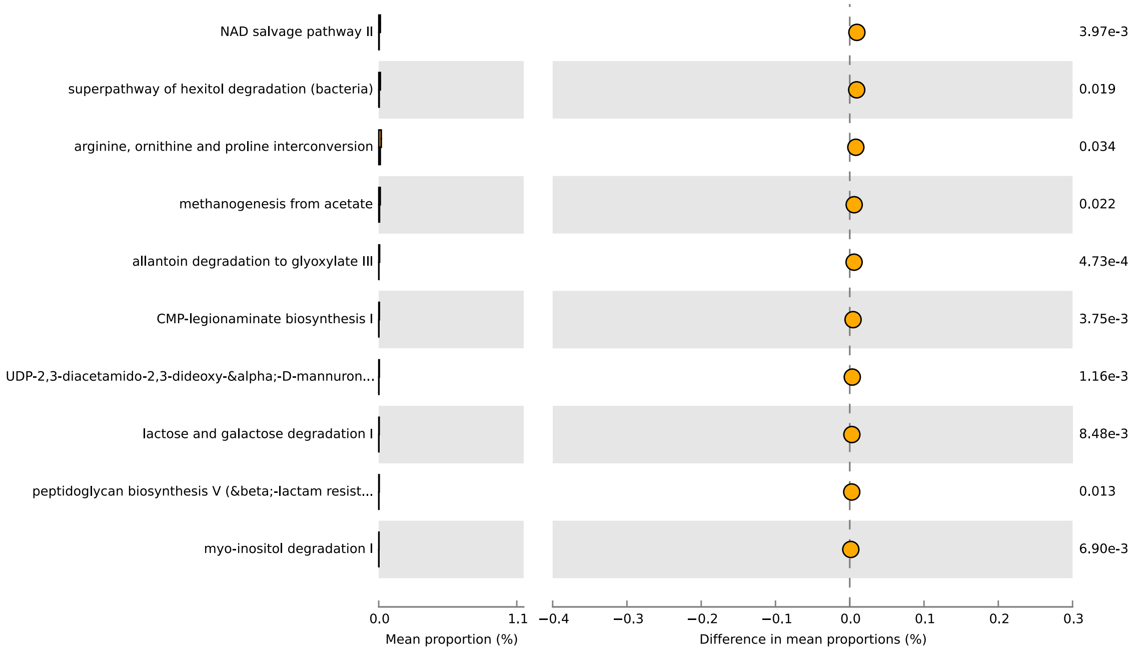

NAD salvage pathway II
superpathway of hexitol degradation (bacteria)
arginine, ornithine and proline interconversion
methanogenesis from acetate
allantoin degradation to glyoxylate III
CMP-legionaminate biosynthesis I
UDP-2,3-diacetamido-2,3-dideoxy-α-D-mannuron...
lactose and galactose degradation I
peptidoglycan biosynthesis V (β-lactam resist...
myo-inositol degradation I
3.97e-3
0.019
0.034
0.022
4.73e-4
3.75e-3
1.16e-3
8.48e-3
0.013
6.90e-3
0.0
1.1
Mean proportion (%)
−0.4
−0.3
−0.2
−0.1
0.0
0.1
0.2
0.3
Difference in mean proportions (%)

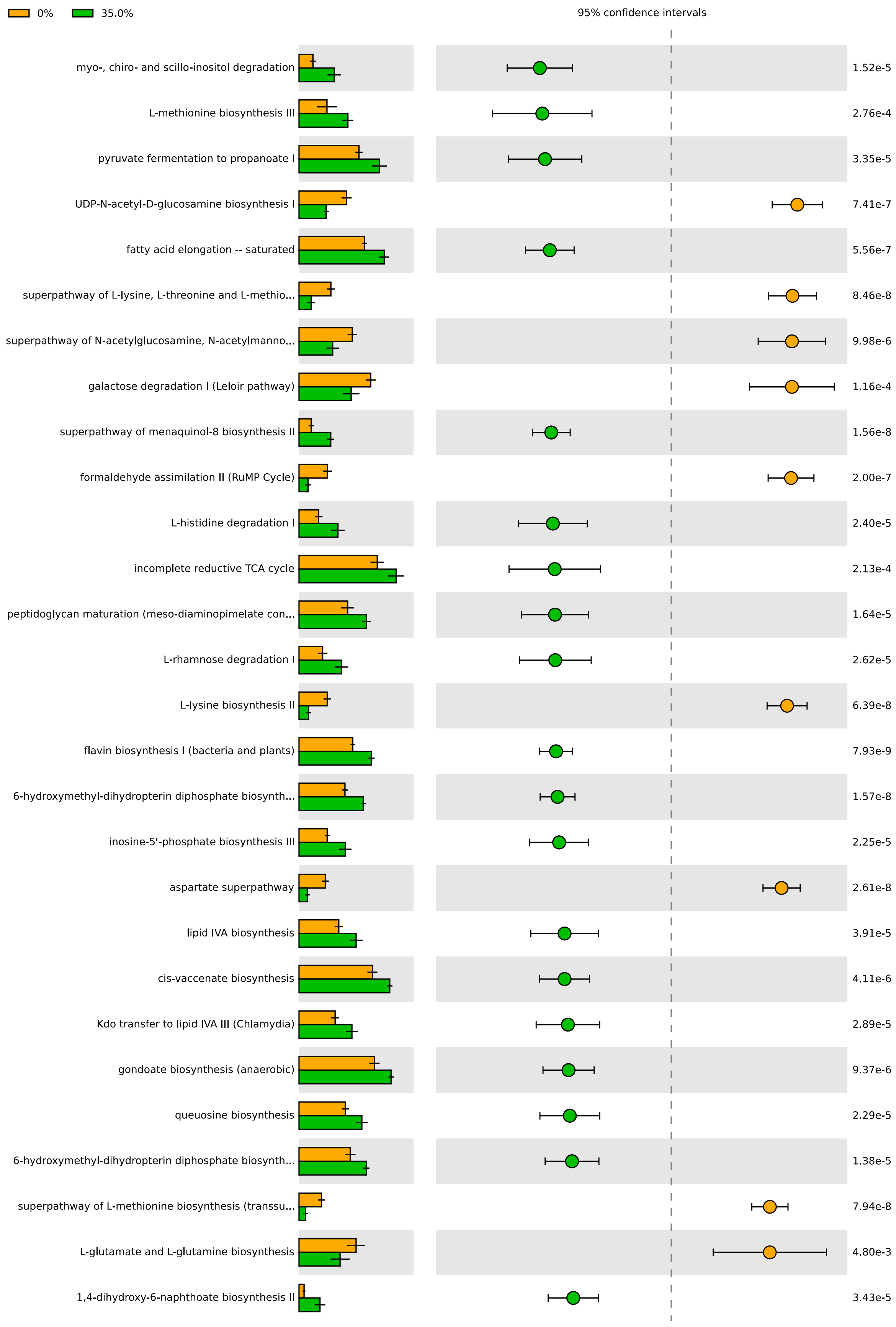
0%
35.0%
95% confidence intervals
myo-, chiro- and scillo-inositol degradation
L-methionine biosynthesis III
pyruvate fermentation to propanoate I
UDP-N-acetyl-D-glucosamine biosynthesis I
fatty acid elongation -- saturated
superpathway of L-lysine, L-threonine and L-methio...
superpathway of N-acetylglucosamine, N-acetylmanno...
galactose degradation I (Leloir pathway)
superpathway of menaquinol-8 biosynthesis II
formaldehyde assimilation II (RuMP Cycle)
L-histidine degradation I
incomplete reductive TCA cycle
peptidoglycan maturation (meso-diaminopimelate con...
L-rhamnose degradation I
L-lysine biosynthesis II
flavin biosynthesis I (bacteria and plants)
6-hydroxymethyl-dihydropterin diphosphate biosynth...
inosine-5'-phosphate biosynthesis III
aspartate superpathway
lipid IVA biosynthesis
cis-vaccenate biosynthesis
Kdo transfer to lipid IVA III (Chlamydia)
gondoate biosynthesis (anaerobic)
queuosine biosynthesis
6-hydroxymethyl-dihydropterin diphosphate biosynth...
superpathway of L-methionine biosynthesis (transsu...
L-glutamate and L-glutamine biosynthesis
1,4-dihydroxy-6-naphthoate biosynthesis II
1.52e-5
2.76e-4
3.35e-5
7.41e-7
5.56e-7
8.46e-8
9.98e-6
1.16e-4
1.56e-8
2.00e-7
2.40e-5
2.13e-4
1.64e-5
2.62e-5
6.39e-8
7.93e-9
1.57e-8
2.25e-5
2.61e-8
3.91e-5
4.11e-6
2.89e-5
9.37e-6
2.29e-5
1.38e-5
7.94e-8
4.80e-3
3.43e-5
b.

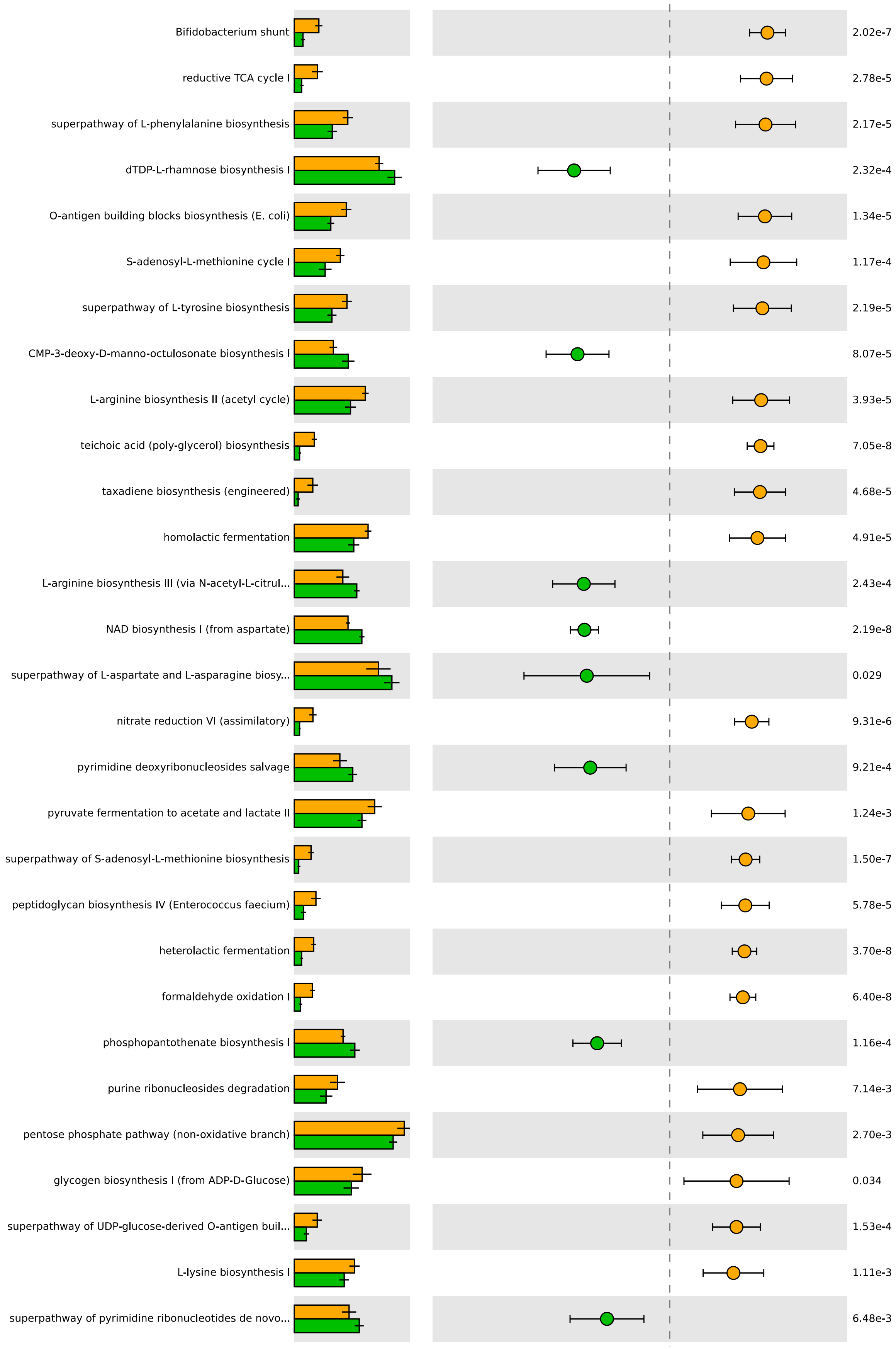
Bifidobacterium shunt
2.02e-7
reductive TCA cycle I
2.78e-5
superpathway of L-phenylalanine biosynthesis
2.17e-5
dTDP-L-rhamnose biosynthesis I
2.32e-4
O-antigen building blocks biosynthesis (E. coli)
1.34e-5
S-adenosyl-L-methionine cycle I
1.17e-4
superpathway of L-tyrosine biosynthesis
2.19e-5
CMP-3-deoxy-D-manno-octulosonate biosynthesis I
8.07e-5
L-arginine biosynthesis II (acetyl cycle)
3.93e-5
teichoic acid (poly-glycerol) biosynthesis
7.05e-8
taxadiene biosynthesis (engineered)
4.68e-5
homolactic fermentation
4.91e-5
L-arginine biosynthesis III (via N-acetyl-L-citrul...
2.43e-4
NAD biosynthesis I (from aspartate)
2.19e-8
superpathway of L-aspartate and L-asparagine biosy...
0.029
nitrate reduction VI (assimilatory)
9.31e-6
pyrimidine deoxyribonucleosides salvage
9.21e-4
pyruvate fermentation to acetate and lactate II
1.24e-3
superpathway of S-adenosyl-L-methionine biosynthesis
1.50e-7
peptidoglycan biosynthesis IV (Enterococcus faecium)
5.78e-5
heterolactic fermentation
3.70e-8
formaldehyde oxidation I
6.40e-8
phosphopantothenate biosynthesis I
1.16e-4
purine ribonucleosides degradation
7.14e-3
pentose phosphate pathway (non-oxidative branch)
2.70e-3
glycogen biosynthesis I (from ADP-D-Glucose)
0.034
superpathway of UDP-glucose-derived O-antigen buil...
1.53e-4
L-lysine biosynthesis I
1.11e-3
superpathway of pyrimidine ribonucleotides de novo...
6.48e-3

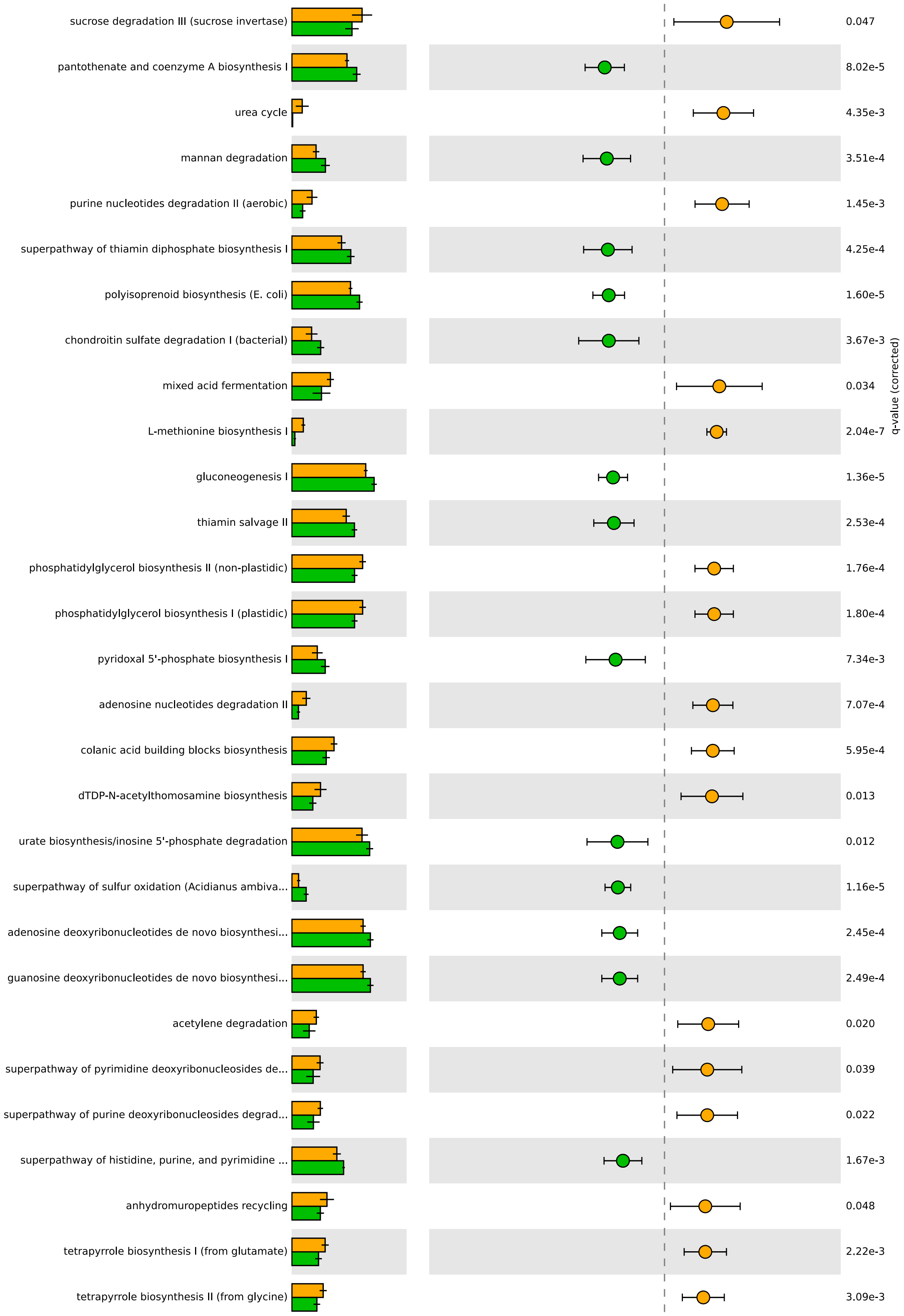

sucrose degradation III (sucrose invertase)
pantothenate and coenzyme A biosynthesis I
urea cycle
mannan degradation
purine nucleotides degradation II (aerobic)
superpathway of thiamin diphosphate biosynthesis I
polyisoprenoid biosynthesis (E. coli)
chondroitin sulfate degradation I (bacterial)
mixed acid fermentation
L-methionine biosynthesis I
gluconeogenesis I
thiamin salvage II
phosphatidylglycerol biosynthesis II (non-plastidic)
phosphatidylglycerol biosynthesis I (plastidic)
pyridoxal 5'-phosphate biosynthesis I
adenosine nucleotides degradation II
colanic acid building blocks biosynthesis
dTDP-N-acetylthomosamine biosynthesis
urate biosynthesis/inosine 5'-phosphate degradation
superpathway of sulfur oxidation (Acidianus ambiva...
adenosine deoxyribonucleotides de novo biosynthesi...
guanosine deoxyribonucleotides de novo biosynthesi...
acetylene degradation
superpathway of pyrimidine deoxyribonucleosides de...
superpathway of purine deoxyribonucleosides degrad...
superpathway of histidine, purine, and pyrimidine ...
anhydromuropeptides recycling
tetrapyrrole biosynthesis I (from glutamate)
tetrapyrrole biosynthesis II (from glycine)
0.047
8.02e-5
4.35e-3
3.51e-4
1.45e-3
4.25e-4
1.60e-5
3.67e-3
0.034
2.04e-7
1.36e-5
2.53e-4
1.76e-4
1.80e-4
7.34e-3
7.07e-4
5.95e-4
0.013
0.012
1.16e-5
2.45e-4
2.49e-4
0.020
0.039
0.022
1.67e-3
0.048
2.22e-3
3.09e-3
q-value (corrected)

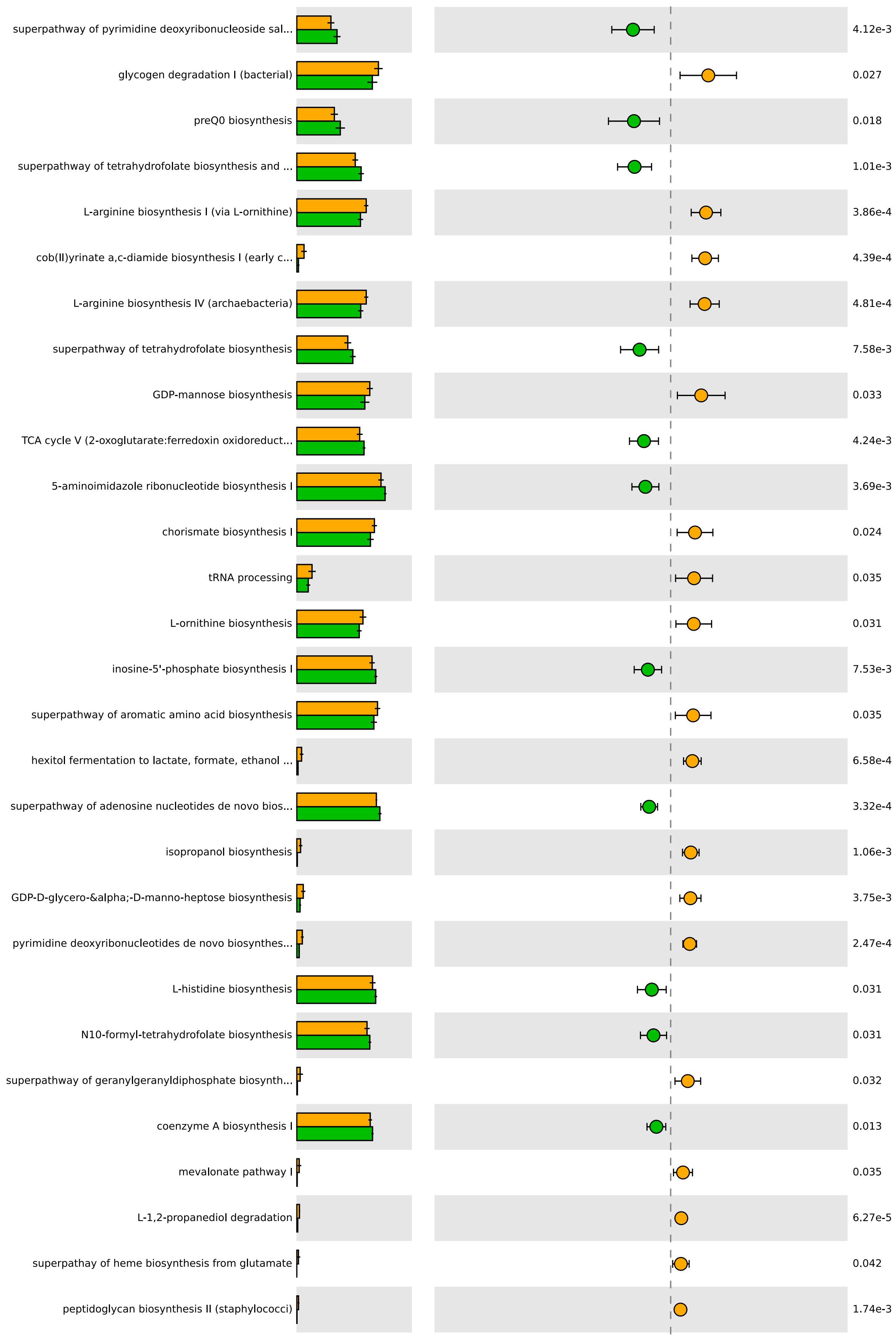
superpathway of pyrimidine deoxyribonucleoside sal...
4.12e-3
glycogen degradation I (bacterial)
0.027
preQ0 biosynthesis
0.018
superpathway of tetrahydrofolate biosynthesis and ...
1.01e-3
L-arginine biosynthesis I (via L-ornithine)
3.86e-4
cob(II)yrinate a,c-diamide biosynthesis I (early c...
4.39e-4
L-arginine biosynthesis IV (archaebacteria)
4.81e-4
superpathway of tetrahydrofolate biosynthesis
7.58e-3
GDP-mannose biosynthesis
0.033
TCA cycle V (2-oxoglutarate:ferredoxin oxidoreduct...
4.24e-3
5-aminoimidazole ribonucleotide biosynthesis I
3.69e-3
chorismate biosynthesis I
0.024
tRNA processing
0.035
L-ornithine biosynthesis
0.031
inosine-5'-phosphate biosynthesis I
7.53e-3
superpathway of aromatic amino acid biosynthesis
0.035
hexitol fermentation to lactate, formate, ethanol ...
6.58e-4
superpathway of adenosine nucleotides de novo bios...
3.32e-4
isopropanol biosynthesis
1.06e-3
GDP-D-glycero-α-D-manno-heptose biosynthesis
3.75e-3
pyrimidine deoxyribonucleotides de novo biosynthes...
2.47e-4
L-histidine biosynthesis
0.031
N10-formyl-tetrahydrofolate biosynthesis
0.031
superpathway of geranylgeranyldiphosphate biosynth...
0.032
coenzyme A biosynthesis I
0.013
mevalonate pathway I
0.035
L-1,2-propanediol degradation
6.27e-5
superpathay of heme biosynthesis from glutamate
0.042
peptidoglycan biosynthesis II (staphylococci)
1.74e-3

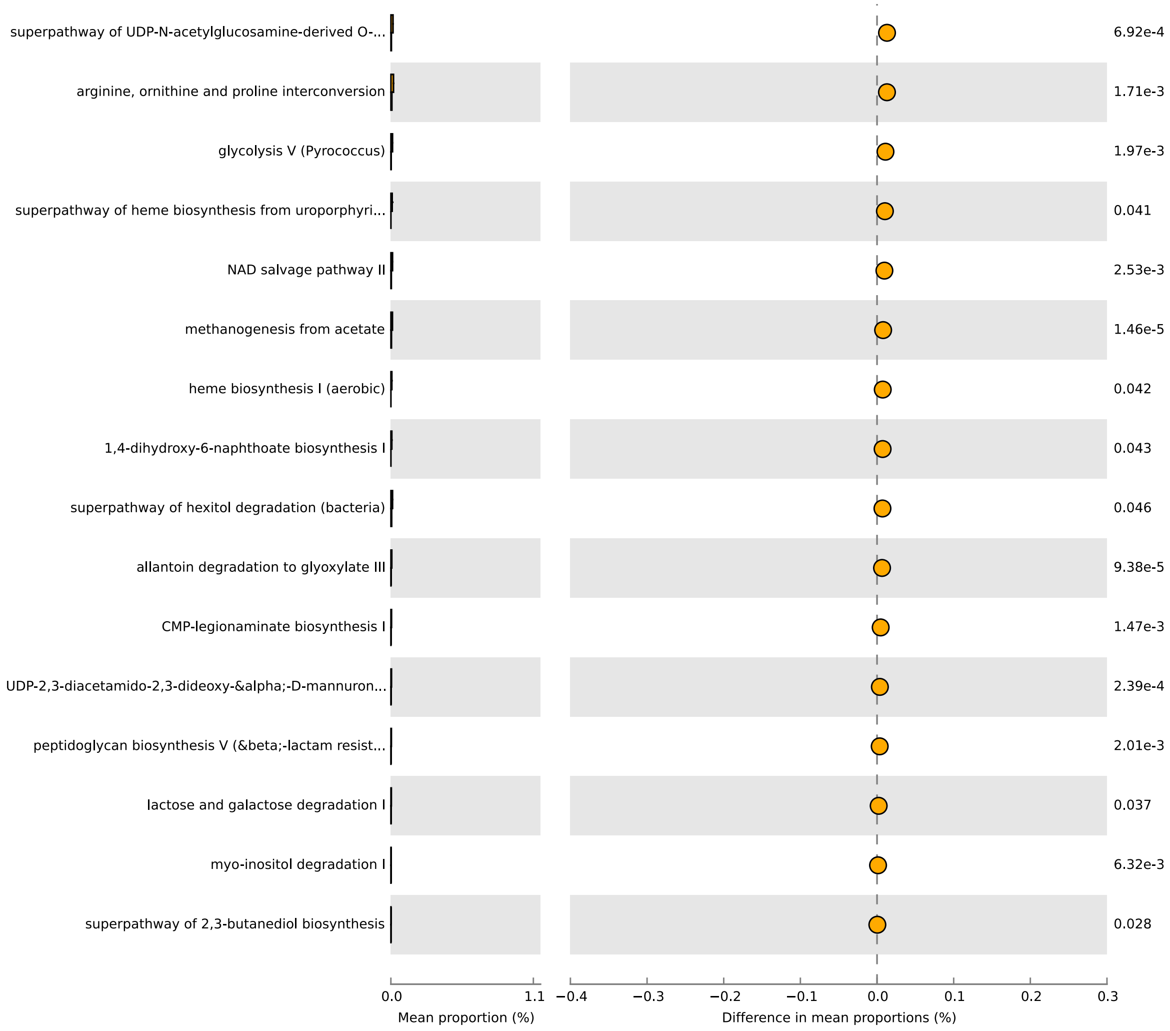
superpathway of UDP-N-acetylglucosamine-derived O-...
6.92e-4
arginine, ornithine and proline interconversion
1.71e-3
glycolysis V (Pyrococcus)
1.97e-3
superpathway of heme biosynthesis from uroporphyri...
0.041
NAD salvage pathway II
2.53e-3
methanogenesis from acetate
1.46e-5
heme biosynthesis I (aerobic)
0.042
1,4-dihydroxy-6-naphthoate biosynthesis I
0.043
superpathway of hexitol degradation (bacteria)
0.046
allantoin degradation to glyoxylate III
9.38e-5
CMP-legionaminate biosynthesis I
1.47e-3
UDP-2,3-diacetamido-2,3-dideoxy-α-D-mannuron...
2.39e-4
peptidoglycan biosynthesis V (β-lactam resist...
2.01e-3
lactose and galactose degradation I
0.037
myo-inositol degradation I
6.32e-3
superpathway of 2,3-butanediol biosynthesis
0.028
0.0
1.1
Mean proportion (%)
−0.4
−0.3
−0.2
−0.1
0.0
0.1
0.2
0.3
Difference in mean proportions (%)

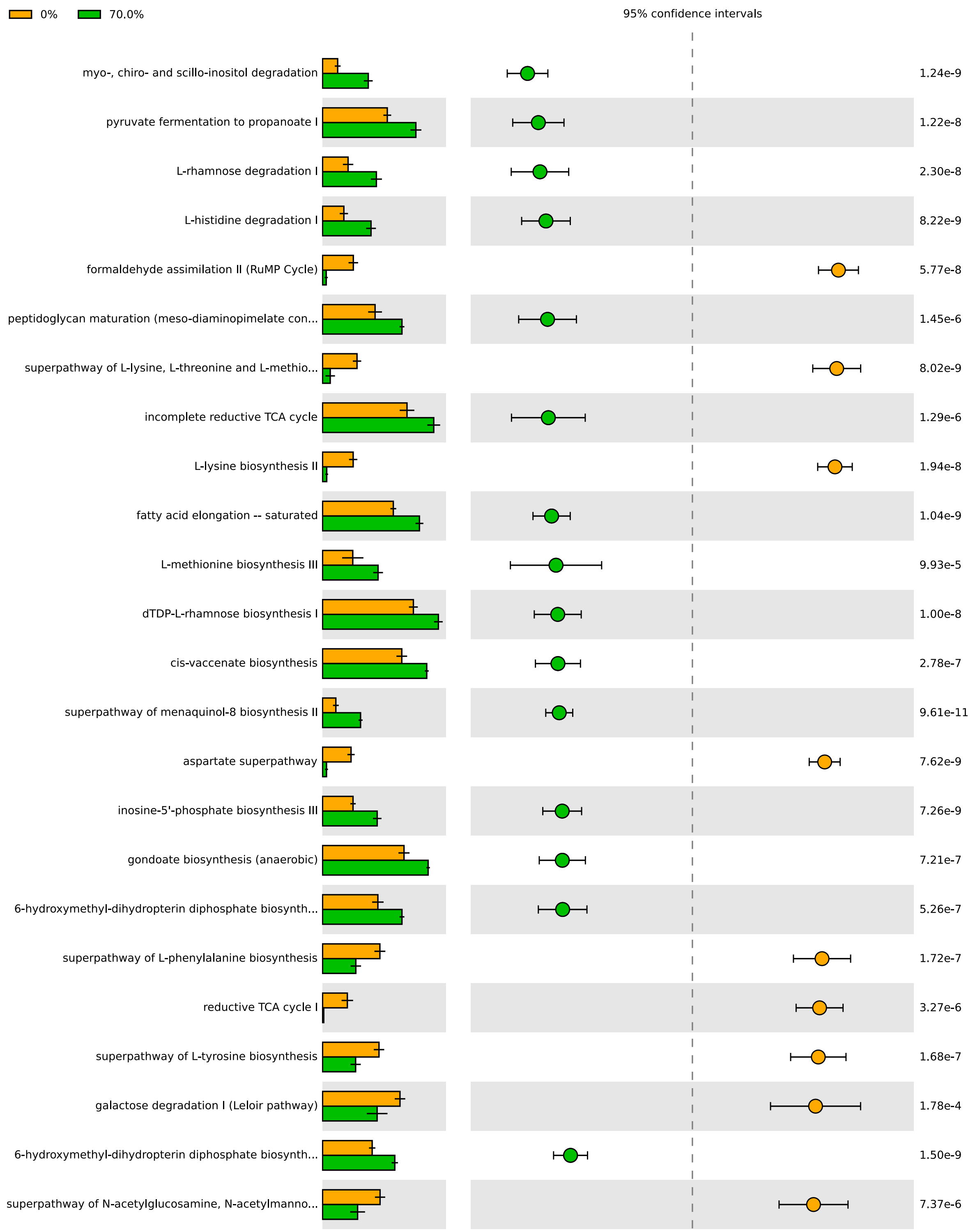
0%
70.0%
95% confidence intervals
myo-, chiro- and scillo-inositol degradation
pyruvate fermentation to propanoate I
L-rhamnose degradation I
L-histidine degradation I
formaldehyde assimilation II (RuMP Cycle)
peptidoglycan maturation (meso-diaminopimelate con...
superpathway of L-lysine, L-threonine and L-methio...
incomplete reductive TCA cycle
L-lysine biosynthesis II
fatty acid elongation -- saturated
L-methionine biosynthesis III
dTDP-L-rhamnose biosynthesis I
cis-vaccenate biosynthesis
superpathway of menaquinol-8 biosynthesis II
aspartate superpathway
inosine-5'-phosphate biosynthesis III
gondoate biosynthesis (anaerobic)
6-hydroxymethyl-dihydropterin diphosphate biosynth...
superpathway of L-phenylalanine biosynthesis
reductive TCA cycle I
superpathway of L-tyrosine biosynthesis
galactose degradation I (Leloir pathway)
6-hydroxymethyl-dihydropterin diphosphate biosynth...
superpathway of N-acetylglucosamine, N-acetylmanno...
1.24e-9
1.22e-8
2.30e-8
8.22e-9
5.77e-8
1.45e-6
8.02e-9
1.29e-6
1.94e-8
1.04e-9
9.93e-5
1.00e-8
2.78e-7
9.61e-11
7.62e-9
7.26e-9
7.21e-7
5.26e-7
1.72e-7
3.27e-6
1.68e-7
1.78e-4
1.50e-9
7.37e-6

c.

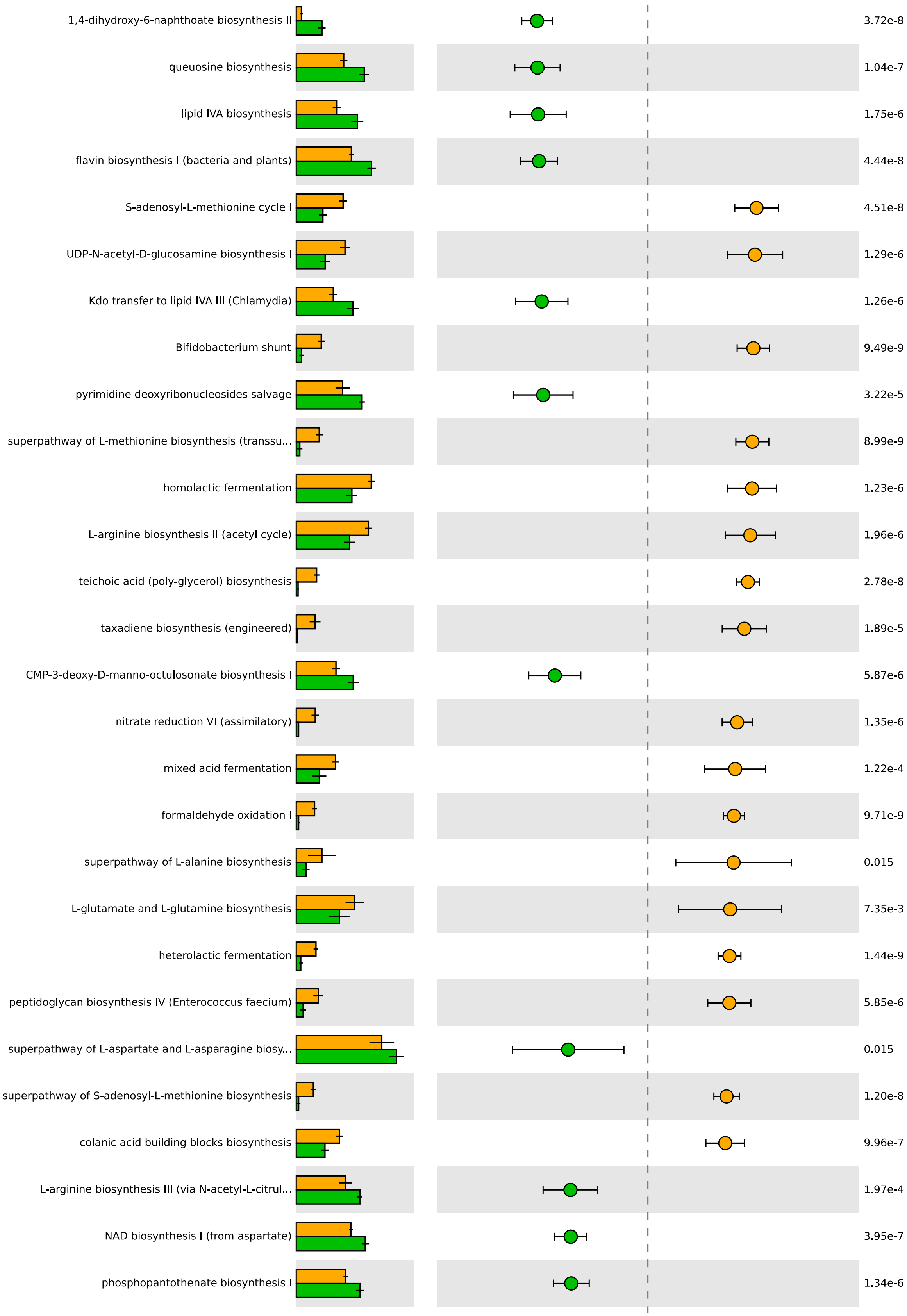
1,4-dihydroxy-6-naphthoate biosynthesis II 3.72e-8
queuosine biosynthesis 1.04e-7
lipid IVA biosynthesis 1.75e-6
flavin biosynthesis I (bacteria and plants) 4.44e-8
S-adenosyl-L-methionine cycle I 4.51e-8
UDP-N-acetyl-D-glucosamine biosynthesis I 1.29e-6
Kdo transfer to lipid IVA III (Chlamydia) 1.26e-6
Bifidobacterium shunt 9.49e-9
pyrimidine deoxyribonucleosides salvage 3.22e-5
superpathway of L-methionine biosynthesis (transsu... 8.99e-9
homolactic fermentation 1.23e-6
L-arginine biosynthesis II (acetyl cycle) 1.96e-6
teichoic acid (poly-glycerol) biosynthesis 2.78e-8
taxadiene biosynthesis (engineered) 1.89e-5
CMP-3-deoxy-D-manno-octulosonate biosynthesis I 5.87e-6
nitrate reduction VI (assimilatory) 1.35e-6
mixed acid fermentation 1.22e-4
formaldehyde oxidation I 9.71e-9
superpathway of L-alanine biosynthesis 0.015
L-glutamate and L-glutamine biosynthesis 7.35e-3
heterolactic fermentation 1.44e-9
peptidoglycan biosynthesis IV (Enterococcus faecium) 5.85e-6
superpathway of L-aspartate and L-asparagine biosy... 0.015
superpathway of S-adenosyl-L-methionine biosynthesis 1.20e-8
colanic acid building blocks biosynthesis 9.96e-7
L-arginine biosynthesis III (via N-acetyl-L-citrul... 1.97e-4
NAD biosynthesis I (from aspartate) 3.95e-7
phosphopantothenate biosynthesis I 1.34e-6

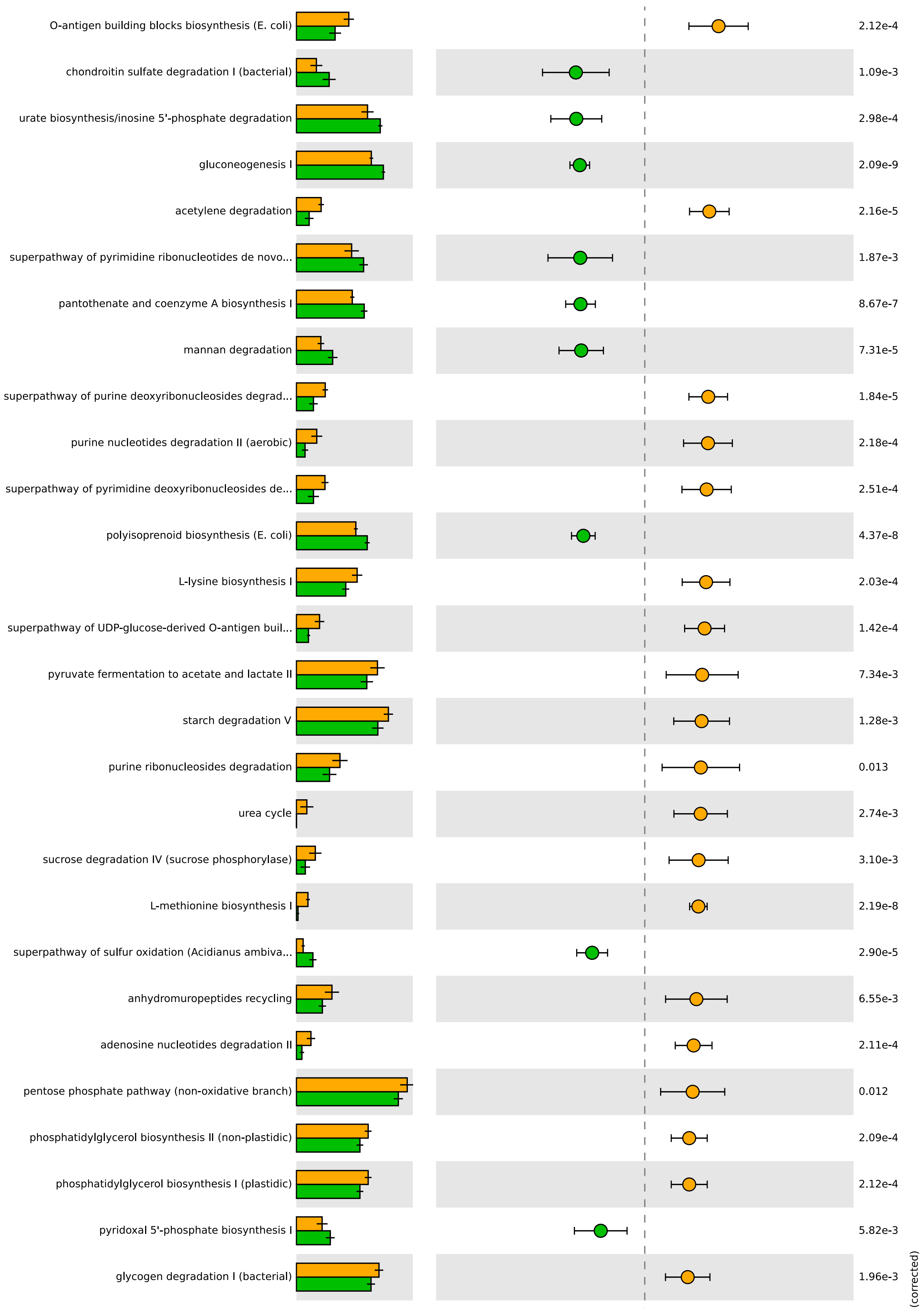
O-antigen building blocks biosynthesis (E. coli)
chondroitin sulfate degradation I (bacterial)
urate biosynthesis/inosine 5'-phosphate degradation
gluconeogenesis I
acetylene degradation
superpathway of pyrimidine ribonucleotides de novo...
pantothenate and coenzyme A biosynthesis I
mannan degradation
superpathway of purine deoxyribonucleosides degrad...
purine nucleotides degradation II (aerobic)
superpathway of pyrimidine deoxyribonucleosides de...
polyisoprenoid biosynthesis (E. coli)
L-lysine biosynthesis I
superpathway of UDP-glucose-derived O-antigen buil...
pyruvate fermentation to acetate and lactate II
starch degradation V
purine ribonucleosides degradation
urea cycle
sucrose degradation IV (sucrose phosphorylase)
L-methionine biosynthesis I
superpathway of sulfur oxidation (Acidianus ambiva...
anhydromuropeptides recycling
adenosine nucleotides degradation II
pentose phosphate pathway (non-oxidative branch)
phosphatidylglycerol biosynthesis II (non-plastidic)
phosphatidylglycerol biosynthesis I (plastidic)
pyridoxal 5'-phosphate biosynthesis I
glycogen degradation I (bacterial)
2.12e-4
1.09e-3
2.98e-4
2.09e-9
2.16e-5
1.87e-3
8.67e-7
7.31e-5
1.84e-5
2.18e-4
2.51e-4
4.37e-8
2.03e-4
1.42e-4
7.34e-3
1.28e-3
0.013
2.74e-3
3.10e-3
2.19e-8
2.90e-5
6.55e-3
2.11e-4
0.012
2.09e-4
2.12e-4
5.82e-3
1.96e-3
(corrected)

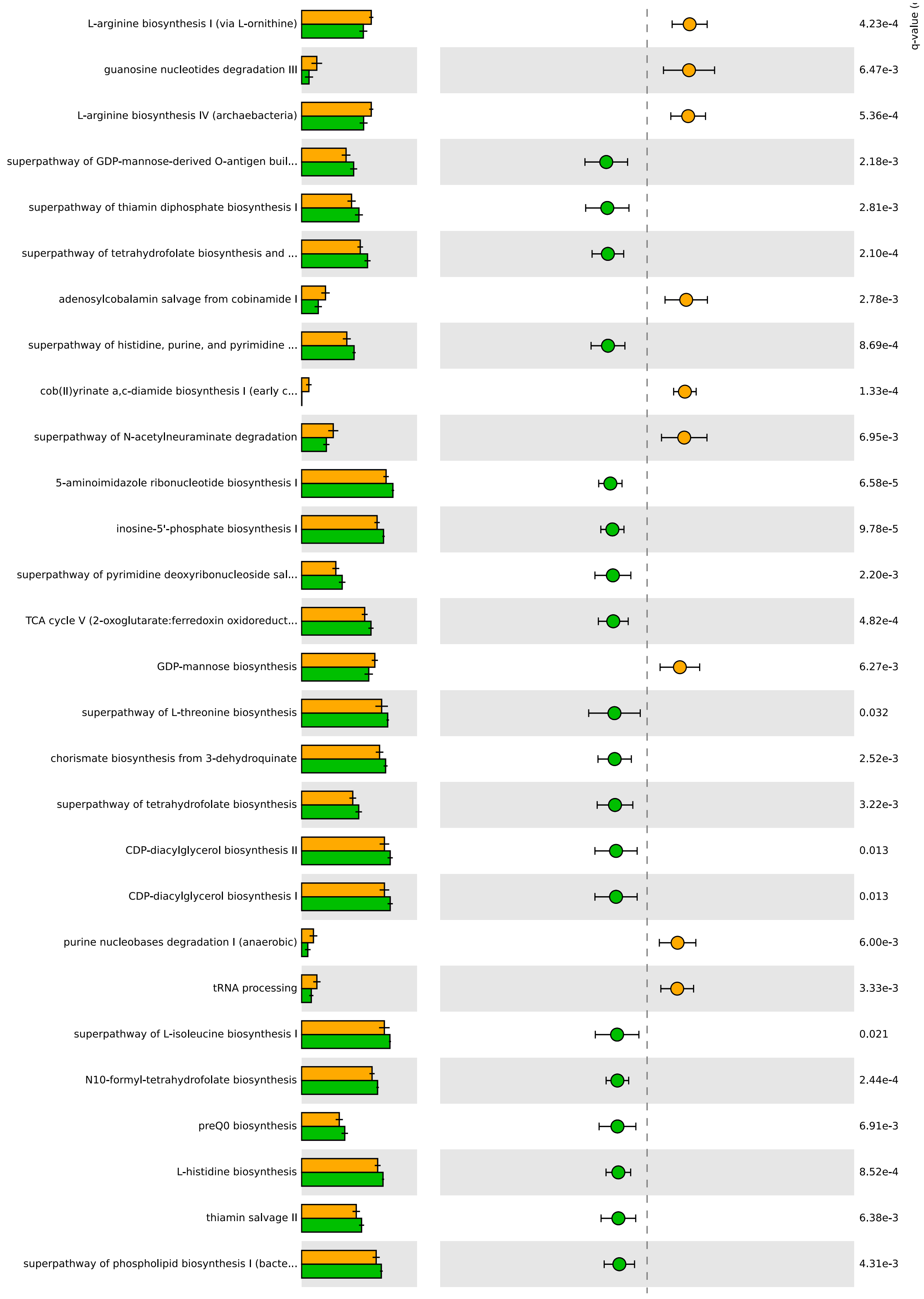
L-arginine biosynthesis I (via L-ornithine)
guanosine nucleotides degradation III
L-arginine biosynthesis IV (archaebacteria)
superpathway of GDP-mannose-derived O-antigen buil...
superpathway of thiamin diphosphate biosynthesis I
superpathway of tetrahydrofolate biosynthesis and ...
adenosylcobalamin salvage from cobinamide I
superpathway of histidine, purine, and pyrimidine ...
cob(II)yrinate a,c-diamide biosynthesis I (early c...
superpathway of N-acetylneuraminate degradation
5-aminoimidazole ribonucleotide biosynthesis I
inosine-5'-phosphate biosynthesis I
superpathway of pyrimidine deoxyribonucleoside sal...
TCA cycle V (2-oxoglutarate:ferredoxin oxidoreduct...
GDP-mannose biosynthesis
superpathway of L-threonine biosynthesis
chorismate biosynthesis from 3-dehydroquinate
superpathway of tetrahydrofolate biosynthesis
CDP-diacylglycerol biosynthesis II
CDP-diacylglycerol biosynthesis I
purine nucleobases degradation I (anaerobic)
tRNA processing
superpathway of L-isoleucine biosynthesis I
N10-formyl-tetrahydrofolate biosynthesis
preQ0 biosynthesis
L-histidine biosynthesis
thiamin salvage II
superpathway of phospholipid biosynthesis I (bacte...
q-value (
4.23e-4
6.47e-3
5.36e-4
2.18e-3
2.81e-3
2.10e-4
2.78e-3
8.69e-4
1.33e-4
6.95e-3
6.58e-5
9.78e-5
2.20e-3
4.82e-4
6.27e-3
0.032
2.52e-3
3.22e-3
0.013
0.013
6.00e-3
3.33e-3
0.021
2.44e-4
6.91e-3
8.52e-4
6.38e-3
4.31e-3

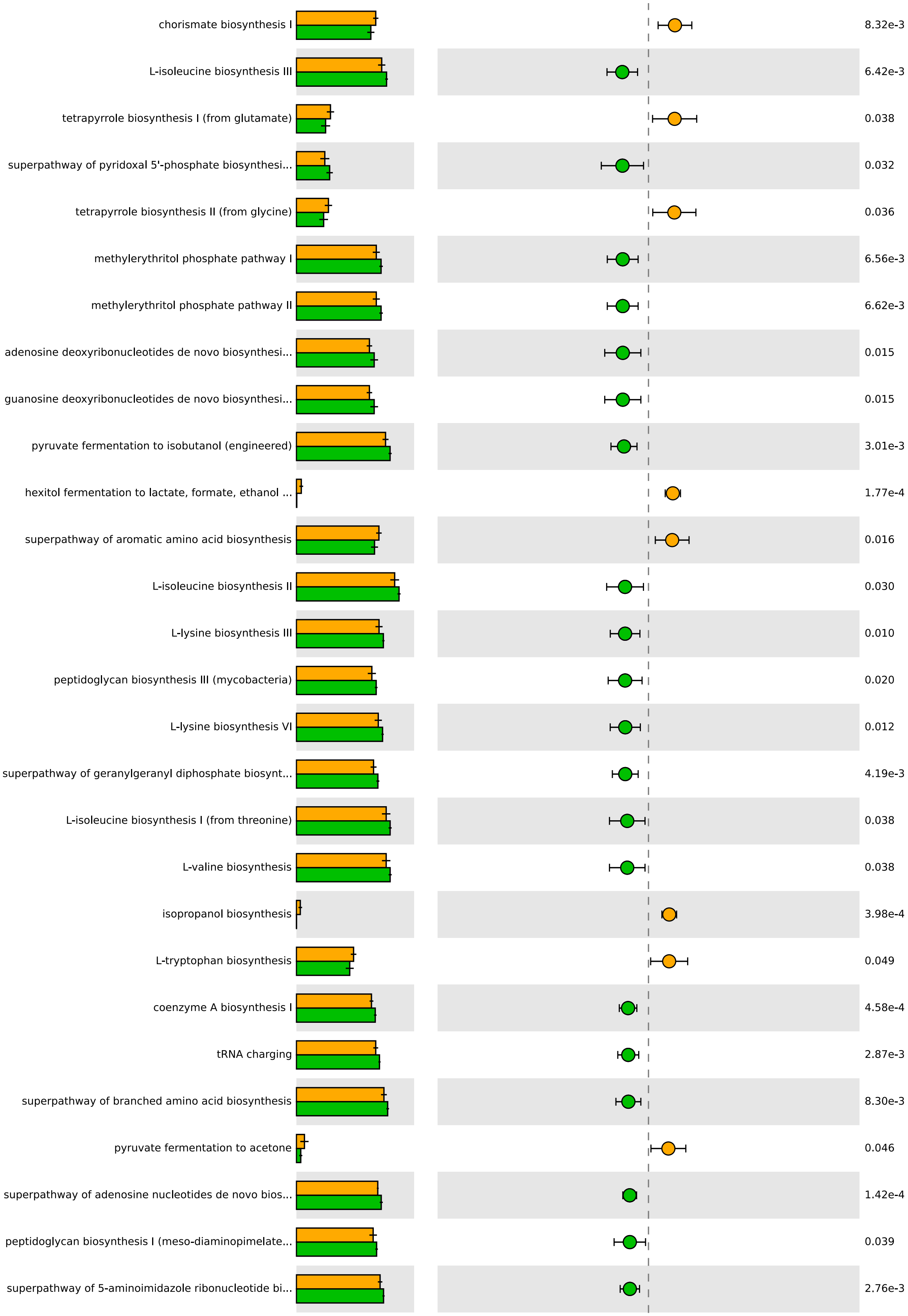

chorismate biosynthesis I
8.32e-3
L-isoleucine biosynthesis III
6.42e-3
tetrapyrrole biosynthesis I (from glutamate)
0.038
superpathway of pyridoxal 5'-phosphate biosynthesi...
0.032
tetrapyrrole biosynthesis II (from glycine)
0.036
methylerythritol phosphate pathway I
6.56e-3
methylerythritol phosphate pathway II
6.62e-3
adenosine deoxyribonucleotides de novo biosynthesi...
0.015
guanosine deoxyribonucleotides de novo biosynthesi...
0.015
pyruvate fermentation to isobutanol (engineered)
3.01e-3
hexitol fermentation to lactate, formate, ethanol ...
1.77e-4
superpathway of aromatic amino acid biosynthesis
0.016
L-isoleucine biosynthesis II
0.030
L-lysine biosynthesis III
0.010
peptidoglycan biosynthesis III (mycobacteria)
0.020
L-lysine biosynthesis VI
0.012
superpathway of geranylgeranyl diphosphate biosynt...
4.19e-3
L-isoleucine biosynthesis I (from threonine)
0.038
L-valine biosynthesis
0.038
isopropanol biosynthesis
3.98e-4
L-tryptophan biosynthesis
0.049
coenzyme A biosynthesis I
4.58e-4
tRNA charging
2.87e-3
superpathway of branched amino acid biosynthesis
8.30e-3
pyruvate fermentation to acetone
0.046
superpathway of adenosine nucleotides de novo bios...
1.42e-4
peptidoglycan biosynthesis I (meso-diaminopimelate...
0.039
superpathway of 5-aminoimidazole ribonucleotide bi...
2.76e-3

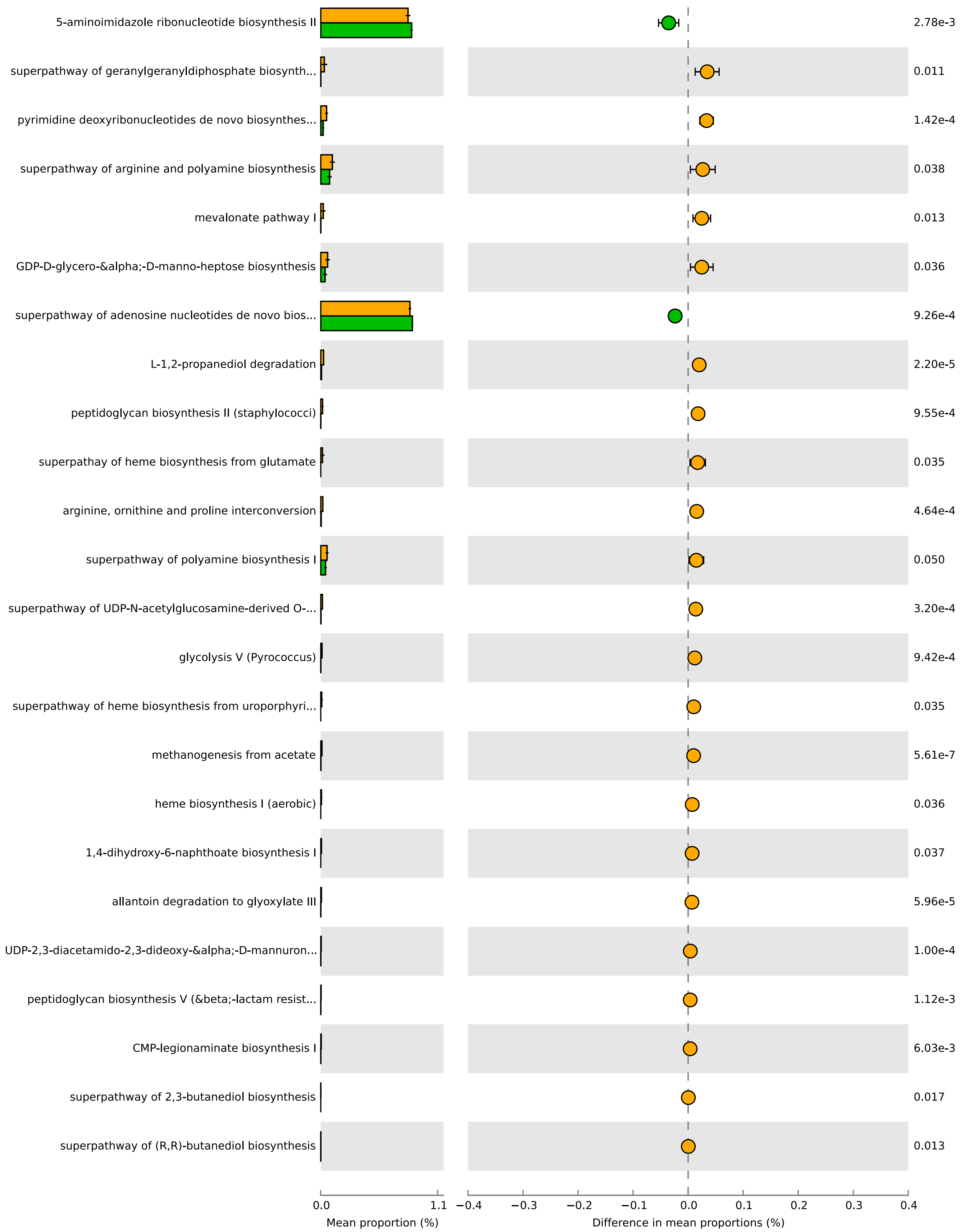

Figure S9. PICRUSt2 results indicating differential predicted functional pathways between the bean-free and the bean-containing diet groups in the male cohort: **(a)** 95 pathways in 0% vs 17.5% bean; **(b)** 131 pathways in 0% vs 35% bean; **(c)** 160 pathways in the 0% vs 70% bean. The bean-free diet (*0%*) is indicated in beige, while the bean-based diet samples (*17.5%; 35%*, and *70%*)—in green. Extended error bar

plot indicating the mean proportion of pathways assigned to each group, difference between them, and corrected p-value (q-value) of each.
